# Microrobotic copper-rich electrochemical interfacing for targeted cancer theranostics in the gut

**DOI:** 10.1126/sciadv.aeb5934

**Published:** 2026-03-13

**Authors:** Junghwan Byun, Siyeon Jang, Yingdan Wu, Jiwoong Choi, Ugur Bozuyuk, Junghyeon Ko, Alp Can Karacakol, Eun Hye Kim, Sungwoo Chun, Amirreza Aghakhani, Seungjun Chung, Jiachen Zhang, Yoosoo Yang, Metin Sitti

**Affiliations:** ^1^ Electronic and Hybrid Materials Research Center, Korea Institute of Science and Technology, Seoul 02792, Republic of Korea.; ^2^ Physical Intelligence Department, Max Planck Institute for Intelligent Systems, Stuttgart 70569, Germany.; ^3^ Institute for Biomedical Engineering, ETH Zurich, Zurich 8092, Switzerland.; ^4^ State Key Laboratory of Robotics and Systems, Harbin Institute of Technology, Harbin 150001, China.; ^5^ Zhengzhou Research Institute, Harbin Institute of Technology, Zhengzhou, Henan 450000, China.; ^6^ Medicinal Materials Research Center, Korea Institute of Science and Technology, Seoul 02792, Republic of Korea.; ^7^ Department of Immunology, School of Medicine, Kyungpook National University, Daegu 41944, Republic of Korea.; ^8^ Department of Integrative Biotechnology, Sungkyunkwan University, Suwon 16419, Republic of Korea.; ^9^ Department of Electronics and Information Engineering, Korea University, Sejong 30019, Republic of Korea.; ^10^ Department Institute of Biomaterials and Biomolecular Systems (IBBS), University of Stuttgart, Stuttgart 70569, Germany.; ^11^ School of Electrical Engineering, Korea University, Seoul 02841, Republic of Korea.; ^12^ Department of Biomedical Engineering, City University of Hong Kong, Hong Kong SAR China.; ^13^ School of Medicine and College of Engineering, Koç University, Istanbul 34450, Turkey.

## Abstract

The exquisite spatiotemporal regulation of drug biodistribution is paramount for optimal targeted cancer theranostics. Robotic ingestible devices promise to reinvent the way drugs interact with gastrointestinal (GI) tissues and malignant tumors. However, no study has yet demonstrated theranostic functions beyond merely conveying synthetic drugs. Here, we present an orally administrable, functionally integrated soft microrobot capable of localized therapeutic regulation of copper (Cu)–dependent cell death mechanism, termed “cuproptosis” within GI tumors. By leveraging the interplay of mechanical, electrical, and biochemical functions, the robot actively targets and grasps the tumor and generates anticancer Cu-rich electrochemical interfacing with the targeted tumor microenvironment. This tumor-robot interface, characterized by in situ generated electric multipole fields and on-demand burst Cu^2+^ ion release, induces ~10^4^-fold increase in local concentration of Cu^2+^ ions, and drives their dense accumulation and directed infiltration for effective cuproptotic cancer treatment, with tumor penetration capabilities far beyond those of passive diffusion. Demonstrations of minimally invasive, long-range tumor targeting in porcine organs and mouse tumor eradication in vivo demonstrate the translational potential of our approach as microrobotic theranostic platforms for targeting GI cancer.

## Teaser

Microrobotic electrochemical interfacing enables localized therapeutic regulation of cuproptosis within gastrointestinal cancer.

## INTRODUCTION

Localizing drug distribution with optimal dose control is of essential interest to the arena of targeted cancer therapy (*1*–*4*). For clinical translation, the overarching goal is to regulate the biological fate—delivery, tumor penetration, and tumor cell internalization—of administered medicines with high spatiotemporal precision (*1*, *3*). This requires unique pharmacokinetic alterations that manifest extremely skewed biodistribution towards the target site of action. In systemic circulation, however, the sharp localization of pharmaceutical substances is generally inhibited either in the spatial or temporal domain due to intrinsic physiological factors including hemodynamic dominance over transport route selection, homeostatic clearance that normalizes blood plasma (e.g., opsonization by the mononuclear phagocyte system), and complex stochasticity involved in extravasation (*3*–*5*). A quarter century of progress in pharmaceutical nanotechnology has found success in pharmacokinetic perturbation regarding circulation half-life (*6*–*8*), vascular or interstitial permeability (*9*–*14*), and binding affinity (*15*–*18*), yet their enduring effectiveness for clinical use is still controversial (*5*, *19*, *20*). A consensus is developing that diffusion-limited passive transport has inherent limitations in achieving sharp localization and deep tissue penetration (*3*, *4*, *21*–*23*), even when combating tumors arising in the gut, the most accessible regions.

Robotic ingestible devices have the potential to redefine the way drugs interact with gastrointestinal (GI) cancer (*24*, *25*). Preclinical technologies—such as mucus adhesion (*26*), mucus clearing (*27*), needle-based injection (*28*–*30*), and microjetting (*31*, *32*)—foster efficiency in GI drug delivery. Harnessing adaptive actuation and associated mobility is particularly beneficial in performing localization and subsequent pharmacokinetic alteration of orally administered biologic agents within the GI environment, as exemplified by magnetic soft miniature robots (*33*–*36*), origami robots with a permanent magnet (*37*), capsulized algae motors (*38*), and biohybrid cellular microrobots (*39*). However, strategies for enhancing drug loading capacity, active sustainable release, and deep tumor penetration have been largely left out of the design rationale.

**Fig. 1.**
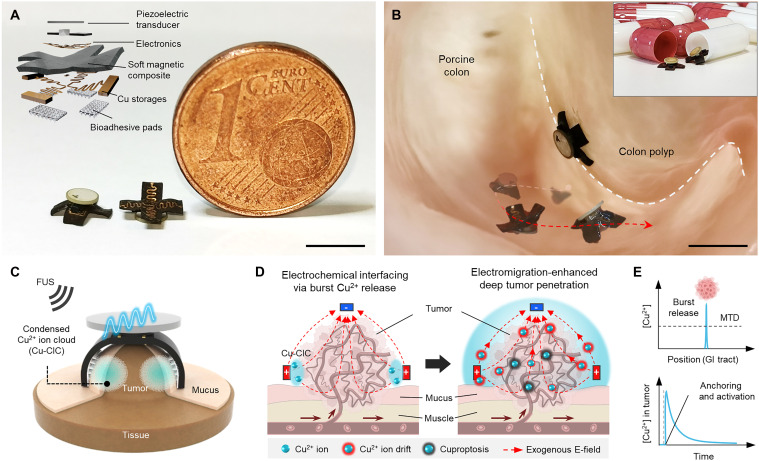
Design and working principle of the microrobot. (**A**) Design of the microrobot. Inset image: an extended-view schematic of the microrobot. (**B**) Photograph of the microrobot capable of deployment, locomotion, on-demand anchoring, and cuproptosis-based cancer treatment in the GI lining. Inset image: edible capsules encasing microrobots for initial deployment to the specified organ through oral administration. (**C**) Microrobot-mediated Cu^2+^-rich electrochemical interfacing of the tumor that drives locoregional up-regulation of Cu^2+^ concentration in the form of Cu-CICs upon wireless FUS signals. (**D**) Cancer-treating mechanism of M-cuproptosis. (**E**) Microrobot-mediated, unique Cu^2+^ pharmacokinetic profiles (see fig. S1 for comparison with conventional drug delivery). MTD denotes “maximum tolerated dose”. Scale bars, 5 mm. Illustrations software credit: Microsoft PowerPoint, Blender.

Here, we report an ingestible, functionally integrated soft microrobot capable of spatiotemporal regulation of copper (Cu) pharmacokinetics within GI tumors via Cu^2+^-rich electrochemical interfacing. As opposed to passive, diffusion-limited drug delivery, the microrobot directly targets GI tumors via magnetically driven active mobility (Fig. 1, A and B) and leverages a subtle interplay between Cu and electric multipole fields to generate peritumoral Cu^2+^-dependent electrochemical interface in situ (Fig. 1C). Ion kinetics within this interface are primarily governed by electromigration rather than passive diffusion, thereby facilitating exclusively localized biodistribution, deep tumor penetration, and the associated anticancer therapeutic outcome through the Cu^2+^-dependent cell-death pathway, termed “cuproptosis” (Fig. 1, D and E, and fig. S1) (*40*).

## RESULTS

### Design and working principle of the microrobot

The microrobot consists of a soft magnetic composite, electronics, bulk Cu storages, and bioadhesive pads (Fig. 1A and figs. S2 and S3). The radial magnetization profile of the soft magnetic body facilitates the pop-up and rolling modes of locomotion (Fig. 1B and fig. S4, A and B). The inclusion of the Cu serpentine trace layer increases the effective bending stiffness of the robot’s legs by several orders of magnitude, thereby allowing the robot to demonstrate its effectiveness in negotiating wet, sticky, and dynamic topographic features of the GI lining by adaptive shape morphing (fig. S4, C to G, and movie S1). Upon application of external focused ultrasound (FUS) signals, the embedded electronics shapes the waveform of electric potential (Φ) in the range that can in situ activate the electrochemical dissolution of solid-state Cu (Fig. 1C and figs. S5 to S8). The intimate interplay between the Φ-induced, spatially coordinated “electric multipoles” and electrochemically released Cu^2+^ ions leads to the spontaneous formulation of highly dense, anticancer Cu^2+^ ion clouds—termed, condensed ion clouds of Cu^2+^ (Cu-CICs)—by which the huge concentration gap across the tumor microenvironment promotes excess intracellular accumulation and accordingly fuels the copper-dependent cell-death pathway, namely, cuproptosis (Fig. 1, D and E) (*40*). Hereafter, we term this microrobot-driven Cu^2+^ release, transport, and tumor treatment mechanism as “microrobotic cuproptosis (M-cuproptosis).”

### Cu-CIC dynamics

**Fig. 2.**
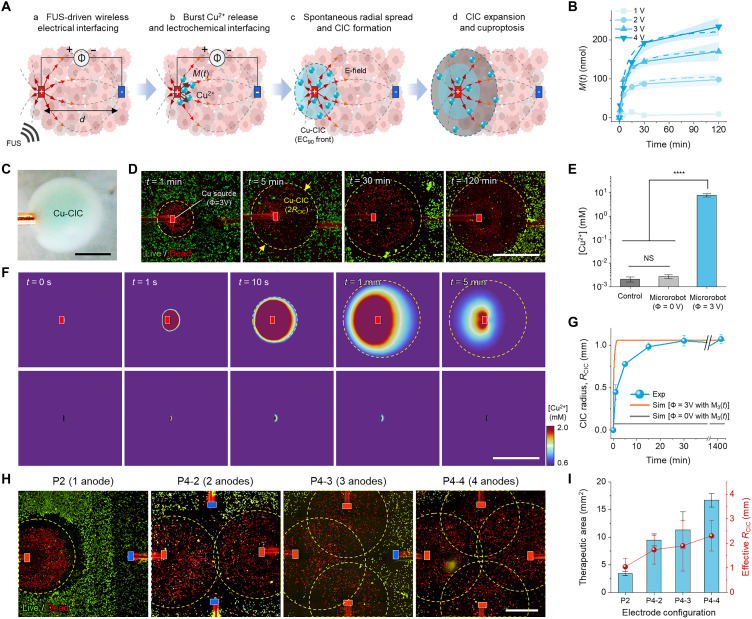
Cu-CIC dynamics. (**A**) Schematic illustration of key phenomenological steps of M-cuproptosis. (**B**) Cu^2+^ production profiles as a function of an applied potential (Φ) by electrochemical dissolution of bulk, solid-state Cu. (**C**) Optical image of the Cu-CIC generated at Φ = 3 V. (**D**) Live/dead fluorescent images of the evolution of Cu-CICs on HT-29 monolayer cancer cells. The boundaries of Cu-CICs are defined by EC_90_ (≈0.6 mM) so that we can identify the Cu-CIC area as highlighted by yellow dashed circles. (**E**) In vitro characterization of local Cu^2+^ production performance of the microrobot at Φ = 3 V (*n* = 4). Statistical analysis was performed using one-way analysis of variance (ANOVA), ****P < 0.0001. (**F**) Numerical results of Cu-CIC dynamics at Φ = 3 V under the continuous cell-ion reaction (that is, intracellular Cu^2+^ uptake) condition expressed by *R*(*c*) = −*kc*, where *k* is the first-order rate constant for passive diffusion (see Materials and Methods, fig. S11, and note S1 for details). (**G**) Numerical and experimental analysis of *R*_CIC_ evolution during M-cuproptosis (Φ = 3 V at *d* = 3 mm). The strong correspondence of *R*_CIC_ between simulation and experimental results, together with ~90% lethality of Cu-CICs as shown in Fig. 4A, justifies the definition of *R*_CIC_ as the EC_90_ front within which [Cu^2+^] ≥ EC_90_. (**H**) Scalable Cu-CIC formation with an increasing number of anodes. (**I**) Characterization of effective therapeutic area according to the electrode configuration as described in (H). All data are means ± SD. Scale bars, 1 mm. Illustrations software credit: Microsoft PowerPoint, Blender.

M-cuproptosis is underpinned by the microrobot-mediated Cu-dependent electrochemical interfacing of tumors that features uniquely regulated ion release and transport mechanisms. In the absence of circulating blood flow, local factors—such as concentration (*c*, denoting [Cu^2+^] in this study), diffusivity (*D*), and electric field (E-field, *E* = ∇Φ)—dominate ion kinetics over conventional pharmacokinetic parameters (Fig. 1D). A minimal model can be constructed by combining the electrochemical Cu^2+^ production model with the reactive drift-diffusion kinetics (Fig. 2A), which is well delineated by the Nernst-Planck equation (see note S1)

$$\frac{\text{∂}c}{\text{∂}t}=\nabla \cdot \left(D\nabla c+\frac{\mathrm{zeD}}{k_{B}T}\mathrm{cE}\right)+\mathrm{kc}$$ (1)

where *z* is the valence of ions, *e* is the elementary charge, *k*_B_ is the Boltzmann constant, *T* is the absolute temperature, and *k* is the first-order reaction rate constant. The Michaelis-Menten reaction kinetics was used to establish a mathematical model of the electrochemical Cu^2+^ production, M(*t*) (or *c*) (Fig. 2Ab; see note S2). The relevance of M(*t*) function was experimentally verified by colorimetric absorbance spectroscopy with the best fitting curves (Fig. 2B). The results also reveal that M(*t*) curves are valid only under the onset condition (Φ > 1.4 V) and are widely tunable as a function of Φ, time duration, and the number of anodes (fig. S6; see note S2).

A pair of Cu sources with potential difference (Φ) act like an electric dipole separated by a distance of *d* (Fig. 2Aa and fig. S8). The interplay between anodic dissolution and singularities in the electric multipole fields—that is, |*E*| → ∞ near the boundaries of Cu sources—provokes instantaneous radial spreads of Cu^2+^ ions (Fig. 2A, b and c) and consequently formulates Cu-CICs wherein local Cu^2+^ concentration increases by a factor of ~10^4^ (Fig. 2, C to E). Notably, we defined the physical boundary of Cu-CICs by the critical concentration for 90% maximal effective cuproptotic cell death, EC_90_ (≈0.6 mM for human colorectal adenocarcinoma cell lines, HT-29) (*41*–*43*), to better relate their therapeutic implication (Fig. 2D and fig. S9). The central role of exogenous E-fields in Cu-CIC dynamics was also verified by in vitro tests, which incorporated independent control over E-field factors at the electrochemical interface (fig. S10). The sequence of kinetic phenomena unique to M-cuproptosis, as shown in Fig. 2A, was equally observed in the simulation (Fig. 2, F and G, and fig. S11), where we used the dynamic model that combines Eq. 1 with the M(*t*) model and the first-order cellular uptake model that was built upon the Goldman-Hodgkin-Katz flux equation (see note S1) (*44*–*46*). The strong correspondence of the radius of Cu-CICs, *R*_CIC_, between experiments and the numerical estimates for a broad range of treatment duration, Φ, and *d* verified the effectiveness of our analytical method (Fig. 2G and fig. S11, C and D).

Cu-CIC dynamics is readily scalable by means of changes in anode size (fig. S12) and configuration (Fig. 2, H and I): The arrangement of Cu anodes modulates the E-field distribution, and the increase in their cross-sectional area and number leads to the expansion of effective therapeutic area, thereby providing a design cue that we use to create microrobots that treat tumors of clinically relevant sizes. Considering that M(*t*) is linearly proportional to Cu area at a given Φ, the scalability of M-cuproptosis can be further enhanced by increasing the cross-sectional area of each Cu anode (fig. S12).

### Tumor penetration dynamics

**Fig. 3.**
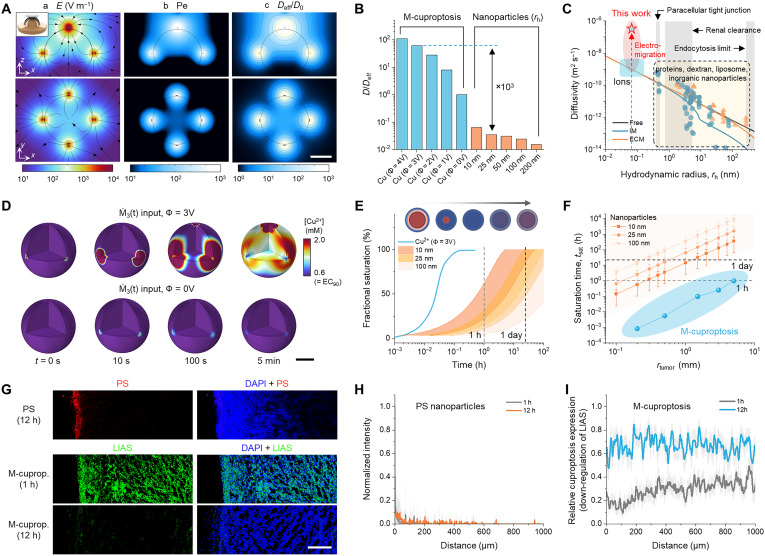
Tumor penetration dynamics. (**A**) Simulation results of E-field (a), *Pe* (b), and *D*_eff_ of Cu^2+^ ions (c) at Φ = 3 V in the peritumoral region (*r*_tumor_ = 1.45 mm). (**B**) Integrative data analysis of normalized effective diffusivity (*D*_eff_/*D*_0_) for M-cuproptosis in comparison with diffusion-limited spherical nanoparticles of clinically relevant sizes [hydrodynamic radius (*r*_h_) = 10 to 200 nm]. (**C**) Plot of diffusivity versus *r*_h_ based on the theoretical models and experimental data. M-cuproptosis, featured by the interplay of high intrinsic diffusivity of Cu^2+^ ions, homogenized diffusive property within neoplastic tissues, and electromigration-dominated kinetics exhibits the potential benefit for tumor penetration by a factor of ~10^3^ to 10^5^ (see note S4 for details). (**D**) Time-evolving spatial distribution of Cu^2+^ ions within the spherical core-shell tumor model (ϕ_ECM_ = 0.25) during M-cuproptosis at Φ = 3, 0 V. See fig. S13 [(F) to (H)] for details on the propagation behavior of EC_90_ fronts and the corresponding local pharmacokinetic profiles. (**E**) Fractional tumor saturation behavior of Cu^2+^ ions (Φ = 3 V) in comparison with nanoparticles (*r*_h_ = 10, 25, and 100 nm). (**F**) *t*_sat_ versus *r*_tumor_ curves for M-cuproptosis in comparison with diffusion-limited nanoparticles. (**G**) Fluorescence images showing the diffusion of PS nanoparticles in the tumor tissues and the expression of cuproptosis through the depletion of LIAS marker proteins over 12 hours. (**H**) Fluorescence intensity distribution of 25-nm polystyrene (PS) nanoparticles as a function of distance (*n* = 3). (**I**) Relative expression profile of cuproptosis as a function of distance (*n* = 3). Scale bars are 1 mm [(A) and (D)] and 200 μm (G).

To evaluate the tumor penetration performance and potential therapeutic outcome involved in M-cuproptosis, we investigated the Peclet number (Pe) for the spherical core-shell tumor model as described in fig. S13A, which reads as

$$\text{Pe}=\frac{\mathrm{uL}}{D}=\frac{\text{zeEL}}{k_{B}T}$$ (2)

where the drift velocity *u* = μ*E* with μ given by the Einstein-Smoluchowski equation, the characteristic length *L* is set to be *d*, and diffusivities (*D*) of tumor interstitial matrix (IM) and extracellular matrix (ECM) are theoretically estimated for a broad continuum of sizes [i.e., hydrodynamic radius (*r*_h_)] based on the effective medium theory with dual-scale cylindrical pore models (*47*) and the modified Brinkman model (*48*), respectively (Fig. 3A, a and b; see note S4). The Pe analysis results show that about 70% of the total tumor volume with a radius (*r*_tumor_) of 1.5 mm exhibits Pe > 10 at Φ = 3 V, supporting that the major transport mechanism of Cu^2+^ ions in M-cuproptosis is electromigration rather than pure diffusion (Fig. 3Ab and fig. S13, B and C). Considering the structural feature of tumor microvasculature, the Taylor-Aris dispersion relation allowed us to further extend the expression of *D* under the electromigration effect, as expressed by *D*_eff_ = *D*_0_(1 + Pe^2^/48), where *D*_0_ is the ordinary diffusivity (*49*). The resultant heatmap and integrative data analysis of *D*_eff_, as shown in Fig. 3 (A to C), suggest that when compared to conventional nanoparticles of clinically relevant sizes (*r*_h_ = 10 to 200 nm), Cu^2+^ ion potentially carries enormous therapeutic benefits for tumor penetration due to its intrinsic diffusivity (by a factor of 10^2^ to 10^3^), negligible steric hindrance (by a factor of 10^1^ to 10^2^), and electromigration-dominated ion kinetics (by a factor of ~10^2^).

We verified this estimation by investigating local Cu^2+^ pharmacokinetics in the tumor. Numerical results show that the dynamics of Cu^2+^ ions in M-cuproptosis are dictated by the E-fields that guide them into the tumor core, whereas the diffusion-limited kinetics of Cu^2+^ ions (Φ = 0 V)—assumed to be generated equally by M_3_(*t*)— are insufficient to compensate for the depletion due to the first-order reaction of intracellular uptake (Fig. 3D and fig. S13). The kinetic profile of moving EC_90_ fronts says that, at Φ = 3 V, the treatment duration required to saturate moderate (*r*_tumor_ = 1.45 mm; volume ~ 12.7 mm^3^, which best suits the Cu configuration with *d* = 3 mm) and even larger tumors (*r*_tumor_ = 5 mm; volume ~ 520 mm^3^) falls within the range of 0.1 to 1 hour (fig. S13E). This tumor penetration performance is particularly noteworthy when compared to biomolecules of clinically relevant sizes, given their marked discrepancy in the characteristic timescale, *t*_sat_, for tumor saturation (Fig. 3, E and F). For effective comparison, the shrinking core model (SCM) was adopted for nanoparticles (*50*), which were assumed to be bound to the tumor surface (*r*_tumor_ = 1.45 mm) under the condition of constant surface concentration (Fig. 3E, inset; see note S5). In-depth analyses on *t*_sat_ and *t*_clear_ (timescale for clearance) reveal that *t*_sat_ > 24 hours and clearance modulus Γ (= *t*_sat_/*t*_clear_) falls within the range of 10^1^ to 10^3^ ≫ 1 for nanoparticles treating the tumors of *r*_tumor_ > 1 mm, whereas *t*_sat_ < 1 hour for M-cuproptosis at Φ = 3 V, validating the therapeutic effectiveness in terms of tumor penetration (Fig. 3F and fig. S14; see note S5).

To experimentally validate the model study, we visualized and analyzed the spatial distribution of polystyrene (PS) nanoparticles and cuproptosis expression in tumor tissues. The tumors were immersed in fluorescent PS nanoparticle solutions of sufficient concentration so that the diffusion condition meets the central assumption of the SCM, while Cu^2+^ ions were electrochemically released in situ by the P4-4 configuration at Φ = 3 V. Fluorescent imaging demonstrated that nanparticle diffusion was limited to the tumor periphery, having a diffusion length of <50 μm, which is in good agreement with the previous study (*13*) (Fig. 3, G and H, and fig. S15, A and B). By contrast, the marked depletion in the fluorescence intensity of lipoyl synthase (LIAS)—the representative marker protein involved in the cuproptosis pathway (*40*)—across the tumor sections attests to the high tumor penetration capability of Cu^2+^ ions and the concomitant vigorous progression of cuproptosis due to elevated Cu^2+^ levels (Fig. 3, G and I, and fig. S15, C and D).

### In vitro therapeutic efficacy

**Fig. 4.**
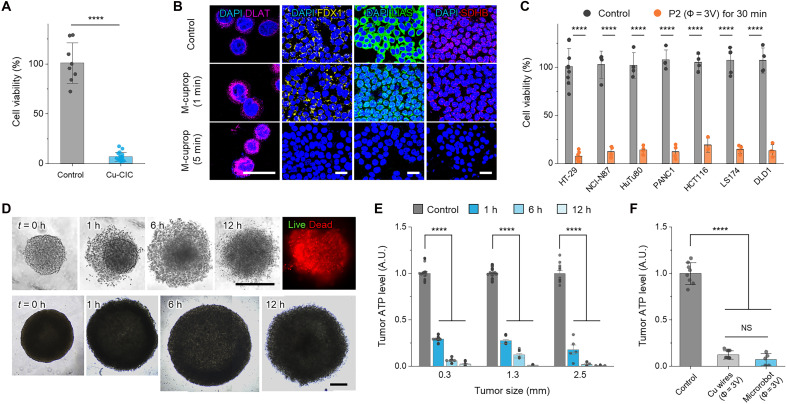
Experimental verification of anticancer efficacy in vitro. (**A**) Anticancer potency of Cu-CICs, formed by different duration and Φ, for HT-29 monolayer cells (*n* = 18). (**B**) In vitro immunofluorescence imaging of key biomarker proteins (DLAT, FDX1, LIAS, and SDHB) involved in the cuproptosis process. (**C**) Viability of various GI cancer cell lines, including HT-29, NCI-N87 (human gastric cancer), HuTu80 (human duodenal cancer), PANC-1 (human pancreatic cancer), HCT116, LS174, and DLD1 (human colorectal cancers), after treatments with M-cuproptosis (*n* ≥ 4). (**D**) Optical images and the corresponding live/dead assay results (top right) of HT-29 tumor spheroids with different sizes (0.3 mm for top and 1.3 mm for bottom) after treatment with 0, 1, 6, and 12 hour of M-cuproptosis. (**E**) In vitro adenosine triphosphate (ATP) assay results for HT-29 tumor spheroids (0.3, 1.3, and 2.5 mm) with increasing treatment duration (*n* = 6 to 16). (**F**) Comparison of antitumor efficacy of 6-h M-cuproptosis fueled by Cu wires (diameter = 150 μm, P4-4 configuration) and the microrobot for 1.3-mm tumor spheroids (*n* = 5 to 10). All data are means ± SD. All statistical analyses were performed using one-way analysis of variance (ANOVA). ****P < 0.0001. Scale bars, 50 μm (B) and 500 μm (D). A.U., arbitrary unit.

The definition Cu-CICs led us to assess the anticancer efficacy of M-cuproptosis by the Cu-CIC area (or volume). In vitro live/dead staining experiments for M-cuproptosis–treated HT-29 monolayer cancer cells provided clear evidence of the kinetic phenomena, such as time-dependent expansion as shown in Fig. 2D, and vigorous killing activity of Cu-CICs (Fig. 4A).

To investigate whether the Cu-CIC-induced cytotoxicity originated really from cuproptosis, we performed immunofluorescence imaging on four representative marker proteins involved deeply in the cuproptosis pathway, such as dihydrolipoamide *S*-acetyltransferase (DLAT), adrenal ferredoxin (FDX1), LIAS, and succinate dehydrogenase complex iron-sulfur subunit B (SDHB) (*40*). The results suggest that M-cuproptosis induced down-regulation of FDX1, LIAS, and SDHB, as well as the resultant DLAT oligomerization (Fig. 4B). This proves that M-cuproptosis shares the same mechanism with cuproptosis, distinct from other existing locoregional therapies using E-fields, such as radiofrequency/microwave ablation (*51*), electrochemical therapy (*52*), irreversible electroporation (*53*), and tumor-treating fields (*54*) (see note S6). Moreover, the similar viability response of seven different human GI cancer cell lines under 30-min M-cuproptosis treatment verified that M-cuproptosis is nonselective and potentially effective for nearly all GI cancers (Fig. 4C).

To explore whether the M-cuproptotic treatment is scalable and effective enough to combat primary features found in solid tumors, we studied antitumor activities for HT-29 tumor spheroid models in vitro. Optical and three-dimensional (3D) confocal fluorescence imaging showed that the treatment potentiated death of individual cancer cells and weakening of integrins and probably tight junctions, eventually disrupting the tumor structure (Fig. 4D). An adenosine triphosphate (ATP)–based luminescent viability assay provided evidence of pronounced antitumor effects that treatments for over 6 hours had a tumor-killing efficacy of >95% for all spheroid groups with a wide range of sizes (0.3 to 2.5 mm), and those with relatively short duration (<1 hour) also exhibited >80% (Fig. 4E). In vitro testing using microrobots finally confirmed that the M-cuproptotic effect proved by Cu wires was equivalent within the margin of statistically insignificant deviation (*P* = 0.1805) to that of the real microrobot activated by wireless FUS signals, thereby validating the rigor of all the above characterizations (Fig. 4F and Materials and Methods).

### Tumor targeting and anchoring

**Fig. 5.**
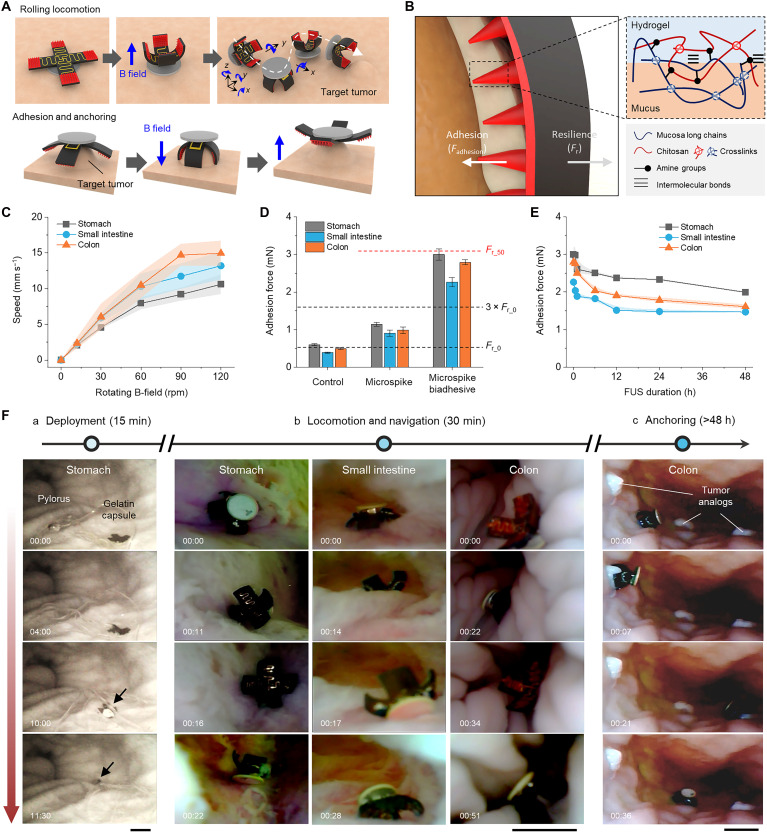
Tumor targeting and anchoring ex vivo. (**A**) Schematic illustration of the strategies for magnetically driven rolling locomotion, adhesion, and anchoring to the target GI tumor protrusion. (**B**) Schematic illustration of the anchoring mechanism. The inset image shows the chitosan-based adhesion principle of the bioadhesive coated onto the micro-spike patterns. (**C**) Locomotion speed characterization in the GI lining as a function of the frequency of alternating magnetic fields (movie S2). (**D**) Adhesion characterization with the GI tissues. (**E**) Long-term sustainability characterization of microrobot anchoring onto the GI tissues under the continuous FUS condition (frequency: 500 kHz, acoustic pressure: 20 kPa). (**F**) Demonstration of locomotion-driven GI navigation and tumor targeting, performed in ex vivo human-scale porcine GI tissues (movies S3 to S5). All data are means ± SD. Scale bars, 5 mm. Illustrations software credit: Microsoft PowerPoint, Blender.

Ex vivo experiments performed in the human-scale porcine GI model demonstrated the capability of long-distance tumor targeting and sustainable anchoring of the microrobot (Fig. 5). The capsule-mediated oral administration initially helped deliver the microrobot to the organ of interest with minimal human intervention (fig. S16) (*55*). The combination of adaptive magnetic actuation and chitosan-based mucus adhesion (*34*) allowed the microrobot to achieve near-unity delivery efficiency and sustainable retention of Cu for specified locations despite the spanning of physical traits throughout the GI lining (Fig. 5, A and B, and movie S1). An average speed of 3 to 5 body length per second (= 10 to 15 mm s^−1^) suggests that the rolling mode is effective for our robot design in terms of speed, reorientation, and friction within the GI environment compared to other possible gaits (Fig. 5C and movie S2; see note S7) (*33*–*36*, *56*). The tumor anchoring performance was optimized by studying the force balance between covalent bonding at the mucus-pad interface, *F*_adhesion_, and elastic resilience of the robot leg, *F*_r_ (Fig. 5B). The parametric characterization verified that the microrobot with optimal adhesive design (height = 100 μm, density = 25 mm^−2^) could achieve robust adhesion (*F*_adhesion_ > 5*F*_r_) either at rest or under unintended magnetic interference, on-demand detachment, and sustainable anchoring under long-term (48 hours), continuous FUS treatments (Fig. 5, D and E, and fig. S17).

Ex vivo testing of long-distance tumor targeting demonstrated the full scenario of our stratagem in the human-scale GI environment (Fig. 5F). The microrobot, encased in a standard size “0” gelatin capsule (21.2 mm by 7.3 mm), was injected through the esophagus with a sufficient amount of water and deployed onto the stomach surface upon dissolution of the capsule throughout a ~15-min time period (Fig. 5Fa and movies S3 and S4). Endoscopic tracking and ultrasound imaging verified that the robot’s locomotion ability drove itself to maneuver within the stomach, pass through the pylorus into the duodenum, and sequentially traverse the small intestine and colon (Fig. 5Fb and movie S4). The total length of the GI tract attempted to traverse was around 1 m (~20, 10, 40, and 30 cm, respectively, for the stomach, duodenum, small intestine, and colon). Demonstrations of consecutive anchoring for potential multisite tumor treatment (Fig. 5Fc and movie S5) and of adhesion under accelerated test conditions of peristalsis (fig. S18 and movie S6) further highlighted the effectiveness of our approach, in terms of precise position adjustment, and successive and robust localization. This series of tumor-targeting demonstrations verified that the therapeutic strategy of M-cuproptosis is centered on minimizing off-target toxicities through sophisticated GI navigation, localization, and dose control, thereby offering potential clinical advantages over systemic pharmacological approaches.

### In vivo validation and biosafety

**Fig. 6.**
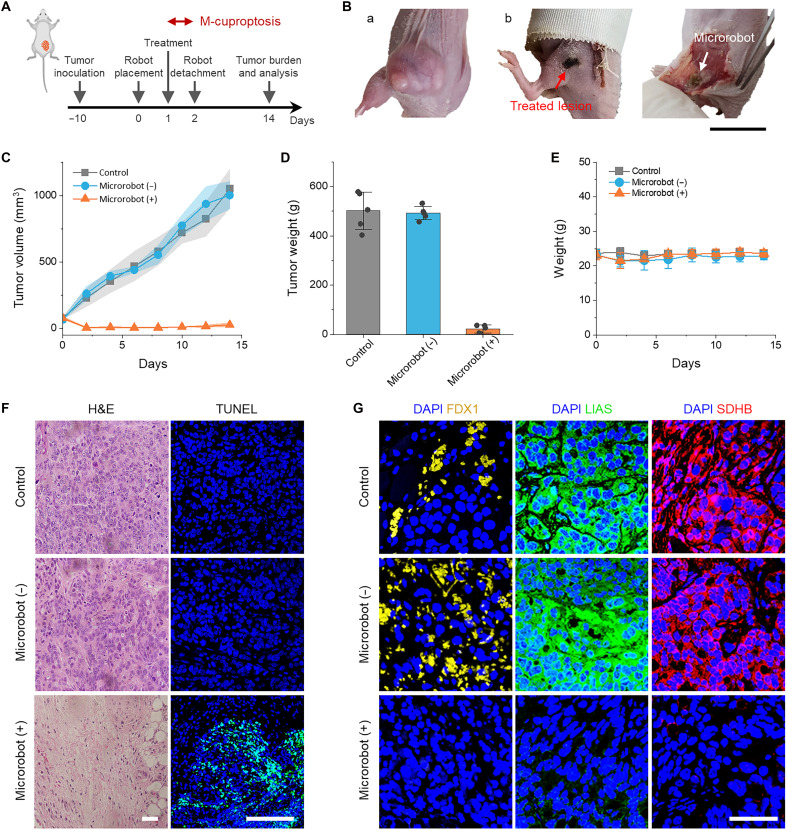
In vivo therapeutic efficacy. (**A**) Experimental protocol for in vivo studies to evaluate in vivo antitumor efficacy of M-cuproptosis using a xenograft mouse tumor model (HT-29). Created in BioRender. Choi, J. (2026) https://BioRender.com/ad31fnz. (**B**) Representative photographs of the control (a) and microrobot (+) group (b) on day 14 posttreatment with M-cuproptosis. (**C** and **D**) Growth (C) and weight (D) of the excised tumors after the indicated M-cuproptosis treatments (*n* = 5). (**E**) Weight curves of mice after M-cuproptosis treatment (*n* = 5). All data are means ± SD. (**F**) Representative histology images stained with hematoxylin and eosin (H&E) (left) and terminal deoxynucleotidyl transferase-mediated deoxyuridine triphosphate nick end labeling (TUNEL) (right). (**G**) In vivo immunofluorescence imaging of marker proteins, including FDX1, LIAS, and SDHB, verifying the cuproptotic pathway. Scale bars, 1 cm (B), 200 μm (F), and 100 μm (G).

In vivo antitumor efficacy of M-cuproptosis was evaluated using a HT-29 xenograft tumor model. Mice were inoculated with HT-29 cancer cells, and on day 14 postinoculation (when the tumor volume reaches ~100 to 200 mm^3^), they were randomly grouped into three—control, “microrobot (−)” without treatment, and “microrobot (+)” with treatment—with microrobots being positioned in the subcutaneous tumor site (Fig. 6, A and B). Negligible changes in tumor volume, weight, and histology for the microrobot (−) group indicate that the subcutaneous microrobot placement with suturing and subsequent long-term contact did not engage in tumor growth (Fig. 6, C to F). In marked contrast, nearly complete tumor eradication was observed in the microrobot (+) group with only a single sequence of 6-hour M-cuproptosis treatment (Fig. 6C and fig. S19). Histological analysis for tumor tissues dosed in vivo confirms structural abnormalities and elevated cancer cell death (Fig. 6F). Immunofluorescence imaging of the Fe-S cluster marker proteins reveals that the expressions of FDX1, LIAS, and SHDB in the microrobot (+) group were notably diminished (Fig. 6G), which again provided evidence of cuproptosis along with the in vitro characterization results shown in Fig. 4B.

**Fig. 7.**
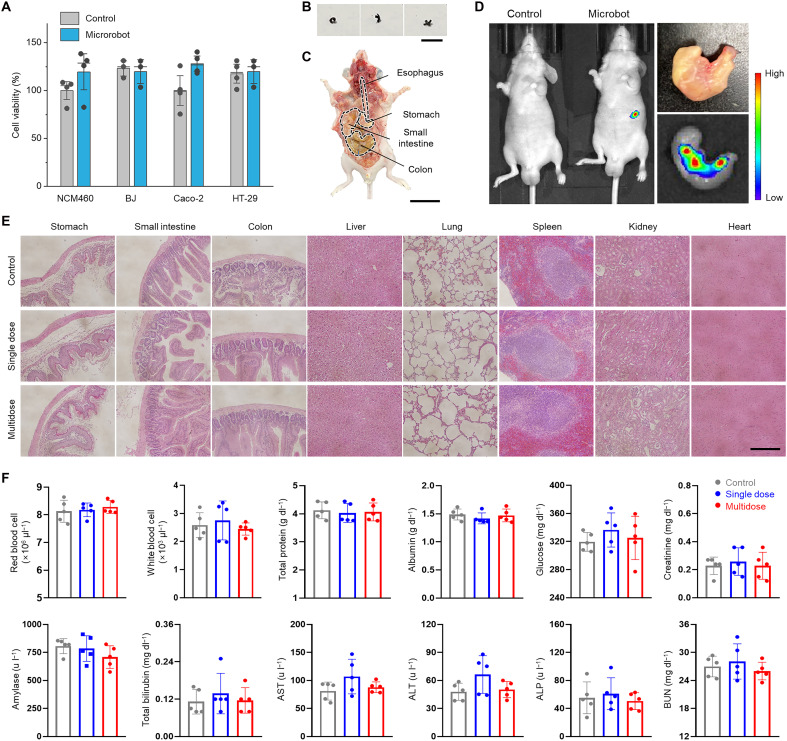
In vivo biosafety. (**A**) In vitro viability tests of NCM460 (human colon epithelial), BJ (human fibroblast), Caco-2 (human colorectal adenocarcinoma), and HT-29 cell lines. (**B**) Sequential image frames of the small-scale microrobot used for in vivo biocompatibility studies. (**C**) Anatomy of the mouse model used for in vivo studies. (**D**) Noninvasive near-infrared fluorescence (NIRF) images of the mouse with orally administered fluorescence-stained microrobot and ex vivo images of the stomach 30 min after administration. (**E**) Representative histological images of various organ tissue sections, stained with H&E, before and after single/multiple (*n* = 3 with 30-min interval) oral administration of the microrobots. (**F**) Comprehensive hematological analysis for control and mice with single/multiple administration of the microrobots (AST = aspartate aminotransferase, ALT = alanine aminotransferase, ALP = alkaline phosphatase, BUN = blood urea nitrogen; *n* = 6). Scale bars, 1 mm [(B) and (E)] and 2 cm (C).

Additional sets of in vitro cell viability and in vivo biosafety tests upon ingestion were performed to explore the potential feasibility of clinical trials (Fig. 7). Given the intrinsic discrepancy in characteristic length between the microrobot (~3 mm in a rolled state) and the mouse gut (~0.58 mm), we used a miniature model of microrobots (length = 500 μm) especially for the tests involving the oral administration process (Fig. 7, B to D). Histological analysis shows that organs associated with digestive system and vitality exhibited no observable inflammation or pathological alteration after multiple sequences of oral administration and excretion (Fig. 7E). Comprehensive hematological studies show that the levels of all types of serum biosafety markers and counts of blood cells remained within the normal range for all experimental groups (Fig. 7F).

## DISCUSSION

The M-cuproptosis establishes a framework to deliver, localize, and infiltrate therapeutic agents into GI tumors with unique pharmacokinetic behaviors that are unattainable by systemic delivery. Our findings show that cupric ion (Cu^2+^) can be revisited as an effective anticancer agent for the following three aspects. First, excess Cu^2+^ ions lead to anticancer activities across diverse cancer cell lines through a programmed pathway of cell death known as cuproptosis (see Fig. 4C)—driven by FDX1-mediated mitochondrial proteotoxic stress (*40*, *57*). Second, the locomotion-driven bulk transport of Cu^2+^ ions in solid form and their burst, controllable release by in situ electrochemical dissolution at specified locations facilitate sharp localization with near-unity delivery efficiency. Last, the dynamic interplay between electric multipole fields, electrochemical dissolution, and reactive drift-diffusion ion kinetics demonstrates the formation and deep tumor penetration of cuproptotic ion-rich clouds, Cu-CICs, which are attributed to very high intrinsic diffusivity of Cu^2+^ ions in tumor tissues, positive charge-assisted promoted transcytosis (*58*, *59*), and electromigration.

Endoscopic access was used in our study to visualize and validate how the robot is deployed, moves, and physically targets GI tumors. In clinical practice, however, endoscopes cannot reliably access past the distal duodenum. Therefore, clinically relevant, noninvasive medical imaging modalities—such as ultrasound (movie S4), x-ray (*60*), photoacoustic (*61*), and fluorescence imaging—need to be used to track and localize the robot in the GI track. Of these, ultrasound-guided autonomous navigation offers the most credible potential to address this translational challenge, as presented in our recent work on data-assisted autonomous ultrasound-guided tracking of miniature robots (*62*). Future work in this direction will give rise to the advanced functionalization and miniaturization of embedded systems that realize imaging-free autonomous GI navigation.

While this study focuses on the development and proof-of-concept of M-cuproptosis, translational relevance should be assessed in terms of targetable tumor morphology and staging, long-term biosafety, and sustainability of treatments. Many early cancers are flat or depressed, and advanced ones often infiltrate the wall and muscularis, such as gastrointestinal stromal tumor, although our ex vivo/in vivo models were limited to discrete protruding nodules. Literature studies on Paris Classification for early GI cancer suggest that 15 to 50% of early gastric cancers and ~90% of early colorectal cancers (ECCs) fall within 0-I(polypoid) and/or 0-IIa(slightly elevated) categories (*63*–*65*), indicating that our microrobot could demonstrate therapeutic effectiveness in most ECCs. In addition, our microrobot (large pad, 0.8 mm by 0.25 mm) is capable of generating Cu-CICs with an effective therapeutic diameter of approximately 8.6 mm (fig. S12B), within which [Cu^2+^] > EC_90_ = 0.6 mM. Considering that the maximum depth of invasion for early-stage cancer (T1 stage) is, by definition, confined to the submucosa, with total depths (mucosa and submucosa) of 2.5 ± 0.8 mm (stomach) and 1.8 ± 0.7 mm (colon) (*66*), this large therapeutic area suggests that the proposed M-cuproptosis strategy could sufficiently cover the entire thickness of the stomach (3.5 ± 0.7 mm) and colon walls (2.9 ± 0.7 mm) and could readily treat various tumor types, including both polypoid and nonpolypoid (sessile, slightly elevated, flat, and depressed) (figs. S20 and S21). Based on this investigation, our approach is expected to be highly relevant for the clinical translation in GI cancers up to T3 disease of the TNM (tumor-nodes-metastasis) classification.

Another critical factor to assess translational relevance is the long-term biosafety of Cu^2+^ ion release. M-cuproptosis reduces its potential clinical risk of copper overload and off-target toxicity by (i) inhibited premature release due to *E*^0^ > 0, (ii) GI locomotion-driven near-unity delivery efficiency and localization, (iii) optimal Cu loading for tumor-specific treatment, and (iv) controlled release by duration control of FUS activation. In particular, the capability of optimal Cu loading and release helps our microrobot to carry the maximum Cu accumulation of ~0.2 mg with an estimated Faradaic efficiency of ~10%, which is only ~2% of the maximum tolerable daily intake of Cu (*67*).

The operational robustness of FUS-driven M-cuproptosis against potential physiological variables (e.g., pH, fluid content, or peristalsis) is underpinned by three engineered redundancies. First, the anchoring stability is guaranteed by the chitosan-based tough adhesive, which maintains high cohesive integrity against peristaltic shear forces and pH variations via dual electrostatic and covalent interfacial bonding mechanisms (*68*). Second, the fluctuations in FUS signals caused by physiological variables are mitigated by the acoustic homogeneity of the GI tract and a sufficient spatial tolerance window of approximately ±2 cm (*69*), within which the local acoustic pressure remains high enough to exceed the 30-kPa threshold required for effective potential generation (see fig. S5, C to E). Last, the applied 3-V dc potential acts as a substantial overpotential that transcends pH-dependent shifts in equilibrium potential defined by the Nernst equation, ensuring the fundamental kinetics of electrochemical Cu/Cu^2+^ burst release regardless of the local acidity or alkalinity of the digestive tract (*70*).

The prospective linkage between cuproptosis and immunotherapy further supports the clinical potential of our approach. Recent studies have reported that cuproptosis can induce immunogenic cell death (ICD) and thereby activate antitumor immune responses in the surrounding TME (*71*–*73*). These findings suggest that cuproptotic tumor cells can trigger immune-mediated clearance not only at the treatment site but also at distant metastatic regions, broadening the therapeutic implications of Cu delivery. Although this ICD-driven mechanism of M-cuproptosis and its potential therapeutic efficacy for advanced GI malignancies (beyond T4) warrant further work, we expect that the proposed platform would provide practical guidance for the clinical translation of targeted cancer therapeutics and help in broadening available options for minimally invasive, personalized targeted therapies.

## MATERIALS AND METHODS

### Microrobot design and fabrication

#### Design consideration

Our previous microrobot designs (*33*–*36*), based on micro-/millimeter-scale magneto-elastomeric composites with programmed ferromagnetic domains, have strength in many aspects such as rapid and precise control, versatility of locomotion modes, and potential biomedical applications. For the specified task such as the navigation of GI terrains characterized by wet, sticky, and grooved surfaces, such a locomotion principle has been found to predominate over other existing approaches. Nevertheless, how this outstanding mobility and targeting ability through the GI lining can aid in the clinical translation of lethal diseases, such as cancer, is poorly understood. To what extent microrobots could provide advanced therapeutic functions is also largely unexplored. Despite the undoubted benefits of installing electronic functionalities onto the microrobot platform, there exists a critical roadblock: When considered from the size perspective—for example, the minimal dimension of commercially available integrated circuit (IC) packages is ≥1 mm by 1 mm (*74*–*76*)—circuit functions that should be defined within a tiny effective area of ingestible microrobots (length <10 mm at most) are fairly limited. Changes in effective stiffness of the microrobots due to the integration of metallic interconnects and bulk ICs are another critical issue for kinetic modeling and locomotion control. In this regard, our key achievement is that we successfully imparted unprecedented therapeutic functions to the microrobot using a compact, miniature, and minimal function design of embedded electronics, without compromising on its forte in mobility (Fig. 1A). To address these design issues, we took the most compact form of the bridge rectifier circuit consisting of four Schottky diodes (two IC packages; 1 mm by 1 mm) and one capacitor (0.6 mm by 0.3 mm) to be connected to the piezoelectric transducer (fig. S5A). The embedded circuit only occupied the total area of <3.6 mm by 1.5 mm, and its function could rectify the wirelessly transmitted FUS signals (500 kHz) to generate stable 3-V dc output signals (fig. S5), which were sufficient to fuel the electrochemical dissolution process of solid Cu sources to initiate M-cupropotosis treatment (fig. S6). In addition, we considered the optimal robot thickness such that the integration of metallic traces (Cu with a thickness of 8 to 25 μm in this study) does not interfere with but, rather, complement the dynamic motion of the microrobot based on the increase in effective bending stiffness (fig. S4 and movie S1).

Another key design criterion includes the maximum tolerance of biological tissues and cellular systems against FUS pressure and the corresponding electric potential (Φ) that the microrobot can generate for M-cuproptosis treatment. We found that the maximum tolerable level of FUS is limited to ~30 kPa, which corresponds to a power density of ~60 mW cm^−2^ (fig. S5, C to E). Five different types of piezoelectric transducers (Physik Instrumente GmbH) were tested, and the results suggest that three among them (*D*2*t*0.25, *D*3*t*0.1, and *D*3*t*0.25, where *D* as diameter and *t* as thickness, with units of mm) met the output voltage level required to activate the electrochemical decomposition of Cu such that *V*_rms_ (root mean square) > Φ_onset_ = 1.4 V (fig. S5; see note S2). Note that the upper bound of the device size (or diameter) is set to be 3 mm because the characteristic length of the microrobot is ~3 mm in its rolled state. Given the saturating effective therapeutic area, in terms of Cu-CIC size for Φ ≥ 3 V (≈8.5 V_pp_) (fig. S9), we chose the transducer design with a diameter of 3 mm and a thickness of 0.25 mm, whose rectified output voltage could reach 3 to 5.5 V within the biologically safe FUS regime (fig. S5E).

#### Fabrication of the microrobot

A soft microrobot body was prepared using a 100-μm-thick magneto-elastomeric composite which consisted of neodymium-iron-boron microparticles (NdFeB, average size of ~5 μm, Magquench GmbH) and Ecoflex 0010 silicone rubber (mixture mass ratio, part A/part B = 1:1, Sooth-On Inc.) in 6:4 weight ratio. To form electric circuits onto both top and bottom surfaces, sequential steps of high-resolution ultraviolet laser patterning (ProtoLaser U3, LPKF) were applied. In detail, Cu foils (thickness of 5, 8, or 25 μm for each purpose and purity of >99.9%; Sigma-Aldrich) were oxygen (O_2_) plasma treated for 2 min with a power of 75 W (Basic Tergeo Tabletop Plasma Cleaner, PIE Scientific LLC). The surface-activated Cu foils were then laminated onto both sides of the magneto-elastomeric composite in a sandwich-like shape via liquid-phase poly(dimethyl siloxane) (PDMS) glue (Sylgard 184, Dow Inc.), followed by thermal curing for 1 hour at 90°C. The top layer circuit was patterned by laser-induced Cu removal, which created 80-μm-wide conductive Cu traces within a compact area of <3.6 mm by 1.5 mm. The Cu-patterned composite film was subsequently cut to obtain the cross-shaped outline of the microrobot body having an end-to-end length of 6 mm and a width of 2 mm. Flipping the robot body upside down, we repeated the same process for the bottom circuit layer where thick Cu storage electrodes were defined. The prepared, electronics-integrated, soft magnetic body was wrapped around a 3D printed cube (edge length of 2 mm) and magnetized by a uniform magnetic field of 1.8 T (Z7 VSM, MicroSense LLC). Afterward, electronic device components—including two Schottky diode arrays (Surface Mount SOT-963, Digi-Key), a capacitor (CAP CER 10 μF 4 V X6S 0402, Digi-Key) and a piezoelectric transducer (Physik Instrumente GmbH)—were bonded at proper positions using silver-filled epoxy adhesive (Ablestik C 990 333, Loctite) and thermally cured for 90 min at 130°C. The top and bottom circuits were electrically connected by through-hole Cu wirings based on the predefined via holes. All of the electrical components and robot body were coated by a 200-nm layer of thermally deposited parylene C (Plasma Parylene Systems GmbH; a parylene coater PDS 2010, Specialty Coating Systems Inc.) to insulate them from the gastric environment. Note that bulk Cu storage electrodes were prevented from the parylene C coating process, being exposed to activate M-cuproptosis upon request.

To prepare the adhesive patches, we fabricated the micro-spike mold using a two-photon polymerization 3D printer (Photonic Professional GT, Nanoscribe GmbH) with a rigid commercial photo resin (IP-S, Nanoscribe GmbH). The micro-spike patch was then made of PDMS (20:1 monomer–to–cross-linker ratio) in a two-step molding method, followed by treatment with a benzophenone solution [20 weight % (wt %) in ethanol, Sigma-Aldrich] to act as a hydrophobic photoinitiator. Subsequently, it was coated with poly(ethylene glycol) diacrylate (Sigma-Aldrich) to serve as a dissipative hydrogel layer, followed by a chitosan-based bioadhesive for tissue surfaces. The preparation of the bioadhesive involved dissolving chitosan (high molecular weight, Sigma-Aldrich) as a bridging polymer and unsulfated *N*-hydroxysuccinimide (98%; Sigma-Aldrich) as a coupling reagent (12 mg ml^−1^) in compound MES buffer (Sigma-Aldrich) at a weight ratio of 2.0 and 0.12%, respectively. Four bioadhesive patches were then bonded onto the microrobot legs at their ends using Ecoflex 00-10. For the ex vivo test, the fabricated microrobot was encapsulated in a standard 21.2 mm by 7.3 mm gelatine capsule (Clear gelatin capsules, EU origin, size 0, Kapselwelt Inc.).

### Experimental characterization

#### Morphological characterization

Scanning electron microscopy (SEM) was used to characterize the cross-sectional morphologies of the soft magnetic composite (fig. S3, A to C), made of PDMS and NdFeB microparticles, bulk Cu storage electrode (fig. S3D), and bioadhesive micro-spike patterns (fig. S3, F and G) using a microscope (Ultra 550 Gemini SEM, Carl Zeiss Inc.). For sample preparation, microrobots before and after M-cuproptosis treatment were sputtered with 20-nm-thick gold by a benchtop sputtering coater (Leica EM ACE600, Leica Microsystems). The imaging mode with an accelerating voltage of 5 keV and an Everhart-Thornley secondary electron detector was used. SEM–energy dispersive x-ray spectroscopy analysis was additionally performed to identify the chemical composition of the microrobot and Cu sources before and after M-cuproptosis treatment) (Figs. 3D and 7A).

#### Magnetic characterization

The magnetic properties of the samples were characterized using a vibrating sample magnetometer (EZ7 VSM, MicroSense LLC) to obtain the magnetic hysteresis (*B*-*H*) curve. The samples were prepared in a circular shape with a 1-mm radius and 150-μm thickness using the same material composition as the robot body, ensuring that they fit within the measurement constraints of the VSM. The samples were mounted on a nonmagnetic sample holder and positioned at the center of the VSM detection coil system. The VSM was calibrated before measurements, ensuring accuracy within the instrument’s sensitivity range. The magnetic field was applied with a maximum field strength of ±1.8 T. The hysteresis loop was recorded by varying the applied magnetic field in a sweep mode between the maximum positive and negative field values at a sweep rate of 50 mT s^−1^. The resulting *B*-*H* curves were analyzed to determine the magnetic permeability and hysteresis losses. All measurements were repeated at least three times to ensure reproducibility, and the mean values were reported (fig. S4D).

#### FUS and electrical characterization

The experimental setup of FUS was built by connecting a function generator (AFG3102C, Tektronix Inc.), a function amplifier (Model 2100HF, Trek Inc.), and a 500-kHz focused single-element transducer probe (H104, SONIC CONCEPTS) (fig. S5B). The function generator produced 500-kHz sinusoidal driving signals with an amplitude of 0.22 V_pp_. The generated signals were amplified 50 times and then transmitted to the FUS probe, immersed in water, to generate mechanical acoustic pressure. To find a FUS focal point and measure acoustic pressure, we used a calibrated needle hydrophone with a tip diameter of 500 μm (NH0500, Precision Acoustic Ltd.). To measure the output signal of the embedded electronics in response to FUS signals, we fixed the microrobot onto a slide glass and precisely positioned it to the focal point underwater. Acoustic pressure and input/output electrical signals from the microrobot were measured in real time by a mixed domain oscilloscope (MDO4024C, Tektronix Inc.). The reliability of the microrobot’s electrical performance was assessed by applying cyclic bending actuation using a universal tensile testing machine (model 5942, INSTRON), during continuous electrical measurement (fig. S5H).

#### Electrochemical characterization

For sample preparation, 35-mm petri dishes were integrated with enamel-coated Cu wires (diameter = 150 μm; Sigma-Aldrich) in four different configurations (P2, P4-2, P4-3, and P4-4; *d* = 3 mm) (Fig. 2H and fig. S6), which represent the spatial layout and therapeutic function of the four-legged microrobot without loss of generality. Next, a poly(methyl methacrylate) (PMMA) well structure (diameter = 8 mm, height = 10 mm) was installed at the target area (diameter, *d*) to contain 200 μl of Dulbecco’s modified Eagle’s medium without phenol red (DMEM, no phenol red; Gibco) solution. The optical imaging of in situ release and time-dependent spatial distribution of Cu^2+^ was performed using a stereo microscope (Stemi 508, Carl Zeiss Inc.), given that aqueous cupric ions (Cu^2+^) exhibit blue in color. Colorimetric absorbance spectroscopy based on the Copper Assay Kit (MAK127, Sigma-Aldrich) and the microplate reader (TECAN) was conducted by measuring the absorbance intensity at 359 nm to measure the quantity of Cu^2+^ as a function of Φ (Fig. 2B) and the number of anodes (fig. S6, F and G). X-ray photoelectron spectroscopy (Theta Probe, Thermo Fisher Scientific) was performed to characterize the chemical component and status of the electro-oxidized Cu samples (fig. S7). The resulting mechanism and by-products during the electrochemical dissolution process were investigated through chemical binding energy of O 1s and Cu 2p peaks in narrow-scan XPS spectra for the samples treated by Φ = 3 V for 1, 5, 10, and 30 min in P2 configuration.

#### Locomotion control and characterization

The locomotion characterization was conducted in the custom-built electromagnetic coil setup (fig. S15E). The customized setup included three pairs of solenoids, enabling the generation of uniform magnetic fields up to 30 mT within a workspace of 2 cm by 2 cm by 2 cm. The system was under the control of an NI compactRIO embedded controller (National Instruments), linked to a PC. For solenoid actuation, a driver board housing eight motor drivers (SyRen 25, Dimension Engineering) was used, powered by a dedicated power supply. Visual monitoring and video recording during the experiments were facilitated by digital cameras (Blackfly S USB3, FLIR Systems) connected to the same PC. To ensure consistency in measurements across each test, the external rotating magnetic field waveforms of various magnitudes and frequencies were preprogrammed using LabVIEW (National Instruments Inc.). Another magnetic actuation system using permanent magnet was used for robot control under medical imaging modalities (fig. S16E). The setup comprised a stepper motor (535-0372, RS Components GmbH) mounted on the *x*-*y* translational motorized stage (LTS300/M, Thorlabs Inc.).

#### Adhesion characterization

The experimental setup for measuring adhesion involved a customized setup as depicted in fig. S17A. The adhesion was measured using a load cell (GSO-25, Transducer Techniques LLC) connected to the probe, where the tests included loading and retracting steps solely related to the vertical motion of the probe via a high-precision piezo motion stage (LPS-65, Physik Instrumente GmbH & Co. KG). During the adhesion test, the probe’s approaching and retracting speeds were set at 50 μm s^−1^, with a preload of 0.1 mN and a 3-s contact time. Tissue samples were periodically prepared from fresh porcine organs to prevent dehydration. In addition, for characterizing adhesion after FUS treatment, patches were affixed to porcine colon tissues within a sealed petri dish, integrated into the FUS treatment setup for the prescribed duration. Adhesion tests commenced immediately after completing the specified treatment duration. The experiments of evaluating the adhesion stability of the microrobot under simulated peristaltic motion were implemented in a customized setup as depicted in fig. S18A. A 3D printed motorized linear actuator (Form 3, Formlabs Inc.) coupled with a servo motor (900-00008, Parallax Inc.) was used to apply cyclic normal forces onto the outer porcine colon wall, where the microrobot was adhered to the inner luminal wall. The cyclic actuation was driven at a frequency of approximately 0.43 Hz, mimicking physiological peristaltic waves (movie S6).

### Simulation

Finite element analysis (FEA) was performed not only to model the dynamic behavior of Cu^2+^ ion transport during M-cuproptosis within the tumor microenvironment but also to evaluate the corresponding therapeutic benefit in direct comparison with diffusion-limited nanoparticles. All FEA simulations were performed with a commercial finite element solver (COMSOL Multiphysics), based on the combination of AC/DC Electric Currents and Chemical Species Transport modules. Two types of geometrical models—a cuboid with a reactive plane boundary condition and a core-shell sphere encased in a cube, both of which were defined by a characteristic length of 3*d*—were used to study Cu-CIC dynamics for monolayer cancer cells (figs. S11 and S12) and 3D spherical tumors (fig. S13). To match with the actual experimental parameters, Cu source electrodes were modeled as a thin disk with a diameter of 150 μm and a thickness of 20 μm, and electric potential (Φ = 0 to 4 V) was applied to only one surface with other surfaces being perfectly insulated. Φ-dependent concentration functions, denoted as M_Φ_(*t*), obtained from the mathematical model of electrochemical Cu^2+^ dissolution (see note S1), were applied as the input boundary condition of [Cu^2+^]. Note that for larger tumors (*r*_tumor_ ≥ 2.5 mm, volume ~ 113 mm^3^), we used Cu sources with bigger cross-sectional area for both simulations and experiments, such that M(*t*) is scaled to βM(*t*) by the area factor β (fig. S12). Diffusivities of species, such as Cu^2+^ ion and nanoparticles, in the core-shell tumor model were obtained from mathematical models as described in note S4. General material properties, such as relative permittivity and electrical conductivity, used for FEA are summarized in table S1.

### Biological experiments

#### Cell lines

Human GI cancer cell lines—including a colorectal adenocarcinoma cell lines HT-29 [American Type Culture Collection (ATCC)], LS174 (DSMZ), and DLD1 (DSMZ); a colorectal carcinoma cell line HCT116 (LGC Standards); a duodenal adenocarcinoma cell line HuTu 80 (ATCC); a pancreatic carcinoma cell line PANC-1 (DSMZ); a gastric adenocarcinoma cell line NCI-N87 (ATCC); an intestinal carcinoma cell line (Caco-2); and a colon epithelial cell line NCM460 (INCELL)—were purchased from each supplier and cultured according to adequate protocols. HT-29, HuTu 80, and PANC-1 cells were cultured in 90% DMEM (Gibco) supplemented with 10% fetal bovine serum (FBS; Gibco), and 1% penicillin/streptomycin (Gibco). LS174, DLD1, and NCI-N87 cells were cultured in 90% RPMI 1640 medium (Gibco) containing 10% FBS and 1% penicillin/streptomycin. HCT116 cells were cultured in 90% McCoy’s 5A medium (Gibco) containing 10% FBS and 1% penicillin/streptomycin. NCM460 cells were cultured in DMEM containing 10% FBS and 1% antibiotic-antimycotic (Gibco). Caco-2 cells were cultured in minimal essential medium (WELGENE) containing 10% FBS and 1% antibiotic-antimycotic. All cell lines were cultivated at 37°C in a humidified 5% CO_2_ atmosphere using 75-cm^2^ cell culture flasks (T-75, Sigma-Aldrich) and used at passage numbers below 20. The detachment of cells was implemented by using trypsin (0.25 wt %)/EDTA solution (Gibco) upon reaching 70% confluency.

#### In vitro cell monolayer sample preparation

For in vitro tests of cancer monolayer models, we used 35-mm cell culture petri dishes (Nunc Cell Culture, Thermo Fisher Scientific Inc.) installed with enamel-coated Cu wires (diameter = 150 μm, Sigma-Aldrich) in four different configurations (P2, P4-2, P4-3, and P4-4; *d* = 1 to 4 mm). Cancer cells (2.20 × 10^5^) were seeded on each cell culture dish on 1 day before the experiments and incubated for 24 hours to stably settle down to the surface of cell culture dishes.

#### In vitro biocompatibility test

To examine the biocompatibility of the microrobot, 0.5 × 10^4^ of HT-29 cells and 1 × 10^4^ Caco-2 and NCM460 cells were seeded in three microrobot-attached 96-well cell culture plates and then incubated in each’s cultured media at 37°C. After 48 hours of incubation, the cells were treated with cell culture medium containing 10% Cell Counting Kit-8 (Dojindo) solution for 30 min. The cell viability was measured using a microplate reader (VersaMax, Molecular Devices Corp.) at a wavelength of 450 nm. See Fig. 7A for the results.

#### Cuproptosis-associated marker protein detection

For detection of cuproptosis-related proteins, 1 × 10^5^ of HT-29 cells were seeded onto microrobot-attached 35-mm glass-bottom confocal dishes. After 24 hours of incubation at 37°C, electric potential (Φ = 3 V) was applied to microrobot for 1 to 3 min. Following this treatment, the cells were incubated for an additional 4 hours. For fluorescent imaging, the cells were gently washed with Dulbecco’s phosphate-buffered saline (DPBS; WELGENE) and subsequently stained with SDHB antibody (Abcam, ab14714), fluorescein isothiocyanate (FITC)–conjugated LIAS antibody (SUNLONG, SL18247R-FITC), FITC-conjugated DLAT antibody (biorbyt, orb400442), and Alexa Fluor 647–conjugated adrenal ferredoxin (FDX1) antibody (Mybiosource, MBS9453137). For SDHB staining, an additional step involved incubation with FITC-conjugated anti-rabbit second antibody (Invitrogen, A32733) for 30 min at 4°C. Subsequently, all plates were washed with DPBS two times and fixed with 4% paraformaldehyde for 10 min. After another two DPBS washes, the nuclear of cells were stained with 4′,6-diamidino-2-phenylindole (DAPI) for 5 min in the dark. Cellular fluorescence imaging was performed using a Leica TCS SP8 laser-scanning confocal microscope (Leica Microsystems GmbH).

#### In vitro viability test of cancer cell monolayers

For the characterization of the anticancer therapeutic effect of M-cuproptosis, multiple experimental sets of M-cuproptosis treatment with varying Φ, *d*, treatment duration (*t*), electrode configuration, and cell type were conducted in parallel while the samples were continuously incubated at 37°C. Unless otherwise specified, the treatment condition of Φ = 3 V, *d* = 3 mm, *t* = 30 min, P2 configuration, and HT-29 was used. After the treatment, 3-hour incubation was followed for the resting stage of each monolayer sample, and the cancer cells were subsequently stained with the Live/Dead Cell Imaging Kit (Invitrogen, Thermo Fisher Scientific Inc.) based on the supplier’s specification. Fluorescent imaging of live cells (excitation of 488 nm and emission of 520 nm) and dead cells (excitation of 528 nm and emission of 617 nm) was performed using a fluorescent microscope (Nikon Eclipse Ti-E).

#### Cell monolayer viability data analysis

Fold viability of control and treated cancer cells was assessed by processing the overlaid composite fluorescent images of live and dead cells using an image-processing software (ImageJ). After loading, each image was converted into the binary format with a proper threshold level, followed by adjustable watershed segmentation. The automated counting of live or dead cells was sequentially performed only in the target area, defined by *d*. If necessary, the calibration process was included to minimize the counting error.

#### Tumor spheroid preparation

HT-29 cells (2 × 10^3^, 70 × 10^3^, and 200 × 10^3^) were seeded onto separate wells in ultralow attachment 96-well spheroid microplates (Gibco) and cultivated for 72 hours at 37°C in a humidified 5% CO_2_ atmosphere to grow tumor spheroids with diameters of ~0.3, 1.2, and 2.5 mm, respectively. The as-grown tumor spheroids were moved to 35-mm cell culture dishes where a PMMA well (diameter = 8 mm, height = 10 mm) enclosing the microrobot or Cu wires with configurations of P2, P4-2, P4-3, and P4-4 was installed, all immersed in 300 μl of DMEM solution.

#### In vitro viability test of tumor spheroids

The standard protocol, as described in detail above, was used for M-cuproptosis treatment. For the experiments with the microrobot, the FUS transducer probe was first installed at the bottom of the water tank (water volume ~ 1 liter; three separate setups for parallel experiments), and the sample dishes (including the tumor spheroid, the microrobot, and the PMMA well; all immersed in DMEM solution) were then immersed into the tank and positioned right at the focal point of FUS waves. Note that the samples must be tightly enclosed by a sealing film (Parafilm M). Once the sample was precisely positioned, the 500-kHz FUS signal with the optimal range of acoustic pressure (fig. S5) was applied to initiate M-cuproptosis. The treated tumor spheroids were incubated overnight at 37°C in a humidified 5% CO_2_ atmosphere. For live/dead viability assay, the treated tumor spheroids were stained with the two-color fluorescent cell viability assay solution (Invitrogen) by following the supplier’s specification. 3D fluorescent imaging of live cells (excitation of 488 nm and emission of 520 nm) and dead cells (excitation of 528 nm and emission of 617 nm) was performed using a fluorescent microscope (Nikon Eclipse Ti-E) with multilevel *z* axis offsetting. For tumor ATP assay, the treated tumor spheroids were moved into each well of a black/clear bottom 96-well plate (Corning), and luminescent spectroscopy was performed by a microplate reader (BioTek Synergy 2). The ATP luminescent data were calibrated by CellTiter-Glo 3D Cell Viability Assay (Promega), according to the supplier’s instruction.

#### Ex vivo porcine tissue preparation

The fresh porcine stomach, small intestine, and colon were acquired from a local food factory in Ulm, Germany. These organs were carefully emptied and lightly cleansed with water, ensuring the preservation of the mucus layer. Human GI tract phantom models, obtained from a medical phantom company (Trandomed 3D Medical Technology Co.), were digitally modified using SolidWorks 2020 (Dassault Group). The modified 3D phantom models were then produced using a Form 3D printer (Form 3, Formlabs Inc.) and Elastic 50A resin (Formlabs Inc.) (movie S3). The tumor analog used for demonstration (movie S5) was prepared using the porcine fat spheroid (2 to 3 mm in diameter) and attached to the colon surface using agarose gel (1% aqueous-solution). In the experiments, porcine tissues were precisely cut and affixed to the human phantoms. All tests and characterizations were performed on fresh tissues within 24 hours, stored at a temperature of 2°C in a refrigerator.

#### Ex vivo locomotion test

The ex vivo locomotion test under ultrasound imaging was conducted with the permanent magnet actuation system as shown in fig. S16E. The organs were merged in water for sufficient ultrasound imaging contrast, and the ultrasound probe was fixed in touch with the organ upper face. The permanent magnet actuation system was placed under the organ. The actuation system and the ultrasound probe were connected using a holder so that the imaging scope would follow the actuation region. The probe was connected to the imaging machine (Vevo 2100 from FUJFILM VisualSonics). The robot’s rolling locomotion was achieved by controlling the magnet’s rotation through the stepper motor and regulating the stepper motor’s motion via the *x*-*y* linear stages. The robot control in aid of endoscopic imaging system (Laserliner 082.256A Endoscope Probe, UMAREX GmbH & Co. KG) was also conducted with the permanent magnet actuation system as shown in fig. S16E. The endoscopic camera was inserted into the organ and was manually moved to focus on the robot. The permanent magnet actuation system was placed under the organ for robot control.

#### In vivo tumor xenograft model preparation

Eight-week-old male BALB/c nu/nu (Orient Bio) were bred under pathogen-free conditions at the Korea Institute of Science and Technology (KIST). For the preparation of xenograft mouse models, 1 × 10^6^ of HT-29 cells in 80 μl of PBS were subcutaneously inoculated into the left flank of the mice. All experiments involving live animals were conducted in accordance with the relevant laws and institutional guidelines of Institutional Animal Care and Use Committee (IACUC) in KIST, and IACUC approved the experiment (approval number 2022-021).

#### Ex vivo tumor penetration imaging and analysis

Tumors (volume = 150 to 200 mm^3^) were excised from the flanks of HT29 tumor-bearing mice and incubated ex vivo with either Cy5.5-labeled PS nanoparticles (CD Bioparticles; 10, 25, and 100 nm in diameter) or M-cuproptosis treatments. For nanoparticle-treated samples, tumors were incubated with PS particles (10 μg ml^−1^) for either 1 or 12 hours, followed by cryosectioning through the central region. For M-cuproptosis–treated samples, tumors were exposed to electrochemical dissolution of Cu^2+^ by the P4-4 configuration with *d* = 5 mm at Φ = 3 V for 1 or 12 hours. Penetration was assessed by direct fluorescence microscopy to visualize the distribution of Cy5.5 signal (for PS nanoparticles) or LIAS proteins within the tumor tissue. For spatial analysis of nanoparticle diffusion, the normalized fluorescence intensity was calculated by *I*(*x*)/*I*_max_, where *I*(*x*) is the fluorescence intensity at a distance *x* from the tumor periphery (*x* = 0) and *I*_max_ is the maximum intensity measured at the tumor periphery. For spatial analysis of cuproptosis, given the down-regulation of LIAS upon cuproptosis, cuproptosis expression was readjusted based on DAPI intensity, such that (relative cuproptosis expression) = *I*_DAPI_(*x*)/*I*_DAPI,max_ − *I*_LIAS_(*x*)/*I*_LIAS,max_.

#### In vivo tumor measurement

Once the HT-29 tumor volume reached 200 mm^3^, the tumor-covering skin was surgically removed, and the microrobot was anchored directly at the tumor site, ensuring complete coverage of the tumor tissue by suturing. Subsequently, a 24-hour incubation was implemented to enhance the adhesion of the microrobot to the tumor tissue, followed by 6-hour M-cuproptosis treatment. After the treatment phase, the skin was surgically reopened to detach the microrobot, and measurements of tumor volume and body weight changes were continued for 14 days. Tumor dimensions were measured with calipers, and tumor volume (mm^3^) was calculated as (width)^2^ × (length) × 0.5.

#### Tumor tissue analysis

For histological analyses, tumors were collected from mice after 24 hours of treatment, fixed in a 4% paraformaldehyde solution, embedded in paraffin, and sectioned to a thickness of 5 μm using a rotary microtome. The sections were then stained using a hematoxylin and eosin (H&E) staining kit (Abcam) and terminal deoxynucleotidyl transferase-mediated deoxyuridine triphosphate nick end labeling assay kit (Promega). To detect cuproptosis-related proteins, the deparaffinized and rehydrated sections were incubated with SDHB antibody, FITC-conjugated LIAS antibody, or Alexa Fluor 647–conjugated FDX1 antibody in PBS with 1% bovine serum albumin overnight at 4°C. For further SDHB fluorescence staining, slides were incubated with FITC-conjugated anti-rabbit second antibody for 1 hour at room temperature. Subsequently, all slides were washed with DPBS twice and incubated with DAPI for 10 min at room temperature. The H&E-stained tissues were observed using an optical microscope, and fluorescence images were visualized using a confocal laser scanning microscope.

#### In vivo biocompatibility test

The biocompatibility of the microrobot was assessed through blood and histological analyses in BALB/c mice. Microrobots with a reduced scale (0.5 to 1 mm in length) were prepared to match the mean diameter of the mouse gut (~0.58 mm) for their oral administration (Fig. 7, B and C). The microrobot’s surface was O_2_ plasma treated for 2 min with a power of 60 W (VITA 8, Femto Science), followed by 3-aminopropyltriethoxysilane (APTES) functionalization for 10 min using a 5 wt % APTES solution. The functionalized microrobot was subsequently stained with Flamma@675 NHS ester (65.2 μM; BioActs) for 2 hours. The mice received single or multiple oral administrations (three times with a 1-hour interval), and near-infrared fluorescence imaging was conducted using an in vivo imaging system (IVIS, Lumina Series III, PerkinElmer). In parallel, blood samples, GI organs (stomach, small intestine, colon), and the five major organs (heart, liver, spleen, lung, and kidney) of the treated mice (*n* = 6) were collected. For serum biochemical analysis, blood samples were centrifuged at 1200 rpm for 20 min, and supernatants were stored at 4°C. The whole blood and serum analyses were conducted by DKKOREA Co., Ltd. For the histological analysis, the collected organs were fixed in a 4% paraformaldehyde solution, embedded in paraffin, and sectioned to a thickness of 5 μm using a rotary microtome. Subsequently, the sectioned tissues were processed for deparaffinization and rehydration and then sequentially stained with H&E. After dehydration, the stained tissues were analyzed using an optical microscope.

## Supplementary Materials

### The PDF file includes:

Notes S1 to S7 Figs. S1 to S21 Tables S1 to S4 Legends for movies S1 to S6 References
