# Supplementary material for: Microrobotic copper-rich electrochemical interfacing for targeted cancer theranostics in the gut
Source: Sci Adv. 2026 Mar 13;12(11):eaeb5934. doi: 10.1126/sciadv.aeb5934 (PMC12985744; doi:10.1126/sciadv.aeb5934)
Supplement: Supplementary file 1 — Notes S1 to S7 Figs. S1 to S21 Tables S1 to S4 Legends for movies S1 to S6 References [file sciadv.aeb5934_sm.pdf]

Science Advances
AAAS

Supplementary Materials for

Microrobotic copper-rich electrochemical interfacing for targeted cancer theranostics in the gut

Junghwan Byun *et al.*

Corresponding author: Junghwan Byun, junghwan@kist.re.kr; Yoosoo Yang, yangys@skku.edu;
Metin Sitti, sitti@is.mpg.de

Sci. Adv. **12**, eaeb5934 (2026)
DOI: 10.1126/sciadv.aeb5934

The PDF file includes:

Notes S1 to S7
Figs. S1 to S21
Tables S1 to S4
Legends for movies S1 to S6
References

Other Supplementary Material for this manuscript includes the following:

Movies S1 to S6

Supplementary Note S1. Details in dynamics and mechanism of action of M-cuproptosis.

Comparison of M-cuproptosis with cuproptosis

Cuproptosis is the copper-induced cell death mechanism which is mediated by protein lipoylation during mitochondrial respiration cycles, uncovered in 2022 by Tsvetkov *et al.* (*40*). They found that the cuproptotic pathway is triggered by intracellular excessive Cu accumulation, which promotes the aggregation of mitochondrial lipoylated proteins, such as dihydrolipoamide *S*-acetyltransferase (DLAT), and in turn develops proteotoxic stress resulting in cell death. This copper-dependent toxicity was found to be distinct from all other known mechanisms of regulated cell death, including apoptosis, ferroptosis, pyroptosis, and necrosis (*40*). Transmembrane transport of Cu ions is usually regulated by CTR1 or SLC31A1 transporters, which can act as either an active ion pump (i.e., using metabolic energy, ATP) or a passive passage for facilitated diffusion. The majority of studies on cuproptosis have commonly used high-concentration copper ionophores (e.g., elesclomol) to accelerate the facilitated diffusion of Cu^{2+} across a cellular membrane. Leveraging ionophores aids in improving the efficiency of intracellular Cu^{2+} uptake; however, localizing their distribution to a specific site, such as a tumor, is hardly achievable via either oral or intravenous administration. We focused on another (but equivalent) aspect of their conclusion, implying that excess Cu^{2+} supplementation results in the same cellular effects as those induced by copper ionophores (*40*). Evidence of increase in transmembrane Cu^{2+} influx, induced solely by Cu^{2+}-rich solutions, was provided by previous experimental studies where 7-h-incuvation of diverse cancer cell lines in physiological fluids with the excess extracellular Cu^{2+} concentration ($[Cu^{2+}]_{out}$ = 600–1,400 μM) led to around 20-fold increase in the intracellular concentration, $[Cu^{2+}]_{in}$, which in turn triggered cuproptotic cell death within 24 h (*42*).

Microrobotic cuproptosis (M-cuproptosis), presented in this study, basically shares the same cell-killing mechanism with cuproptosis. In light of pharmacokinetic profiles required for scalable and effective cancer treatment, the key uniqueness differentiating our M-cuproptosis from other approaches is threefold: (i) locomotion-driven, direct tumor targeting and localization of bulk precursor (Cu) sources without premature release, (ii) wirelessly activated, in situ electrochemical dissolution of Cu^{2+} in close proximity of the target tumor micro-environment (TME) which induces a sharp elevation of $[Cu^{2+}]$ by a factor of $>10^4$ (~10–50 mM inside the Cu-CIC) (Fig. 1F), and (iii) microrobot-mediated exogenous electric fields (E-fields) that enhance ion kinetics for efficient, deep tumor penetration and saturation. These factors act in a mutually complementary manner, achieving unique pharmacokinetic profiles that feature both near-unity tumor-targeting efficiency and enhanced tumor penetration for GI cancers, with high spatiotemporal precision (Fig. 1, D and E). Further details on dynamics of M-cuproptosis will be discussed in separate subsections as below.

Mathematical model of electrochemical Cu^{2+} release

In our M-cuproptosis system, in situ electrochemical dissolution of bulk solid Cu sources leads to burst release of Cu^{2+} ions from each leg (anode) of the microrobot (note S2 for details on the reaction equation and onset potential). Colorimetric absorbance spectroscopy analysis shows that the characteristic curve of this Cu^{2+} production process, M(t), features a sharp increase in $[Cu^{2+}]$ at an early stage (< 30 min) and asymptotically approaches a saturating concentration after 120 min (Fig. 2B). We see that the main reaction equation of the Cu^{2+} release process at the anode

side, Cu(s) → Cu^{2+}(aq) + $2e^-$, shares the apparent similarity in its form with that of the enzyme-substrate binding reaction model (*77*), which is expressed as:

$$EnS \rightarrow En + P \quad \text{(S1)}$$

where *En*, *S*, *EnS*, and *P* denote enzyme, substrate, enzyme-substrate binding, and product, respectively. In addition, the electrochemical dissolution process of Cu can be assumed as a single substrate (Cu)-product (Cu^{2+}) system, and also has a limiting factor dependent on time that originates in faradaic loss as well as overpotential increase, induced by diffusion-limited ion saturation and/or oxide film formation at the Cu surface (see note S2). These implications also well meet the requirements of biochemical reaction models that follow Michaelis-Menten kinetics, consequently leading us to adopt the mathematical model of Michaelis-Menten kinetics to describe the Cu^{2+} production model, such that:

$$M(t) = \frac{\beta_1 t}{\beta_2 + t} \quad \text{(S2)}$$

where β_1 and β_2 are the fitting constants, and t is the reaction time. Note that concentration, c, is replaced with time, t, from the original model; therefore, M(t) in eq. S2, specifies the time-dependent quantity—thus, measured in µg or nmol— rather than rate of product formation. The differentiation of eq. S2 accordingly gives the rate of Cu^{2+} production as:

$$\dot{M}(t) = \frac{\beta_1 \beta_2}{(\beta_2 + t)^2}\,. \quad \text{(S3)}$$

To best relate $\dot{M}(t)$ with numerical studies, we had to convert its unit, nmol s^{-1}, into mM, implying that it is necessary to introduce an effective volume within which as-released Cu^{2+} ions are assumed to occupy without loss of generality. We considered that given the cylindrical model of Cu sources, as shown in fig. S6A, the time-dependent Cu^{2+} concentration function, $c(t)$ can be expressed as:

$$c(t) = \frac{\dot{M}(t)}{u_{x0} A_{yz}} = \frac{\dot{M}(t)}{\mu E_{x0} A_{yz}} \quad \text{(S4)}$$

where x is the direction normal to the surface of the Cu source, u_{x0} is the x-component velocity at (or in close proximity of) the Cu surface, A_{yz} is the effective area in yz plane that as-released Cu^{2+} ions occupy, E_{x0} is the x-component E-field at (or in close proximity of) the Cu surface, and μ is the ionic mobility. Here, the expression of μ can be obtained by the Einstein-Smoluchowski relation, which reads as:

$$\mu = \frac{ze}{6\pi\eta r_h}, \quad \text{(S5)}$$

where z is the valence, e the elementary charge, η the dynamic viscosity of the fluid, and r_h the hydrodynamic radius. Based on the common physiological condition ($\eta \approx 6.913 \times 10^{-4}$ Pa·s for water or PBS at T = 310 K) and Stokes radius for Cu^{2+} ($r_h = k_B T / 6\pi\eta D \approx 6.5\times10^{-11}$ m), a simple calculation yields $\mu \approx 3.78\times10^{-8}$ $m^2 \cdot V^{-1} \cdot s^{-1}$. Additionally, the numerical analysis of the E-field profile, as shown in fig. S8, provides with $E_{x0} \approx 10^4$ V m^{-1}. Given that Cu^{2+} ion transport is governed by the convective (i.e., electromigration) diffusion dynamics (*48, 79*), we calculated A_{yz} as a circular area defined by an effective radius, R_{yz} satisfying $A_{yz} = \pi R_{yz}^2$, which can be expressed as:

$$R_{yz} = R_{Cu} + L_{diff} \quad \text{(S6)}$$

where R_{Cu} is the radius of Cu wires (= 75 µm) and L_{diff} is the characteristic length of diffusion in yz plane, given by $(4\pi D t_0)^{1/2}$ (D: diffusion coefficient, t_0: time). Combining eqs. S3, S6 and related calculations into eq. S4, we obtain,

$$c(t) = \frac{\dot{M}(t)}{\pi\mu E_{x0}\left(R_{Cu}+\sqrt{4\pi D t_0}\right)^2} = \frac{\beta_1\beta_2}{\pi\mu E_{x0}\left(R_{Cu}+\sqrt{4\pi D t_0}\right)^2(\beta_2+t)^2}\,. \quad \text{(S7)}$$

It is noteworthy that t_0 is the only variable parameter in eq. S7 that we can control to make the best fitting between experiments and simulations. By introducing an adequate value of t_0, we finally obtained $c(t)$ functions at varying Φ: $c_2(t) = 294082.2/(t + 361.67)^2$, $c_3(t) = 587428.7/(t + 438.91)^2$, $c_4(t) = 668222.3/(t + 389.56)^2$ respectively for Φ = 2, 3, and 4 V, all of which were used for numerical studies as input boundary conditions (fig. S11).

Dynamics of interstitial Cu^{2+} transport and tumor penetration enhancement

Previous studies support that transport of molecules including ions within tumor interstitium is primarily diffusive due to high interstitial fluid pressure in tumors (*48, 78*). This diffusive nature allowed us to reason that the intra-tumoral permeation process of Cu^{2+}, in the form of Cu-CICs, can be interpreted as diffusive ion transport in a medium characterized by effective diffusivity (D_{eff}), without consideration of complex convective transport via microvasculatures. Under this assumption, the Nernst-Planck equation (Eq. 1), characterized by concentration (c), D_{eff}, and E ($=\nabla\Phi$), governs the kinetics of interstitial ion transport. To be specific, D_{eff} should be treated not as a constant for a homogeneous medium, but instead as a spatial function, $D_{eff}(x, y, z)$, to involve factors like tumor geometry, composition, and E-field profiles. Noting that D is closely related to r_h through the Stokes-Einstein relation, we established mechanistic models of size-dependent D in tumor tissues, such as extracellular matrix (ECM), and interstitial matrix (IM), in order to understand to what extent our approach could enhance the tumor penetration effect (Fig. 2G and note S4). The contribution of E-fields to interstitial ion kinetics and tumor penetration was explored by integrative data analyses of E-fields, Pe, and D_{eff} (Taylor-Aris dispersion approximation) (Fig. 3 and fig. S13) as well as specially designed in vitro experiments (fig. S10).

Based on these investigations, we confirmed that M-cuproptosis delivers strong therapeutic benefits in terms of tumor penetration and saturation for the following aspects: First, Cu^{2+} ion is extremely small from a perspective of hydrodynamic interaction. r_h of Cu^{2+} is calculated as ~0.065 nm whose effectiveness can be verified by the empirically obtained ionic radius ~0.073 nm (*79*); on the other hand, r_h of most existing anticancer (nano)medicines or agents fall within the range of 1–200 nm. Given $D \sim 1/r_h$, such a small r_h of Cu^{2+} effects on the significant enhancement of the ordinary (or free) diffusivity (D_0, diffusivity for water or PBS) by 2 to 3 orders of magnitude (Fig. 2G). Furthermore, the mechanistic models of D for spherical core-shell (IM-ECM) tumor structure, as described in fig. S13, suggest that the effective pore radius is estimated to be ~1–2,000 nm, implying that steric hindrance is significant only for sufficiently large molecules/particles (approximately with $r_h > 1$ nm). In other words, as of importance, Cu^{2+} ions make the tumor interstitial space feel like free physiological solution—that is, $D_{eff}/D_0 \approx 1$ (at Φ = 0 V) —whilst larger molecules are inescapably subjected to the effect of steric hindrance by a factor of 10^1 to 10^2, yielding $D_{eff}/D_0 \approx 0.01$ to 0.1 depending on r_h (Fig. 3B). Combining the gap in D_0, a factor of 10^2 to 10^3, into the steric hindrance effect allowed us to arithmetically calculate the effective benefit for tumor penetration, in terms of D_{eff}/D_0, as on the order of 10^3 to 10^5.

Second, the microrobotic exogenous E-field multiplies the kinetic energy of Cu^{2+} ions. fig. S13, B and C, shows that an application of Φ = 3 V leads to $Pe \approx 28.37$ in average throughout the entirety of the tumor region, implying that the target site of action is governed by the kinetics far beyond pure diffusion, which is conclusively electromigration (see also fig. S8 and note S3 for details on profiles and singular behaviors of E-fields). Numerical studies of moving EC90 fronts suggest that the electromigration-dominated Cu^{2+} kinetics enhances both performance and efficiency of tumor penetration compared to pure diffusion (Φ = 0 V), by at

least 100 times (fig. S13). To verify whether this theoretical and numerical estimation is valid, we designed a set of in vitro experiments in which a short-term treatment (duration, t_1 = 1, 3, 5 min) of M-cuproptosis was followed by 30 min of a resting stage or an additional exogenous E-field (P2 configuration, Φ = 3 V) (fig. S10). Note that for the sample setup, a pair of chemically inert Pt wires were placed right next to the Cu wires to apply the secondary exogenous E-field, meaning that there was no additional supply of Cu ions and relevant byproducts under the secondary E-field. The results demonstrated about 20-fold increase in effective area of Cu^{2+}-dependent cell death (highlighted by red with live/dead assays exhibiting R_{CIC} changed from 0.3 to 1.3 mm) for the samples subjected to the secondary exogenous E-field (fig. S10), the value of which could be considered equivalent to ~80 to 100-fold increase for 3D tumors. This clear evidence again confirms that the electromigration effect accounts for a substantial share of macroscopic Cu^{2+} transport.

Lastly, the microrobotic exogenous E-field exerts the potential well effect that keeps Cu^{2+} ions confined within the tumor area and thus facilitates their retention. In general, the small r_h of Cu^{2+} possesses an intrinsic trade-off: due to the relation $D \sim 1/r_h$, we can take advantage of D enhancement as discussed above, but this large D inversely acts as a critical drawback such that Cu^{2+} can move freely out of the target site of action. Our M-cuproptosis involves spatially coordinated electric multipole fields, as shown in fig. S8, which provide a facile solution to further localize and enhance the cuproptotic therapeutic efficacy (see note S3).

Dynamics of intracellular Cu^{2+} uptake

The pathway of transmembrane ion transport is generally threefold: transporter protein (CTR1)-mediated active transport, facilitated diffusion through carrier proteins or ion channels, and simple diffusion through mostly the lipid bilayer (LB) and/or carrier proteins and ion channels. Given the range of [Cu^{2+}] and rate of cell-ion interaction, we reasoned that the M-cuproptosis process is dominated by passive ion transport, such as facilitated diffusion and simple diffusion. The equation of ion influx describing this combination of facilitated and passive diffusion can be expressed by the linear summation of the Michaelis-Menten kinetics and the first-order rate diffusion (*44*), such that:

$$v = \frac{V_{max}\, c}{K_m + c} + kc \tag{S8}$$

where V_{max} is the limiting rate at saturating concentration, c (=[Cu^{2+}]), for a given concentration of carrier proteins or ion channels (unit: mM min^{-1}), K_m is the Michaelis constant (unit: mM), and k is the first-order rate constant for passive diffusion of Cu^{2+} through the plasma membrane (unit: min^{-1}). Given the experimentally obtained values of K_m ~4.7–6.5×10^{-3} mM, V_{max} ~0.01–0.02 mM min^{-1}, and k ~4–7×10^{-4} min^{-1} (monolayer cells) (*44*), eq. S8 says that for sufficiently high c, $v = V_{max}/(K_m/c+1) + kc \approx V_{max} + kc \approx kc$. This indicates that simple diffusion—the second term in eq. S8— dominates the majority of diffusion for the regime of [Cu^{2+}] > 0.02 mM (*44*), which also falls within the lowest range of [Cu^{2+}] in our M-cuproptosis experiments where, for example, EC90 (=0.6 mM) >> 0.02 mM.

Based on the above investigation, we explored thermodynamics and key factors determining this major pathway of intracellular Cu^{2+} uptake (i.e., simple diffusion) during the M-cuproptosis process. A primary impetus for simple diffusion-driven transmembrane ion transport is the electrochemical gradient across plasma membrane (*45, 80*), which is closely associated with the rapid formation and electromigration of Cu-CICs in the proximity of target cancer cells as described in Fig. 2A. This electrochemical gradient is determined by the combination of chemical gradient (i.e., concentration gradient across a membrane) and electrical gradient (i.e.,

charge difference across a membrane). For ionic species, including Cu^{2+}, the electrochemical potential, Ψ, is given by (*45, 80*):

$$\Psi = RT \ln \alpha c + zF\Phi, \quad \text{(S9)}$$

where *R* is the gas constant, *T* the absolute temperature, *α* the activity coefficient, *c* the concentration, *z* the valency, *F* the Faraday constant, and Φ the electric potential. Given the expression of Ψ with the assumption that *α* is independent of the concentration of any ion, the equation of the ionic flux across plasma membrane, *J*, at given constant *T* can be expressed as:

$$J = -\frac{D}{RT} c \frac{\partial \Psi}{\partial x}, \quad \text{(S10)}$$

where *D* is the diffusion coefficient of the ion through cell membrane and the direction *x* is normal to the membrane surface. Provided that the transport phenomena are limited to the transmembrane domain (membrane thickness: δ_m), eq. S10 can be reduced to:

$$J = -D\left(\frac{dc}{dx} + \frac{zF}{RT} c \frac{d\Phi}{dx}\right) \quad \text{(S11)}$$

$$= -D\frac{dc}{dx} - D\frac{zF}{RT} c \frac{\Phi_{\text{out}} - \Phi_{\text{in}}}{\delta} \quad \text{(S12)}$$

$$= -D\frac{dc}{dx} + P_m \frac{zFV_m}{RT} c, \quad \text{(S13)}$$

where Φ_{in} and Φ_{out} are the electric potential for the intracellular ($x = 0$) and extracellular ($x = \delta_m$) region, respectively, V_m ($\triangleq \Phi_{in} - \Phi_{out}$) is the membrane potential, and P_m ($\triangleq D/\delta_m$) is the membrane permeability. Note that the first term corresponds to Fick's law of diffusion, and the second term represents the flux fueled by electrophoresis with an electric field, *E*. The solution of eq. S11, also known as the Goldman-Hodgkin-Katz (GHK) flux equation, gives:

$$J = P_m \xi \frac{c_{in} - c_{out} e^{-\xi}}{1 - e^{-\xi}} \quad \text{(S14)}$$

where

$$\xi = \frac{zFV_m}{RT}. \quad \text{(S15)}$$

Note that the M-cuproptosis condition keeps $[Cu^{2+}]_{out}$ > EC90 = 0.6 mM, whereas $[Cu^{2+}]_{out}$ falls within the order of 10^{-10} to 10^{-13} mM. By substituting $z = 2$ for Cu^{2+}, V_m = -50 mV for cancer cells, and T = 310 K into eq. S15, we obtain $\xi \approx 42.25$, implying that for $[Cu^{2+}]_{out} >> [Cu^{2+}]_{in}$, eq. S14 can be reduced to:

$$J = P_m \xi \frac{e^{-\xi}}{e^{-\xi} - 1} c_{out}\,. \quad \text{(S16)}$$

The comparison with eq. S8 derives the analytical expression of *k* for the cell membrane as:

$$k = P_m \xi \frac{e^{-\xi}}{e^{-\xi} - 1}\,. \quad \text{(S17)}$$

To determine P_m ($=D/\delta_m$), we reasoned that ion transport by simple diffusion occurs through both LBs and ion channels, and calculated the effective *D* through plasma membrane as $D_m = f_{LB}D_{LB} + f_{ch}D_{ch}$ where f_{LB} and f_{ch} are the fractional coefficients of surface area coverage for LB and ion channels, respectively, satisfying $f_{LB} + f_{ch} = 1$. We could approximate f_{ch} ~0.01–0.1, based on the logical estimate of the density of carrier proteins (and/or ion channels) ~100–1,000 μm^{-2} with the cross-sectional area of a single protein ~10 nm × 10 nm = 10^{-4} μm^2. By introducing D_{LB} ~10^{-22} (to 10^{-19}) $m^2\ s^{-1}$ and D_{ch} ~10^{-14} $m^2\ s^{-1}$ for Cu^{2+} (*81*), we obtained $D_m \approx f_{ch}D_{ch}$ ~10^{-16} to 10^{-15} $m^2\ s^{-1}$, which gives P_m ~2×10^{-8} to 2×10^{-7} m s^{-1} with δ_m = 5 nm (*82*).

Given the Cu^{2+} production performance—$[Cu^{2+}]$ > 10 mM in average within the Cu-CIC at Φ = 3 V, as shown in Fig. 2E— and the propagation behavior of EC90 fronts (Fig. 2, D and F), *J* was estimated to be about -4.60×10^{-7} mol $m^{-2}\ s^{-1}$ with $[Cu^{2+}]_{out}$ = EC90, where the negative sign implies the inward flux, namely the intracellular uptake. Note that this calculation is quite reasonable for our system because V_m is nearly invariant under enhanced Cu^{2+} influx as well as

E_{robot} (*83*). For commonly used cancer cell lines having a mean diameter of ~20 μm (*84*), the rate of intracellular Cu^{2+} uptake by a single cell is calculated to be:

$$\upsilon = JA_{cell}N_A \approx 2.09 \times 10^{10} \text{ ions min}^{-1} \tag{S18}$$

where A_{cell} is the surface area of the cell membrane and N_A is the Avogadro constant (≈6.02×10^{23} mol^{-1}). To check whether this theoretical implication is valid, we calculated the value of k for the monolayer cells (HT-29) with a density (ρ_{cell}) utilized for in vitro experiments ~800 cells mm^{-2} such that,

$$k = P_m\xi\frac{e^{-\xi}}{e^{-\xi}-1} \times A_{cell} \times \rho_{cell} \approx 4.66 \times 10^{-5} \text{ min}^{-1} \tag{S19}$$

which was found to be an order lower than the experimental value (*44*). This error may arise from the estimation of effective diffusion coefficient (D_m). For numerical studies of reactive diffusion (Fig. 2, D and F, and figs. S11 to S13), an experimental value of k (= 4.7×10^{-4} min^{-1}) was used to deduce more valid results. Although this peak value must decay in an exponential fashion along with the increase in intracellular Cu^{2+} content, the characteristic profile of intracellular Cu^{2+} accumulation as shown in (*44*), which exhibits linear (first 30 min) and asymptotic steady increase up to 120 min, supports that the use of the first-order reactive diffusion model is reasonable to study M-cuproptosis. As an adequate comparison group, Saporito-Magrina et al. studied the effect of Cu overload on cancer cell death (i.e., cuproptosis); wherein they exposed several cancer cell lines to Cu^{2+}-rich solutions (0.6–2.4 mM) and analyzed the time-dependent cell viability and intracellular Cu^{2+} content (*41*). The results showed that the internal Cu^{2+} content gradually rose by ~10 and 20 folds after 2h and 7h of simple diffusion (in the absence of E-fields), respectively. These experimental data let us reason that our M-cuproptosis probably enhances intracellular Cu^{2+} accumulation (or concentration) by over two orders of magnitude within the duration of treatment.

Supplementary Note S2. Reaction equation and onset potential of electrochemical dissolution.

M-cuproptosis is initiated by the wireless electro-oxidation process of bulk Cu mounted on the microrobot which creates a Cu^{2+}-rich extracellular environment in situ. The theoretical value of electric potential (Φ) for this Cu^{2+} production process can be estimated by the Nernst equation, which reads at the anode as:

$$\Phi_{Cu^{2+}/\mathrm{Cu}} = E^0 + \frac{RT}{zF}\ln[Cu^{2+}] \quad \text{(S20)}$$

where E^0 is the standard reduction potential ($E_{Cu^{2+}/\mathrm{Cu}}{}^0 \approx 0.34$ V), R is the gas constant (8.31 J K^{-1} mol^{-1}), T is the temperature (in K), z is valence of Cu^{2+}, and F is the Faraday constant (96,487 C mol^{-1}). Despite the relatively low E^0, we observed that the experimentally obtained onset potential ($\Phi_{onset} \approx 1.4$ V) is somewhat higher than the theoretical value ($\Phi_{cal} \approx E^0 \approx 0.34$ V) (fig. S6). This gap is caused by the overpotential, η_{over}, which consists of an activation overpotential for the evolution of hydrogen ($\eta_a \approx 0.5$ V), an ohmic overpotential (η_{ohm}) due to the formation of oxide layers at the anode, and a concentration overpotential (η_c) due to the production of hydrogen (H_2) gas and the increase in Cu^{2+} concentration.

To further investigate the electrochemical reactions and the corresponding effect of η_{over}, we conducted a set of characterizations for the bulk Cu during and after the electro-oxidation process under the applied potential of 3 V. The result of in situ optical characterization suggests that gas bubbles formed at the cathode indicated hydrogen gas evolution whilst a large amount of Cu^{2+}, visible in bluish green, was produced and distributed gradually over time around the anode (Fig. 2C and fig. S6, C and D). It indicates that the primary reaction equations are outlined as:

$$2H_2O + 4e^- \rightarrow H_2(g) + 2OH^- \text{(at the cathode)} \quad \text{(S21)}$$

$$Cu \rightarrow Cu^{2+} + 2e^- \text{ (at the anode)} \quad \text{(S22)}$$

To examine the dynamic evolution of Cu oxidation, XPS analysis was performed for the post-treated, oxidized Cu surfaces (fig. S7, C to H). The XPS chemical status investigation of Cu 2p reveals that Cu oxide complex layers were quickly formed, as indicated by the presence of a CuO bond peak (Cu $2p_{3/2}$: 934.18 eV) after 1 min. Changes in the Cu 2p chemical status of the samples under longer treatment durations, as shown in fig. S7G, suggest that the chemical composition of the Cu oxide layers became more complex, supported by the coexistence of CuO bonds (Cu $2p_{1/2}$: 955.92 eV), Cu_2O bonds (Cu $2p_{1/2}$: 952.54 eV, $2p_{3/2}$: 932.85 eV) and $Cu(OH)_2$ bonds (Cu $2p_{3/2}$: 935.71 eV) in the oxide composite. The appearance of $CuCl_2$ bonds ($2p_{3/2}$: 198.57 eV) (fig. S7H) after 30 min of the electro-oxidation process indicates the formation of chloride products, which is in good agreement with the result of energy dispersive spectroscopy (EDS) mapping that contains Cl elements on the anode surface (fig. S7A). Based on the comprehensive analysis of XPS and EDS data, we confirmed that side reactions can be summarized as follows,

$$Cu + H_2O \rightarrow CuO + 2H^+ \quad \text{(S23)}$$

$$Cu + H_2O \rightarrow Cu_2O + 2H^+ \quad \text{(S24)}$$

$$Cu + 2H_2O \rightarrow Cu(OH)_2 + 2H^+ + 2e^- \quad \text{(S25)}$$

$$Cu + 2Cl^- \rightarrow CuCl_2 + 2e^- \quad \text{(S26)}$$

The reaction equations indicate that the evolution of the copper oxide/chloride layer formation gradually degrades the electron transfer for main reactions, consequently increasing η_{ohm}. Besides, the large amount of Cu^{2+} (namely, Cu^{2+} cloud) near the anode, produced mainly in the initial stage, counteracts subsequent release and diffusion of Cu^{2+}; this can be easily derived from the Fick's law of diffusion. As a result, the ion cloud is getting stagnant, which in turn

contributes to the increase in η_c. The combined effect is the positive shift of η_{over}, which ultimately decreases faradaic efficiency of Cu^{2+} production to a saturated value as depicted in Fig. 2B.

Supplementary Note S3. Singularity and potential well effect of E-fields.

Our microrobot transduces external focused ultrasound signals to the refined electric potential (Φ), at a level sufficient to trigger in situ electrochemical dissolution of bulk Cu sources in the TME. The situation is very similar to electric multipoles—spatially distributed point charges with opposite signs— separate by specific distances (*d*); the differences are the scale of distance (when calculating electric dipole moment, the distance is considered negligible, while it falls within the millimeter scale in our case), the number of negative poles (or ground, GND), and spatial configuration (that is, activation mode of the microrobot) (fig. S8). Given *d* is large enough in our system, we considered the characteristic E-field profile more likely as the E-field generated by a point charge (Q) rather than an electric dipole, such that

$$E(r) = \frac{Q}{4\pi\varepsilon}\frac{r}{|r|^3} \quad \text{(S27)}$$

where ε is the permittivity, and *r* is the distance vector from the origin of the point charge. Equation S27 provides an implication that the E-field, generated by the microrobot, has singularities—that is, $|E| \rightarrow \infty$ near the singular point which is supposed to be the boundaries of Cu sources— and shows rapid decaying behaviors ($\sim 1/r^2$ where $r = |r|$). Based on the numerical results as depicted in fig. S8, E and F, we found that the generated E-field (P2 configuration) does have not only a singular behavior—$|E| > 10^4$ V m^{-1} near the Cu source— but also a rapid decaying profile as *r* increases. The presence of the cathode (GND) breaks the symmetry of the point-charge-like E-field, rendering $|E|$ much higher in the tumor region than the outside. This symmetry breaking plays a vital role in directing Cu^{2+} migration towards the target tumor as well as keeping $Pe > 10$ in the tumor interstitial space (for $r_{tumor} = 1.45$ mm and $\Phi = 3$V). In the similar context, the multi-level vector-field analysis shows that the direction of the microrobotic electric multipole field is radially inward throughout the entirety of the tumor, producing the potential well effect (fig. S8). This provides the implication that the distribution of as-released Cu^{2+} ions is readily confined to the tumor area, and their movement is directed towards the center of the tumor.

Supplementary Note S4. Mechanistic models of the size-dependent molecular diffusivity in the tumor.

The transport of molecules of various sizes (e.g., nanomedicines) in tumor tissues is primarily diffusive in nature due to high interstitial fluid pressure in tumors (*48, 78*). This led us to employ the well-established theoretical model of the molecular diffusivity (D) in the neoplastic porous tissues so that we could characterize the effective diffusivity of Cu^{2+} in direct comparison with pharmaceutical nanoparticles based on the same criteria. For the sake of simplicity, the GI solid tumor was modeled as the spherical core-shell structure which consists of the extracellular matrix (ECM, characterized by the ECM volume fraction, ϕ_{ECM}) and the interstitial matrix (IM, characterized by the radius, r_{IM}) (fig. S13A). The Wittrup's dual-scale right cylindrical pore model (*47*)—which was modified from the pore and fiber-matrix model (*85*) to achieve better fitting curves and empirical relationships with a broad spectrum of experimental measurements of parameters such as D, permeability (P), available volume fraction in the tumor (ϵ), and plasma clearance (k_{clear})— was adopted to explore the effective diffusivity of IM. The key result of this two-pore model reads as,

$$\frac{D_{pore}}{D_0} = \begin{cases} (1 - 2.105\lambda + 2.0865\lambda^3 - 1.7068\lambda^5 + 0.72603\lambda^6)/(1 - 0.78587\lambda^5), & \lambda < 0.6 \\ (1-\lambda)^2[2-(1-\lambda)^2]/\kappa, & 0.6 \le \lambda < 1 \\ 1, & \lambda \ge 1 \end{cases} \tag{S28}$$

where D_0 is the molecular diffusivity of the molecule in solution, $\lambda = r_{\mathrm{h}}/r_{\mathrm{pore}}$, r_{h} is the Stokes-Einstein radius (namely, hydrodynamic radius)—expressed by $k_{\mathrm{B}}T/6\pi\eta D_0$ where k_{B} is the Boltzmann constant, T is temperature, and η is the dynamic viscosity— and κ is the hydrodynamic drag factor of a spherical particle moving within liquid-filled cylindrical pores, which can be obtained from numerical solutions (*86*). For the two different pore scales (small, $r_{\mathrm{pore},s}$, and large, $r_{\mathrm{pore},l}$), eq. S28 gives $D_{\mathrm{pore}} = D_0(D_{\mathrm{pore}}/D_0)$; and, given the fractional coefficients of diffusion through each pore size (f_s and f_l satisfying $f_s + f_l = 1$), we could finally obtain the effective diffusivity over the entire tumor space as $D_{\mathrm{eff}} = f_s D_{\mathrm{pore},s} + f_l D_{\mathrm{pore},l}$. Noting that the two-pore model of tumor IM with pore radii of 13.8 nm (= $r_{\mathrm{pore},s}$) and 1 µm (= $r_{\mathrm{pore},l}$) at a ratio of f_s:f_l = 9:1 exhibits the best description of experimental data sets [see table S2 with experimental data obtained from (*17, 47, 87, 91*)], we chose the same parametric condition and studied the size-dependent diffusivity in the IM (D_{IM}) as shown in Fig. 2G.

To obtain the diffusivity in the ECM, which is mainly composed of a network of collagens, we employed the modified Brinkman model in combination with the Carman-Kozeny model (*48*). The Brinkman model, modified to account for hydrodynamic interactions and the resulting steric hindrance of a collagen gel medium, describes that:

$$\frac{D_{eff}}{D_0} = \left[1 + \sqrt{\frac{r_h^2}{P}} + \frac{1}{9}\left(\frac{r_h^2}{P}\right)\right]^{-1}, \tag{S29}$$

where P is the permeability of the effective medium. The Carman-Kozeny model relates P with the pore size of the cylindrical fiber (r_{gel}), gel porosity (ϵ_{gel}), and the geometric factor (k_{gel}, also called the Kozeny factor) reflecting the random orientation of fibers in the 3D gel medium, such that:

$$P = \frac{\epsilon_{\mathrm{gel}} r_{\mathrm{gel}}^2}{4k_{\mathrm{gel}}}. \tag{S30}$$

Here, ϵ_{gel} is defined as $\epsilon_{\mathrm{gel}} = 1 - \phi_{\mathrm{gel}}$ where ϕ_{gel} is the volume fraction of the collagen gel matrix, and k_{gel} is described as k_{gel}

$$k_{\mathrm{gel}} = \frac{2k_{+} + k_{\parallel}}{3}, \tag{S31}$$

where k_+ is the geometric factor for cylinders parallel to the flow and $k_{||}$ is for cylinders at right angles to the flow, expressed by:

$$k_+ = \frac{2\epsilon_{\text{gel}}^3}{(1-\epsilon_{\text{gel}})\left[\ln\left(\frac{1}{1-\epsilon_{\text{gel}}}\right)-\frac{1-(1-\epsilon_{\text{gel}})^2}{1+(1-\epsilon_{\text{gel}})^2}\right]} \quad \text{(S32)}$$

$$k_{||} = \frac{2\epsilon_{\text{gel}}^3}{(1-\epsilon_{\text{gel}})\left[2\ln\left(\frac{1}{1-\epsilon_{\text{gel}}}\right)-3+4(1-\epsilon_{\text{gel}})-(1-\epsilon_{\text{gel}})^2\right]}\,. \quad \text{(S33)}$$

By combining eqs. S29 to S33 with $r_{\text{gel}} = 2$ μm and $\phi_{\text{gel}} = 0.378$ (*46*), we studied the size-dependent diffusivity in the ECM (D_{ECM}) as shown in Fig. 2G [see table S3 with experimental data obtained from (*13, 48, 88-90*)].

Supplementary Note S5. Theoretical analysis of tumor penetration and saturation for systemically administered nanoparticles.

Size-dependent pharmacokinetic parameters

A theoretical study of the size-dependent pharmacokinetic profiles of systemically administered nanoparticles (NPs) was performed to better evaluate the therapeutic benefit of our M-cuproptosis. The mechanistic compartmental model of antibody uptake suggests that the molecular size (hydrodynamic radius, r_h) of tumor-targeting agents is closely related to four parameters involved in the tumor uptake process: diffusivity (D), tumor vascular permeability (P_v), available volume fraction (ϵ), and plasma clearance factor (k_{clear}) (*47*). An extension of the dual-scale pore model that illustrates the relationship between D and r_h in the spherical core-shell tumor model, as described in note S4, formularizes P_v and ϵ as a function of r_h such that:

$$P_v = f_{cap,s}P_{v,pore,s} + f_{cap,l}P_{v,pore,l} \tag{S34}$$

$$\epsilon = V_i\left(f_s\varphi_{pore,s} + f_l\varphi_{pore,l}\right) \tag{S35}$$

where $P_{v,pore} = D_0(D_{pore}/D_0)\varphi$, $\varphi = (1 - \lambda)^2$ for $\lambda < 1$ and $\varphi = 0$ for $\lambda \geq 1$ with $\lambda = r_h/r_{pore}$, V_i is the interstitial fluid volume fraction (≈ 0.5), and $f_{cap,s}$ and $f_{cap,l}$ are the fractional coefficients of capillary pore areas per unit membrane thickness for small and large pores, respectively ($f_{cap,s} + f_{cap,l} = 1$). The previously established empirical model for systemic clearance was employed to draw the mathematical implication of k_{clear}, which can be expressed as (*44, 92, 93*):

$$k_{clear} = \frac{A}{V_{plasma}}\left\{\frac{Be^{-\alpha r_h}}{1-f(r_h)+Be^{-\alpha r_h}f(r_h)} + g(r_h)\right\} \tag{S36}$$

where A is the rate coefficient of renal fluid filtration, B is the equilibrium partition coefficient, α is the empirical fitting constant, $f(r_h)$ is the size-dependent geometric correction term, and $g(r_h)$ is the non-renal clearance term. Figure S14, D to G, shows the plots of the above four parameters as a function of r_h, particularly for the range of interest, 1 to 100 nm.

Shrinking core model (SCM) for tumor saturation analysis

Based on the theoretical analysis of molecular size dependence of pharmacokinetic parameters involved deeply in tumor uptake, we explored the kinetic behavior and the associated characteristic time scale of NP diffusion within the tumor. For the sake of simplicity, we made the following assumptions that are empirically valid (*50*): (i) the tumor is considered a sphere, characterized by a radius of r_{tumor} and an effective diffusion coefficient of D_{eff} [$\triangleq \phi_{ECM}D_{ECM} + (1 - \phi_{ECM})D_{IM}$]; (ii) the NP-based anticancer effect is determined solely by antibody-antigen kinetics; (iii) the plasma concentration of NPs is the same across the tumor surface, c_{surf}; and (iv) NP diffusion is much slower than the binding reaction. In this formalism, NP penetration into the tumor can be interpreted as the kinetics of the moving reaction front. This indicates that a simplified analytical theory of catalytic chemical reaction that produces moving fronts—termed, a shrinking core model (SCM) (*93*)— can be equivalently applied to describe the NP permeation kinetics, with a remarkable agreement with both numerical and experimental data (*50*) (see fig. S14, A to C). The SCM derives the following equation of the reaction front at a critical radius, r_c:

$$\frac{t}{t_{sat}} = 1 - 3\left\{\frac{r_c(t)}{r_{tumor}}\right\}^2 + 2\left\{\frac{r_c(t)}{r_{tumor}}\right\}^3 \tag{S37}$$

where

$$t_{sat} = \frac{r_{tumor}^2[Ag]_t}{6\epsilon D c_{surf}} \tag{S38}$$

and $r_c(t)$ is the radius outside which all antigen is complexed with NPs (antibody), $[Ag]_t$ is the antigen concentration per tumor volume, and ϵ is the tumor volume fraction accessible to NPs.

Equation S38 provides estimates of the characteristic time scale to saturate the tumor with a radius of r_{tumor} = 1.45 mm—adopted for optimal microrobot design and numerical studies considering the results of the parametric study of d, the diameter of the target site of action— by diffusion limited permeation of NPs with a broad spectrum of molecular sizes (Fig. 2I and fig. S14H). For calculation of t_{sat}, we used $[Ag]_t = 8.303 \times 10^{-5}$ mM—estimated from the reported experimental data sets exhibiting 10^5 to 10^6 binding sites cell^{-1} (*50*) together with a tumor cell density of 1×10^8 cells g^{-1} (*94*)— and $c_{surf} = 1 \times 10^{-3}$ mM—estimated from the maximum among the physiologically relevant values reported in previous literature (*47, 94–97*) which also matches the upper bound of the practical dose range (~10^{-4}–10^{-3} mM) expected to be maximally tolerated in the tumor microenvironment, calculated from the product of mean delivery efficiency of ~0.7%ID (*5*) and the maximum tolerated dose of widely-used clinical anticancer therapeutics, such as doxorubicin, 5-FU, cisplatin, cyclophosphamide, bleomycin, docetaxel, etoposide, gemcitabine, irinotecan, and vinorelbine (*98*)— in combination with size-dependent parameters, ϵ and D. Note that c_{surf} was held constant adopted to characterize t_{sat}, which is the condition clinically achievable by steady intravenous administration (*22, 50, 95*). Equation S37 says that the kinetics of the moving reaction front, r_c, is defined by the characteristic curve, $x = 1 - 3y^2 + 2y^3$ as shown in fig. S14C, as well as the size-dependent variable, t_{sat}. By selecting the clinically relevant r_h (= 10, 25, 50, and 100 nm), we investigated the profiles of NP tumor uptake (fig. S14I) and the corresponding fractional tumor saturation (fig. S14J). The results show that even without considering the effects of clearance—that is, c_{surf} is kept constant as a saturated input boundary concentration— the performance of tumor infiltration, driven by pure diffusion, is about two to four orders of magnitude lower than M-cuproptosis (fig. S14J).

<u>Clearance effect and tumor saturation performance</u>

To explore the effect of clearance on the tumor uptake profile, we introduced the single exponential plasma clearance term, k_{clear} as described in eq. S36, which modified the expression of c_{surf} as:

$$c_{surf} = c_{surf,0} e^{-k_{clear} t} \tag{S39}$$

where $c_{surf,0}$ is the initial plasma concentration of NPs at the tumor surface[47]. Substituting eq. S39 into eq. S37, we obtained the modified pharmaco-kinetic profiles, which reveal that the tumor uptake process is divided into two regimes, the diffusion-limited loading phase and the metabolism-limited retention phase, based on the transition time, t_{trans} (fig. S14K). If further considering other factors like TME structure (e.g., microvasculature), binding affinity (K_d), and infusion type (e.g., subsaturating bolus intravenous injection), we reasoned that t_{trans} stipulated in fig. S14K must be prolonged with regard to the time of peak tumor uptake, which is defined as (*47*):

$$t_{peak} = \frac{\ln\left(\frac{k_{clear}}{\Omega}\right)}{k_{clear} - \Omega} \tag{S40}$$

where

$$\Omega = \frac{2P_v r_{cap}}{\epsilon r_{Krogh}^2}\left(\frac{K_d}{[Ag]_t/\epsilon + K_d}\right), \tag{S41}$$

r_{cap} is the capillary radius (= 8 μm, adopted from ref. 44), and r_{Krogh} is the average radius of tumor tissues surrounding each blood vessel [= 75 μm, adopted from (*47*)]. The estimates of t_{peak} > 24 h for the relatively small tumor (r_{tumor} = 1.45 mm) with NPs of $r_h \geq 5$ nm indicate that conventional chemotherapeutics via systemic delivery pose an inherent limitation for saturating tumors of clinically relevant size (fig. S14L). In addition, clearance modulus (Γ) for the target

penetration depth, R (here, set to be r_{tumor})—which is defined as the ratio between the characteristic time scale for tumor saturation (t_{sat}) and clearance ($t_{clear} = 1/k_{clear}$), such that:

$$\Gamma = \frac{t_{sat}}{t_{clear}} = \frac{r_{tumor}^2 k_{clear}[Ag]_t}{\epsilon D(c_{surf}+K_d)} \quad \text{(S42)}$$

— exhibits $\Gamma = 10^1–10^3 >> 1$ (or equivalently $t_{sat} >> t_{clear}$) for NPs of r_h = 1–100 nm, meaning that NPs will clear far before reaching the core of the tumor (fig. S14M). In the similar context, we could estimate the approximate penetration depth, R ~100–500 µm, by applying the condition $\Gamma = 1$ to eq. S42. This result confirmed that NPs with clinically and physiologically relevant sizes are effective only in treating tumors (or tumor cell aggregates) of r_{tumor} < 500 µm through the diffusion-limited complete saturation. Probably, this is why the majority of existing in vitro testing of tumor-targeting nanomedicines seemingly worked well for tumor spheroids of size ~500–1,000 µm, whereas there exists an intractable obstacle to their clinical translation.

Supplementary Note S6. Comparison of figure of merits of M-cuproptosis with existing E-field-assisted locoregional cancer therapies (table S4).

The E-field has been widely adopted as an alternative therapeutic modality for cancer due to its close relevance to various physico-biochemical functions. For example, an application of E-fields with an adequate range of frequency and intensity to biological organs, tissues, and/or fluids can lead to heat generation, electrochemical reaction (e.g., electrolysis), electro-biochemical reaction (e.g., disruption of plasma membrane or mitochondrion), and electro-physiochemical process (e.g., electro-osmosis), all of which could underlie the cell-killing mechanism. Here, we introduce four major types of E-field-assisted locoregional cancer therapy—radiofrequency (RF) or microwave (MW) electrothermal ablation (*51, 99–101*), electrochemical treatment (EChT) (*52, 102, 103*), irreversible electroporation (IRE) (*53, 104*), and tumor-treating fields (TTFields) (*54, 105*)— and compare them with M-cuproptosis to evaluate the effectiveness of our approach. Specifically, we considered factors involved in therapeutic effectiveness, safety, and long-term sustainability, such as cancer-killing mechanism, invasiveness, localization, local power density, duration, and temperature increase (table S4).

The key uniqueness of M-cuproptosis lies in the cancer-killing mechanism that relies on the Cu^{2+}-dependent cell death pathway, termed "cuproptosis". M-cuproptosis uses the untethered, mobile microrobot capable of producing Cu^{2+} ions and precisely controlling their concentration, distribution, migration, and the resulting pharmacokinetic profile in spatiotemporal domain. This indicates that the way of employing E-fields is fairly different from others; in M-cuproptosis, E-fields do not interact directly with cancer cells but, instead, regulate pharmacokinetics of the anticancer agent, Cu^{2+}. Therefore, the underlying principle of M-cuproptosis presents an invaluable potential for therapeutic stratagems that require local dose escalation and/or local pharmacokinetic modulation of therapeutic agents, whilst others are less relevant. Further, as evidenced by indicators such as locomotion-driven, non-invasive tumor targeting and anchoring, and very low local power density (fig. S6, H and I), M-cuproptosis shows its comparative advantages in precise localization, safety, and long-term sustainability although currently limited to GI tumors.

Supplementary Note S7. Kinetic model of magnetically-guided locomotion and anchoring

The kinetic model of the magnetically-guided robot involves an integrated approach to describe both the bending stiffness of the leg and its dynamic behavior under external magnetic actuation. This section summarizes the key aspects of the model, including the effective bending stiffness, locomotion dynamics, deflection characteristics, and anchoring mechanism.

Effective bending stiffness

Our cross-shaped magnetic robot is simplified as a magnetic beam as it is axis-symmetric about its central axis perpendicular to its body plane. As illustrated in fig. S4C, the robot leg is modeled as a composite beam with a soft elastomer layer and a thin Cu layer and the neutral plane is assumed as the mid-layer of the soft elastomer since the Cu layer thickness is negligible compared to the soft elastomer layer. The effective bending stiffness (K_{eff}) of the composite beam is calculated as:

$$K_{\text{eff}} = E_{\text{eff}} I_{\text{eff}} = \frac{E_{\text{s}} A_{\text{s}} + E_{\text{Cu}} A_{\text{Cu}}}{A_{\text{s}} + A_{\text{Cu}}} \cdot (I_{\text{s}} + I_{\text{Cu}}), \quad \text{(S43)}$$

where A_s and A_{Cu} are the areas of the cross-sectional shapes of soft magnetic elastomer and Cu layers, respectively. E_s and E_C are the Young's moduli, and I_s and I_{Cu} are the moment of inertias of soft magnetic composite and copper layers, respectively. The theoretical value of K_{eff} of the robot leg with and without the Cu layer is plotted as functions of the thickness of the soft magnetic layer, t_s, in fig. S4E.

Dynamics of the robot leg

The deflection of robot legs is governed by the Euler-Bernoulli beam theory under magnetic actuation (*34*):

$$\frac{d^2}{dx^2}\left[K_{\text{eff}} \frac{d^2 w}{dx^2}\right] = M(x) \quad \text{(S44)}$$

where $w(x)$ is the deflection of the beam at position x, and $M(x)$ is the magnetic moment induced by the external field. Boundary conditions depend on the beam's clamping or free edges, and numerical simulations are performed to solve the fourth-order differential equation. The beam dynamics under varying magnetic fields are experimentally characterized using customized setups (fig. S4, F and G). Helmholtz coils are used to generate a controlled magnetic field (B) and the leg's beam force, or recovery force, $F_{\text{r\_}B}$ is measured using a high-precision force test rig (*34*). The deflection $w(x)$ is measured using digital cameras (Blackfly S USB3, FLIR Systems).

Locomotion dynamics

The robot body's magnetization profile $\mathbf{M}(s)$ along its length direction (magnitude profile: $\mathbf{M}(s)$, phase profile: $\phi(s)$, s∈[0, L], where L is the leg length) is as shown in fig. S4A. When subjected to an external magnetic field $\mathbf{B}(t)$, the robot body deforms into a specific shape parameterized with the deflection angle $\theta(s)$,

$$\mathbf{n}_{\text{z}} \cdot A_{\text{s}}\big(\mathbf{R}[\theta(s)]\mathbf{M}(s)\big) \times \mathbf{B}(t) = -K_{\text{eff}} \frac{\partial^2 \theta(s)}{\partial s^2} \quad \text{(S45)}$$

where $\mathbf{R}$ denotes the rotation matrix, and $\mathbf{n}_z$ is the unit vector along the z axis. As the external magnetic field $\mathbf{B}(t)$ rotates, the deformed robot body also experiences a non-zero net magnetic torque, $\boldsymbol{\tau}_{\text{net}}$,

$$\boldsymbol{\tau}_{\text{net}} = \mathbf{M}_{\text{net}} \times \mathbf{B}(t) \quad \text{(S46)}$$

Where $\mathbf{M}_{\text{net}}$ is the net magnetic moment given by:

$$\mathbf{M}_{\text{net}} = \int_0^L \mathbf{R}[\theta(s)]\mathbf{M}(s)\, A_{\text{s}} \text{d}s. \quad \text{(S47)}$$

This non-zero net magnetic torque $\boldsymbol{\tau}_{net}$ controls the rolling motion of the robot body by varying the rotation direction of $\mathbf{B}(t)$.

<u>Anchoring</u>

The microrobot is attached to the target tumor, with their leg ends subject to 'fixed-fixed' boundary conditions that both ends adhere to the substrate so that they cannot move, as shown in fig. S16C. Under these conditions, the leg's beam force, F_{r_B}, due to the external magnetic field of B mT, balances the adhesion force, $F_{adhesion}$, between the adhesive patch and the tissue surface. The force equilibrium condition is described as:

$$F_{adhesion} = F_{r\_B} \tag{S48}$$

The beam force F_{r_B} as a function of the magnitude of the external magnetic field was experimentally characterized using a customized force measurement set-up (fig. S4G, inset). The set-up comprises a high-resolution load cell (GSO-25, Transducer Techniques) with a resolution of 0.01 mN, mounted on a vertical, high-precision piezo motion stage (LPS-65 2″, Physik Instrumente). One end of the robot leg was bonded onto the substrate and the other end was bonded to a 3D printed cubic tip (Clear V4, Formlabs Inc.) that was connected to the load cell. The tip approached and bent the robot until the two ends' distance was 3 mm. The external magnetic field was applied perpendicular to the robot surface via a cube magnet (20 mm × 20 mm × 20 mm, MagnetMax) and its magnitude was characterized by the gauss meter (GM07, Hirst Magnetics). To ensure firmly anchoring, the bioadhesive patch needs to provide high enough adhesion forces. As experimentally characterized in Fig. 5D and fig. S17, the experimental value of $F_{adhesion}$ is designed to exceed the beam force.

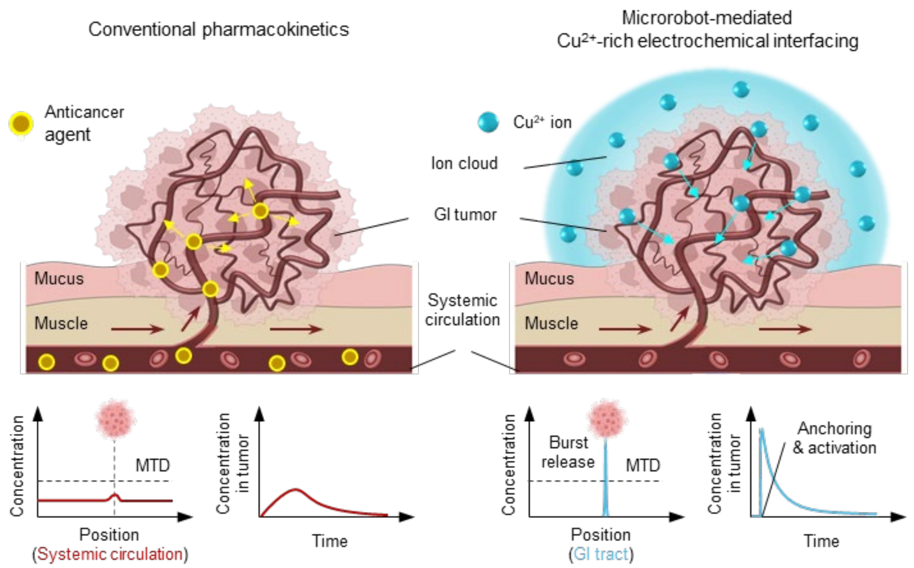

Fig. S1. Comparison between conventional pharmacokinetics and microrobotic Cu^{2+}-rich electrochemical interfacing for targeted cancer therapy.

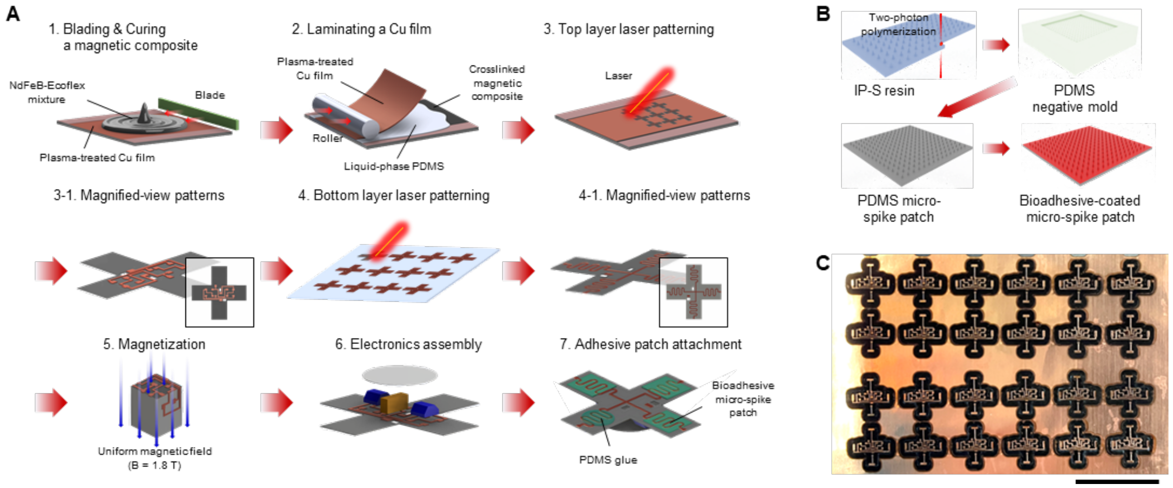

Fig. S2. Fabrication process of the microrobot. (**A**) Fabrication process of the main body of the microrobot with embedded electronics. (**B**) Two-photon polymerization-based microfabrication process for bioadhesive pads. (**C**) Representative photograph of mass-manufactured microrobots with electrical layouts. Scale bar, 1 cm.

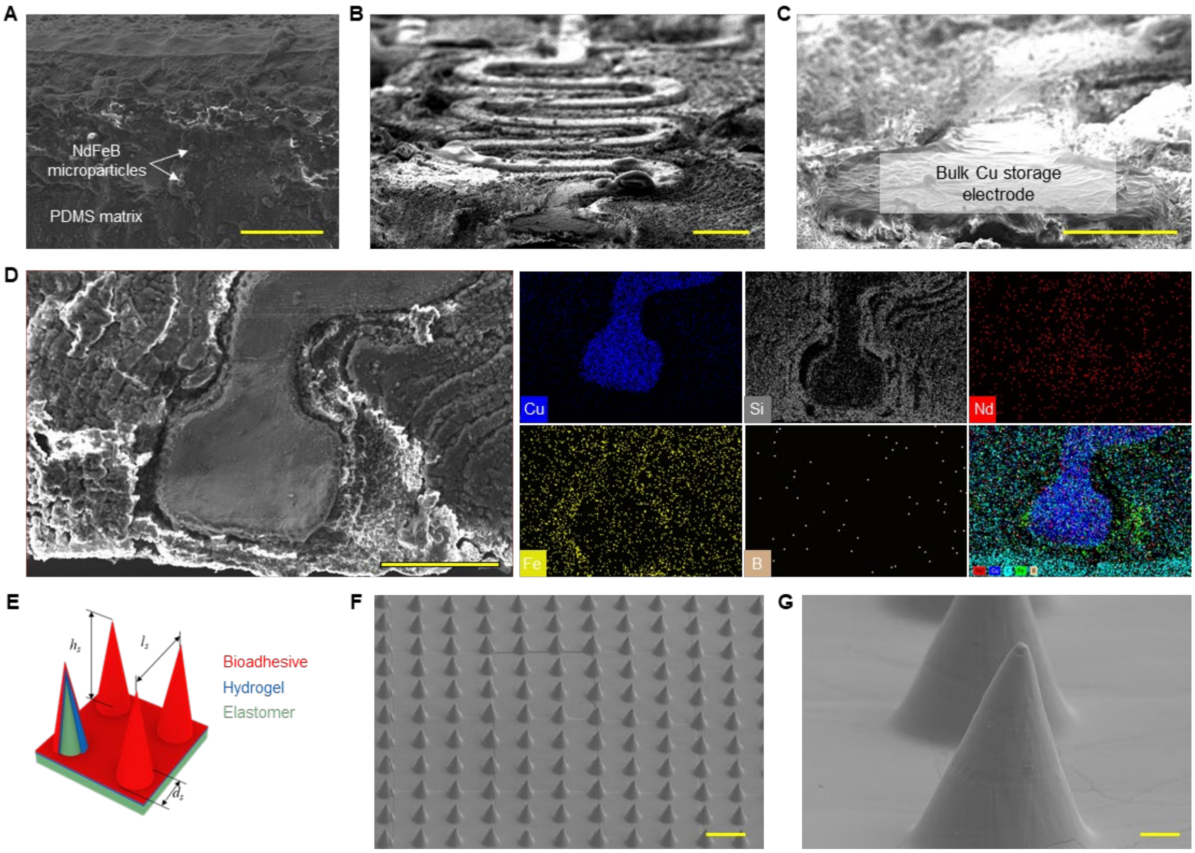

Fig. S3. Microrobot components. (**A**) Cross-sectional scanning electron microscope (SEM) image of the main body consisting of PDMS and NdFeB. (**B** and **C**) SEM image of bulk Cu storage electrodes with a thickness of ~10 µm. (**D**) SEM-EDS (energy dispersive X-ray spectroscopy) analysis of the microrobot's main components showing elemental mapping of Cu, Si, Nd, Fe, and B. (**E** to **G**) Schematic illustration (E) and the corresponding SEM images (F, G) of bioadhesive micro-spike patterns. Scale bars are 100 µm (A, B, D, F), 50 µm (E), 10 µm (G).

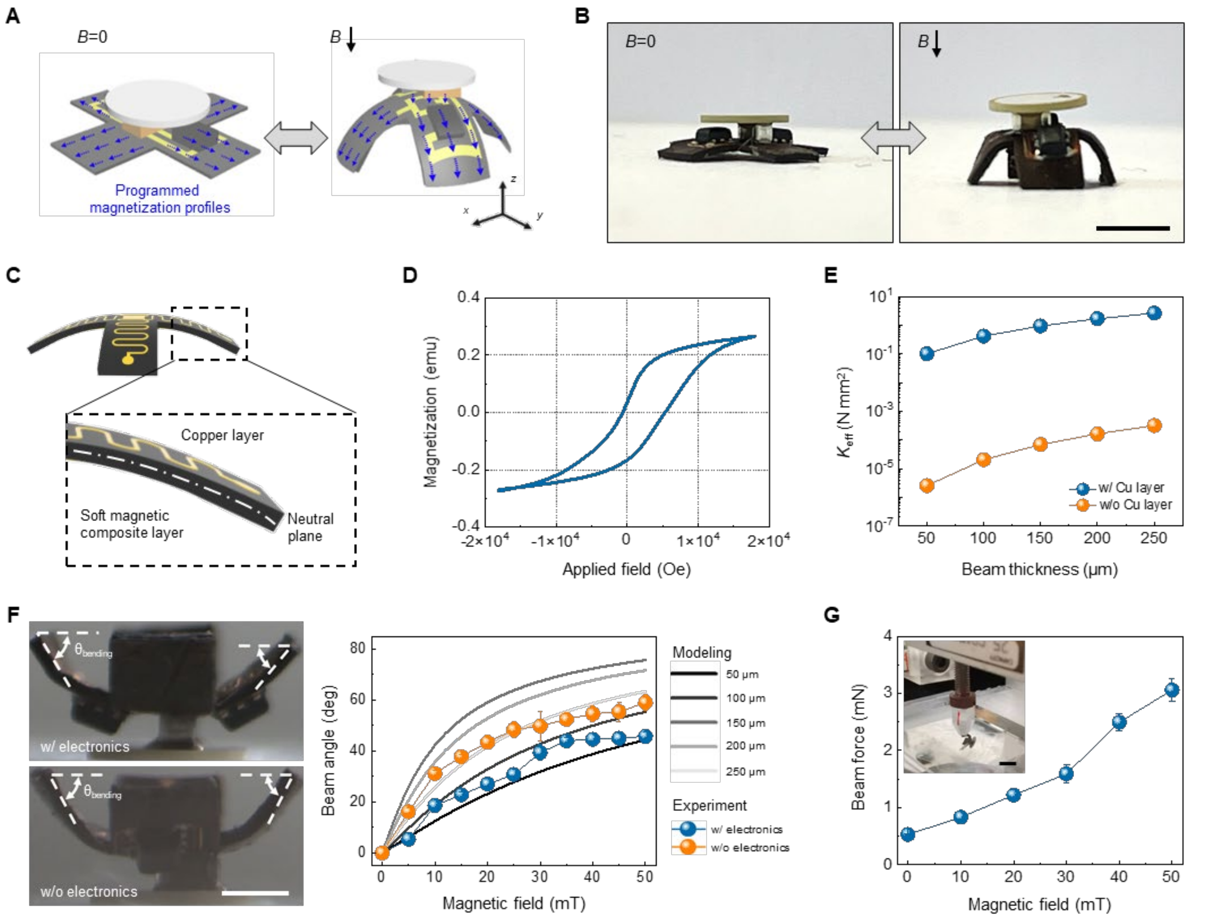

Fig. S4. Modeling and characterization of magnetic actuation. (**A** and **B**) Schematic illustration (A) and the corresponding photograph (B) of the microrobot with programmed magnetization. The radial magnetization profile along each robot leg was chosen to allow the pop-up and rolling modes of locomotion. (**C**) Schematic drawing of the main body that is considered a soft magnetic composite beam consisting of Cu serpentine traces (thickness, 8–25 μm) and a soft magnetic composite (thickness, 150 μm). (**D**) The magnetic hysteresis loop of the microrobot (PDMS/NdFeB) measured by a vibrating-sample magnetometer (VSM). (**E**) Theoretical estimation of effective bending stiffness (K_{eff}) of the beam, shown in C, as a function of thickness. (**F** and **G**) Beam angle (F) and bending force, or recovery force (F_{r_B}) (G) of the beam under the external uniform magnetic field (*B* mT). The Euler-Bernoulli beam theory provides an optimal range of beam thickness (150–200 μm) to maximize the beam angle, which aids in agile and efficient rolling motion. See note S7 for details on the kinetic model and dynamics of the microrobot. All data are means ± SD. Scale bars are 2 mm (B, G) and 1 mm (F).

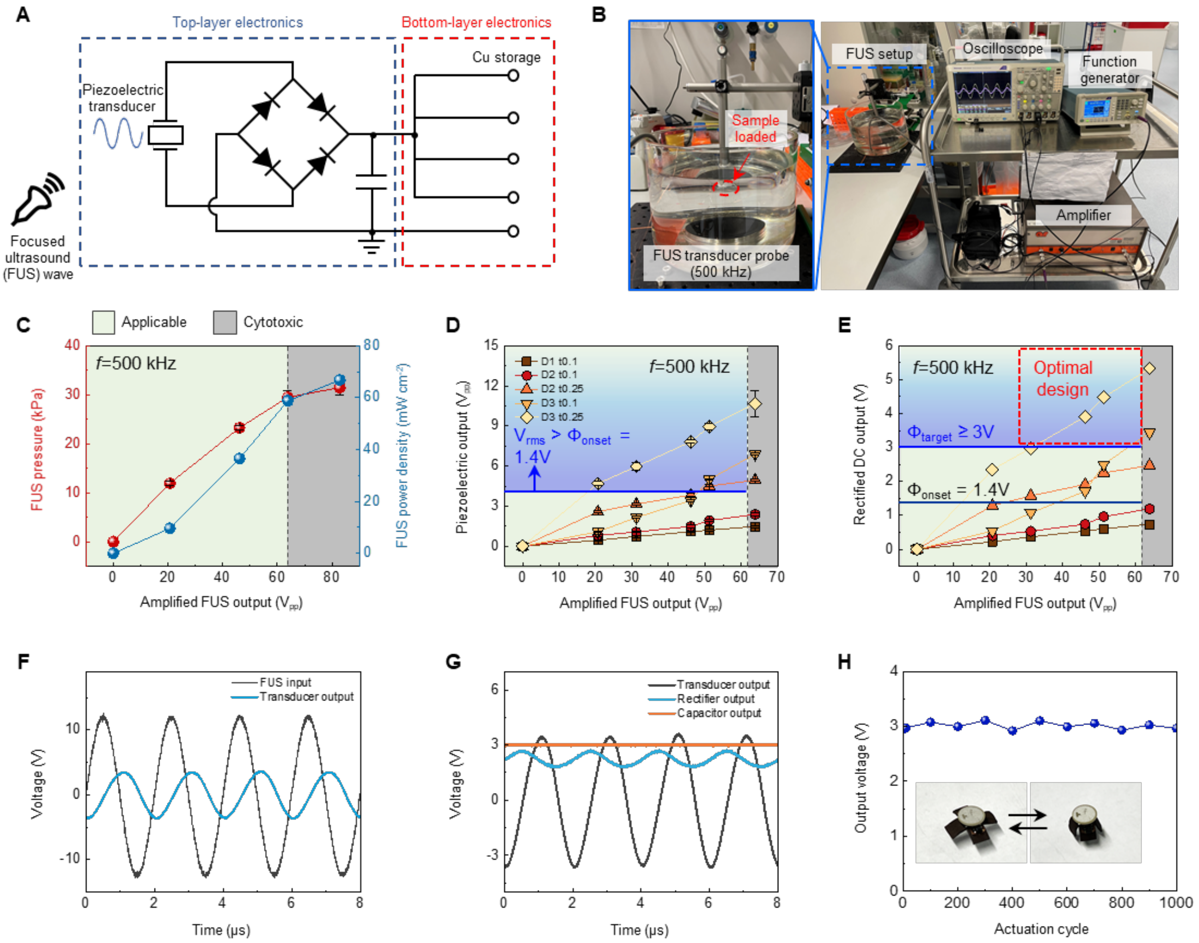

Fig. S5. Embedded electronics. (**A**) Schematic diagram of embedded electronics consisting of a piezoelectric transducer, four power diodes, a smoothing capacitor, and output Cu storage electrodes. The function is the same as the full wave rectifier. (**B**) Experimental setup for focused ultrasound system (FUS) and piezoelectric transduction characterization. (**C**) Acoustic pressure and power density characterization of the 500-kHz FUS system used in the study. Our observation suggests that the upper bound of the applicable FUS pressure, above which induces negative effects by itself such as detachment, weakening of intercellular cohesion, and cytotoxicity, is around 30 kPa that is equivalent to the power density of ~60 mW cm^{-2}, highlighted in light green. (**D** and **E**) Piezoelectric output (D) and on-board rectification performance (E) of piezoelectric transducers with different geometries (diameter, *D*, thickness, *t*, with units in mm). By measuring the peak-to-peak voltage (V_{pp}), we determined the feasible dimension of the piezoelectric transducer that falls within the target performance regime satisfying $V_{rms} = V_{pp}/2\sqrt{2} > \Phi_{onset} = 1.4$ V. (**F** to **H**) Electrical characterizations of on-board piezoelectric transduction (F), rectification and signal smoothing that lead to stable Φ_{target} (G), and reliability under repetitive magnetic actuation up to 1,000 cycles (H).

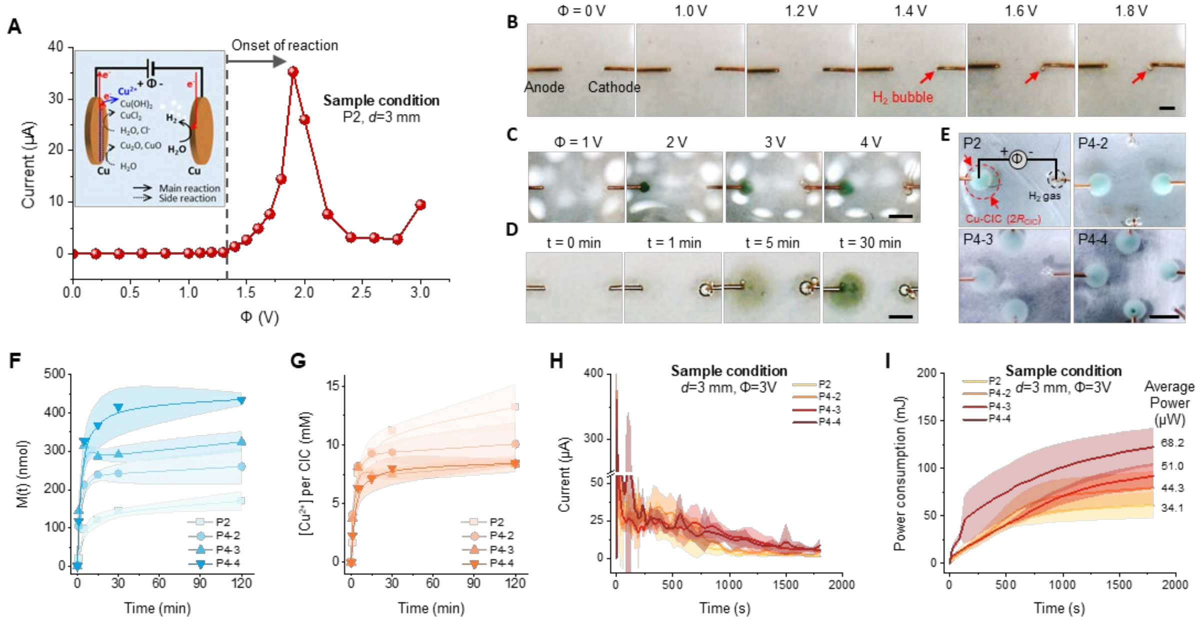

Fig. S6. In situ electrochemical dissolution of bulk solid-state Cu in the physiological fluid. (**A** and **B**) Experimental characterization of the onset of the electrochemical dissolution process of solid-state Cu (wires with a diameter of 150 μm) using the P2 configuration with $d = 3$ mm (A) and the corresponding sequential image frames as a function of Φ (B). The inset image in (A) shows the schematic of the main and side reactions of the process. (**C** and **D**) Sequential image frames of the Cu^{2+} release processes with increasing Φ for 30 min (C) and duration time at Φ = 3 V (D). (**E**) Optical images of in situ Cu electro-oxidation-driven CIC formation for various Cu source configurations (P2, P4-2, P4-3, and P4-4). (**F** and **G**) Total Cu^{2+} quantity (F) and concentration profiles in a single Cu-CIC (G) for different Cu source configurations. (**H** and **I**) Current level (H) and power consumption (I) characterization for various electrode configurations. All data are means ± SD. All scale bars indicate 1 mm.

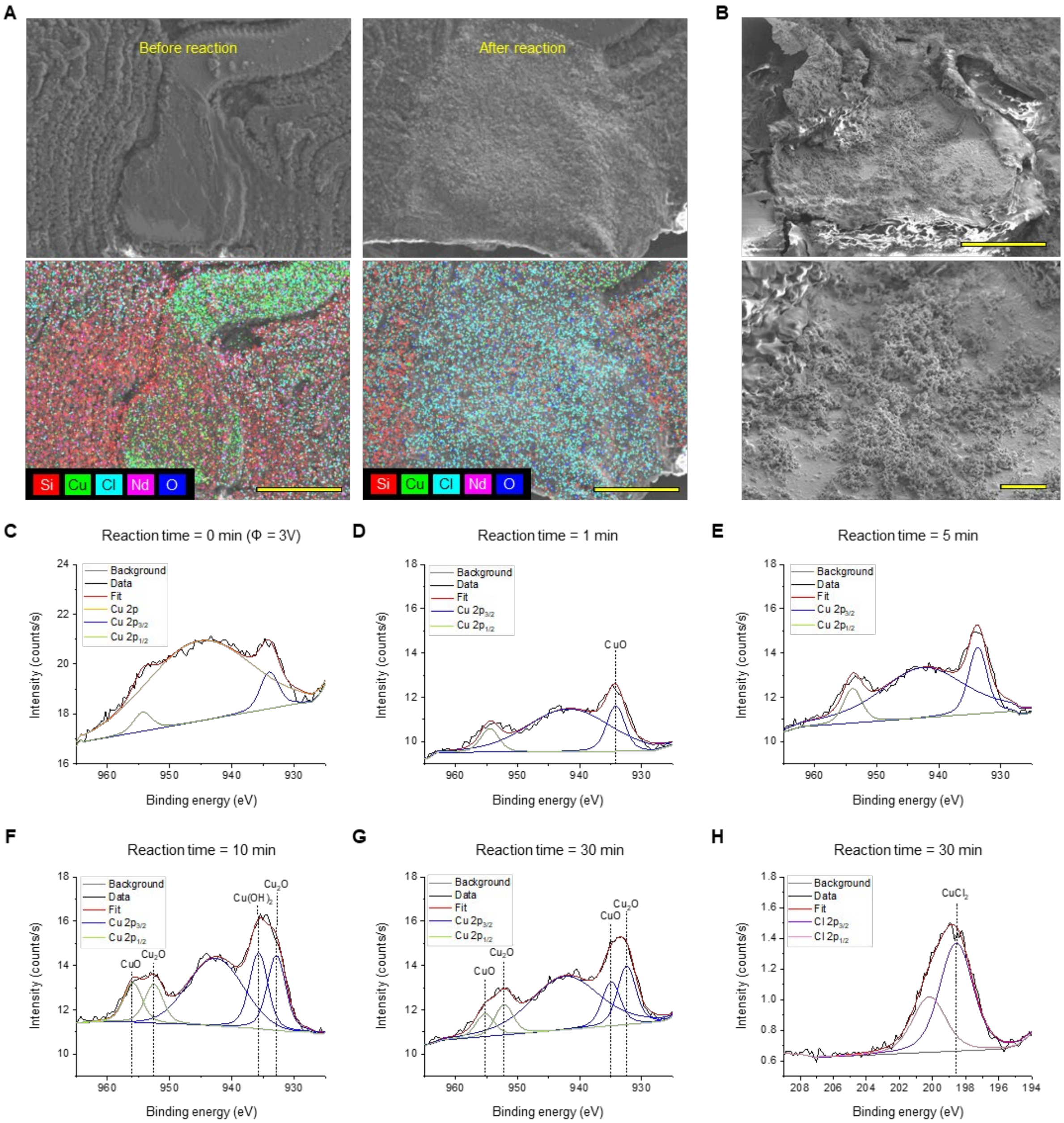

Fig. S7. Morphology and chemical composition of electrochemically oxidized Cu. (**A**) Overlaid SEM (top) and EDS (bottom) images of the Cu storage electrode integrated onto the soft magnetic composite (NdFeB/ecoflex) before and after the 30-min electro-oxidation process. Changes in chemical composition, such as the increase in Cl and O elements, verify the side reactions that form byproducts as shown in the inset of fig. S6A. (**B**) SEM images of the Cu storage electrode after the 2-h electrochemical reaction. (**C** to **H**) XPS results of the Cu storage electrode after 0, 1, 5, 10, and 30 min of electrochemical dissolution. The shifts and splitting in the Cu 2p orbital peaks correspond to the higher oxidation states (C to G), and while those in the Cl 2p orbital peaks represent the formation of $CuCl_2$ byproducts (H). Scale bars are 100 μm for (A), 50 μm (top) and 10 μm (bottom) for (B).

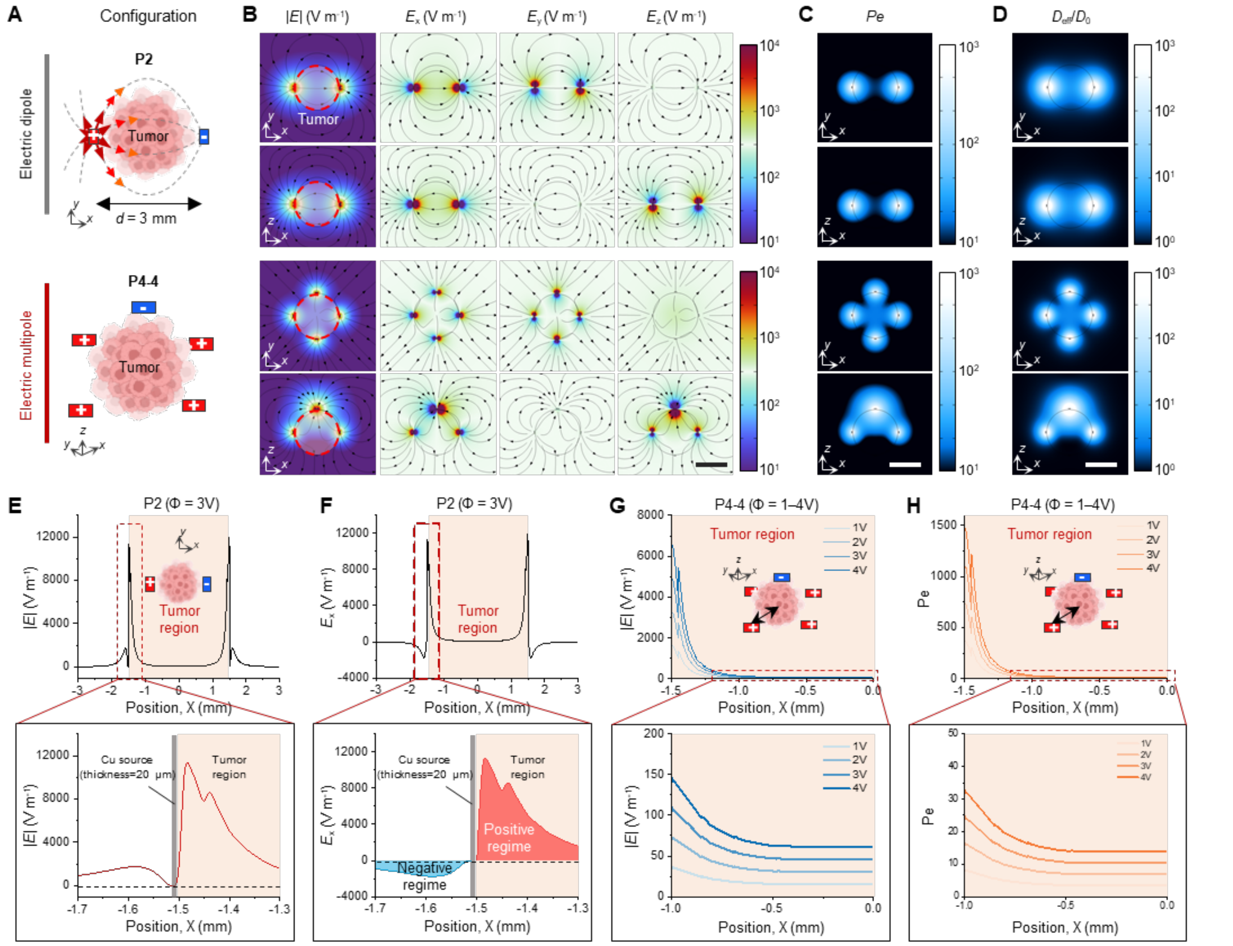

Fig. S8. 3D electrical interface generated by microrobotic electric multipoles (Φ = 3 V). (**A**) Schematic illustration of electric dipole (P2) and multipole (P4-4) separated by a distance of d (= 3 mm), encircling the target tumor (r_{tumor} = 1.45 mm). The effect of point charges with opposite signs was applied by a uniform electric potential (Φ) between each source. (**B** to **D**) Numerical simulation results of electric fields (E_x, E_y, E_z, $|E|$) (B), Pe (C), and normalized effective diffusivity, D_{eff}/D_0, (D) for each configuration at Φ = 3 V. (**E** and **F**) Typical singular profiles in close proximity of the point-like electric sources in the P2 configuration (d = 3 mm). The sharp valley in the $|E|$ profile, which is radially outward from each source, as shown in (B), and its exponential decaying behavior as a function of distance verify the existence of the E-field singularity (E). The asymmetry in E_x with sign reversal along the x-axis, which arises from the insulation boundary condition for the Cu source, supports the potential well effect (F) (see note S3 for details). (**G** and **H**) Typical singular profiles of E-fields, $|E|$, in the P4-4 configuration with increasing Φ (G), and the corresponding Pe profiles (H). Note that $Pe > 10$ for the entire tumor region on the x-axis at Φ ≥ 3V, implying that electromigration dominates the Cu^{2+} transport mechanism. All scale bars indicate 2 mm.

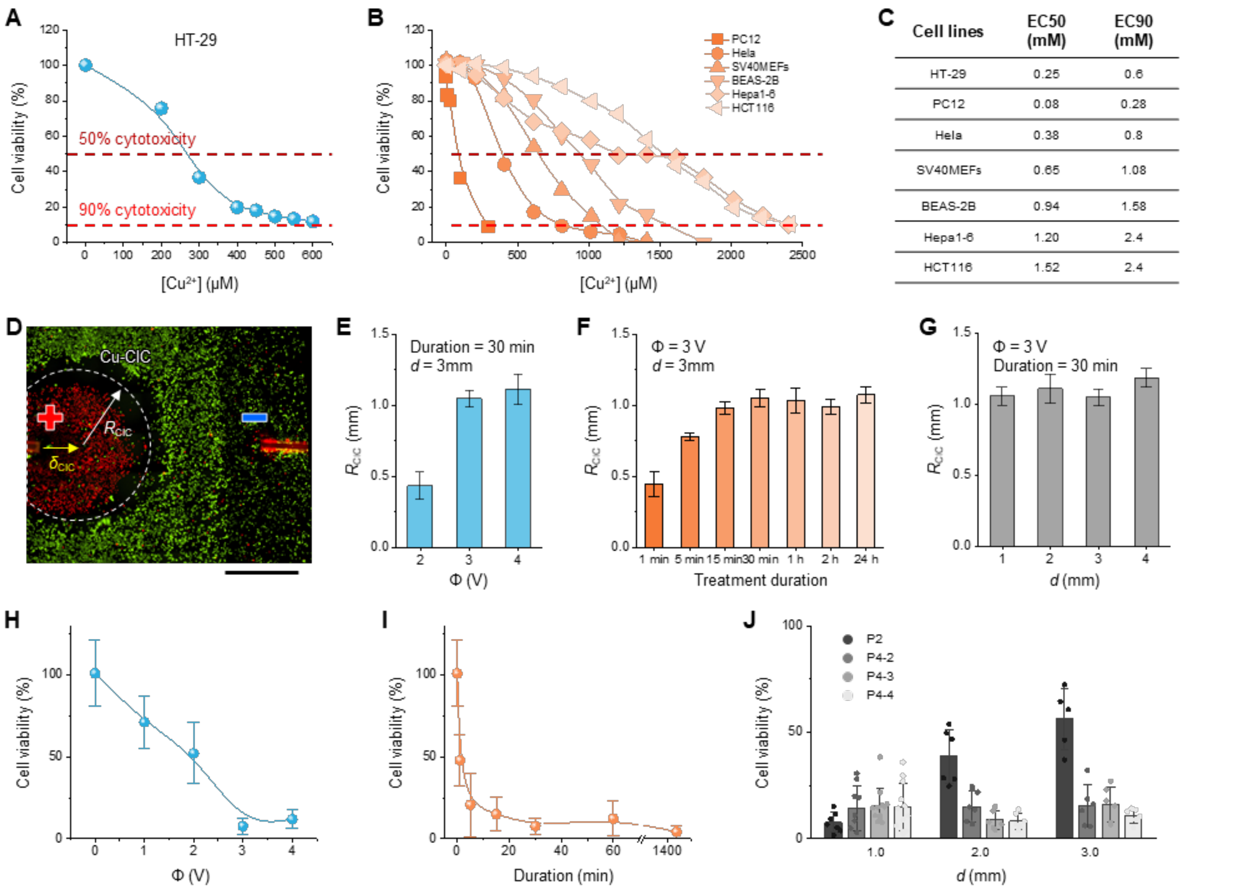

Fig. S9. In vitro characterization of formation and anticancer effect of Cu-CICs for HT-29 monolayer cells. (**A**) A typical viability curve of HT-29 monolayer cancer cells exhibiting the critical concentration for 50% (EC50 ≈ 0.25 mM) and 90% (EC90 ≈ 0.6 mM) maximal effective cuproptotic cell death induced by overdosed Cu^{2+} ions. Data were obtained and reproduced from (*42*). (**B**) Typical viability curves of various cell lines including PC12 (rat adrenal cancer cell line), Hela (human cervical cancer cell line), SV40MEF (mouse embryonic fibroblast cell line), BEAS-2B (human bronchial epithelial cell line), Hepa1-6 (mouse hepatocellular carcinoma cell line), and HCT116 (human colorectal carcinoma cell line) under cuproptotic treatments of overdosed Cu^{2+} ions. Data were obtained and reproduced from (*41*). (**C**) A summary table of EC50 and EC90 values of overdosed Cu^{2+}-based cuproptotic treatments for various cell lines described above. (**D**) A representative live/dead fluorescence image of HT-29 monolayer cells after 30-min M-cuproptosis treatment using the P2 configuration. The well-defined circular boundary of the area of cytotoxicity, highlighted by red, provide evidence of the Cu-CIC formation and its anticancer effect. Scale bar, 1 mm. (**E** to **G**) Experimental characterization of R_{CIC} as a function of Φ (E), treatment duration (F), and characteristic length, *d*, of the target area (G). The invariance of R_{CIC} over treatment duration (≥30 min) and *d* stems respectively from the asymptotic saturation of Cu^{2+} production, as shown in Fig. 2B, and from the characteristic profile of E-fields. (**H** to **J**) Anticacer efficacy evaluation of M-cuproptosis by assessing the viability of HT-29 cells after treatment with variable factors such as input voltage, Φ (H), treatment duration (I), electrode configuration and target TME area, *d*, (J).

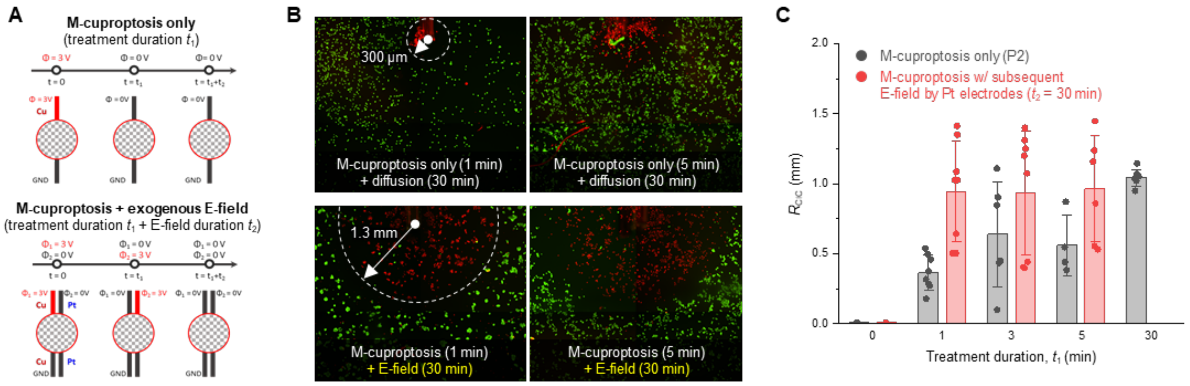

Fig. S10. In vitro characterization of the exogenous E-field effect on Cu-CIC dynamics. (**A**) Experimental setup and protocol. We positioned an additional pair of Pt electrodes (P2 configuration) right next to the Cu electrodes, so as to apply an equivalent E-field without Cu^{2+} production. We first applied $\Phi = 3$ V to Cu electrodes for t_1 (= 0, 1, 3, 5, and 30 min), and then switched to Pt electrodes for t_2 (= 30 min). (**B**) Representative fluorescent images showing live/dead viability assay results of the exogenous E-field effect on Cu-CIC dynamics and the corresponding therapeutic area. Scale bar, 1 mm. (**C**) Comparative analysis of R_{CIC} evolution after the treatment sequences ($t_1 + t_2$) described in (A).

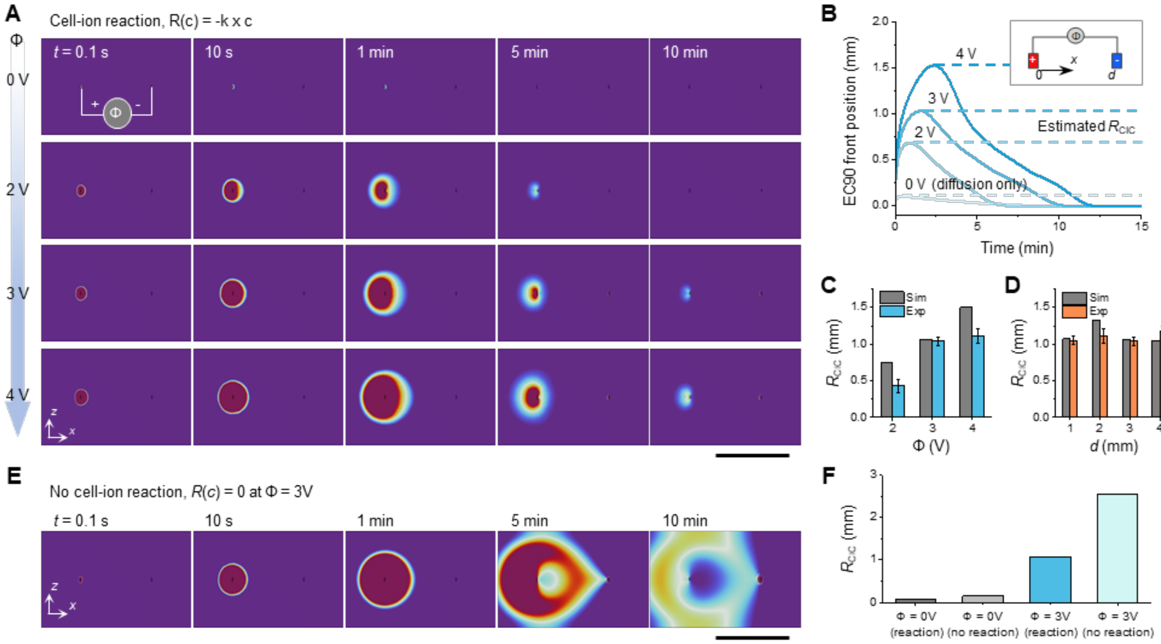

Fig. S11. Numerical analysis of Cu-CIC dynamics within monolayer cells. (**A**) Numerical results of Cu-CIC kinetics with increasing Φ under the continuous cell-ion reaction (that is, intracellular Cu^{2+} uptake) condition expressed by $R(c) = -kc$ where k is the first-order rate constant for passive diffusion. For simplicity of analysis, the P2 configuration is chosen with a fixed $d = 3$ mm. Modified $\dot{M}(t)$ functions, named $C(t)$—given by $C_2(t) = 294082.2/(t + 361.67)^2$, $C_3(t) = 587428.7/(t + 438.91)^2$, $C_4(t) = 668222.3/(t + 389.56)^2$ respectively for Φ = 2, 3, and 4 V— are assigned to the surface of Cu anode as the input boundary conditions of [Cu^{2+}] (see note S1 for details on mathematical modeling and fitting); whilst other Cu surfaces are insulated. $C_0(t)$—which must be zero in the real space— is set equal to $C_3(t)$ in order to estimate and directly compare the effect of pure diffusion with electrochemically released Cu^{2+} ions at Φ = 3 V. (**B**) Kinetic evolution of the EC90 front of Cu-CICs with increasing Φ. Given the continuous cell-ion reaction condition, the maximum reach can be estimated as R_{CIC}. (**C** and **D**) Comparison of R_{CIC} between simulation and experimental results for increasing Φ (C) and d (D). (**E**) Numerical results of Cu-CIC kinetics at Φ = 3 V without the cell-ion reaction boundary condition. (**F**) Numerical estimation of R_{CIC}, based on Cu-CIC kinetics, showing the apparent effect of the application of E-fields (electromigration effect) and the boundary condition for cell-ion reaction. All scale bars indicate 3 mm.

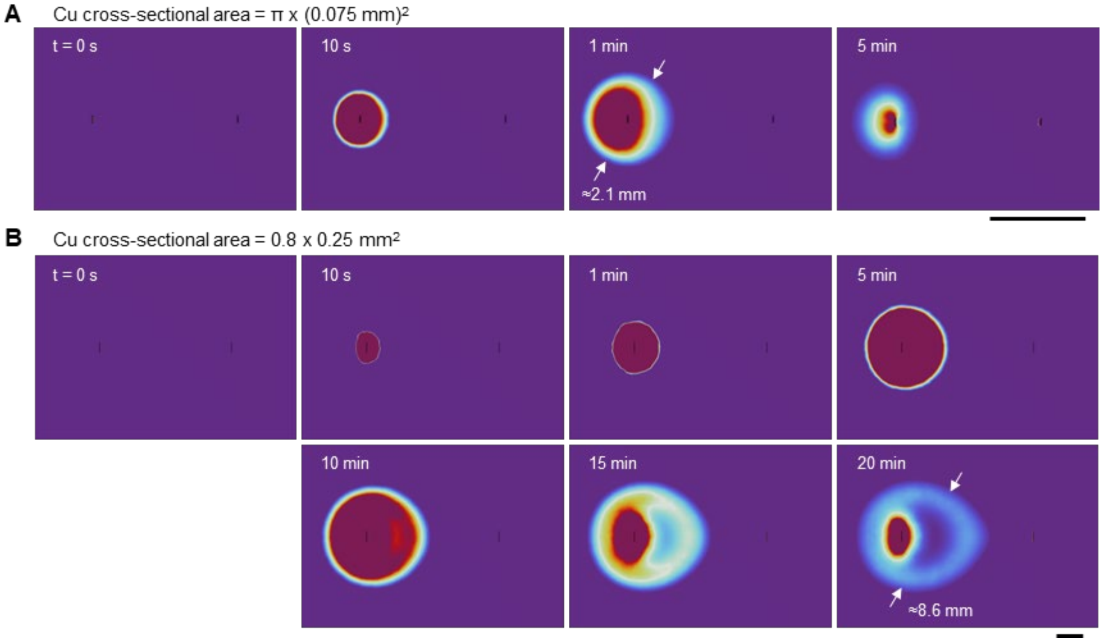

Fig. S12. Numerical analysis of Cu-CIC dynamics with different Cu pad types. Numerical results of Cu-CIC kinetics under the continuous cell-ion interaction condition for the circular anode (diameter, 150 μm) (**A**) and the large rectangular anode (dimension, 0.8×0.25 mm^2) (**B**). The rectangular one is identical to that used in the actual microrobot. All scale bars indicate 2 mm.

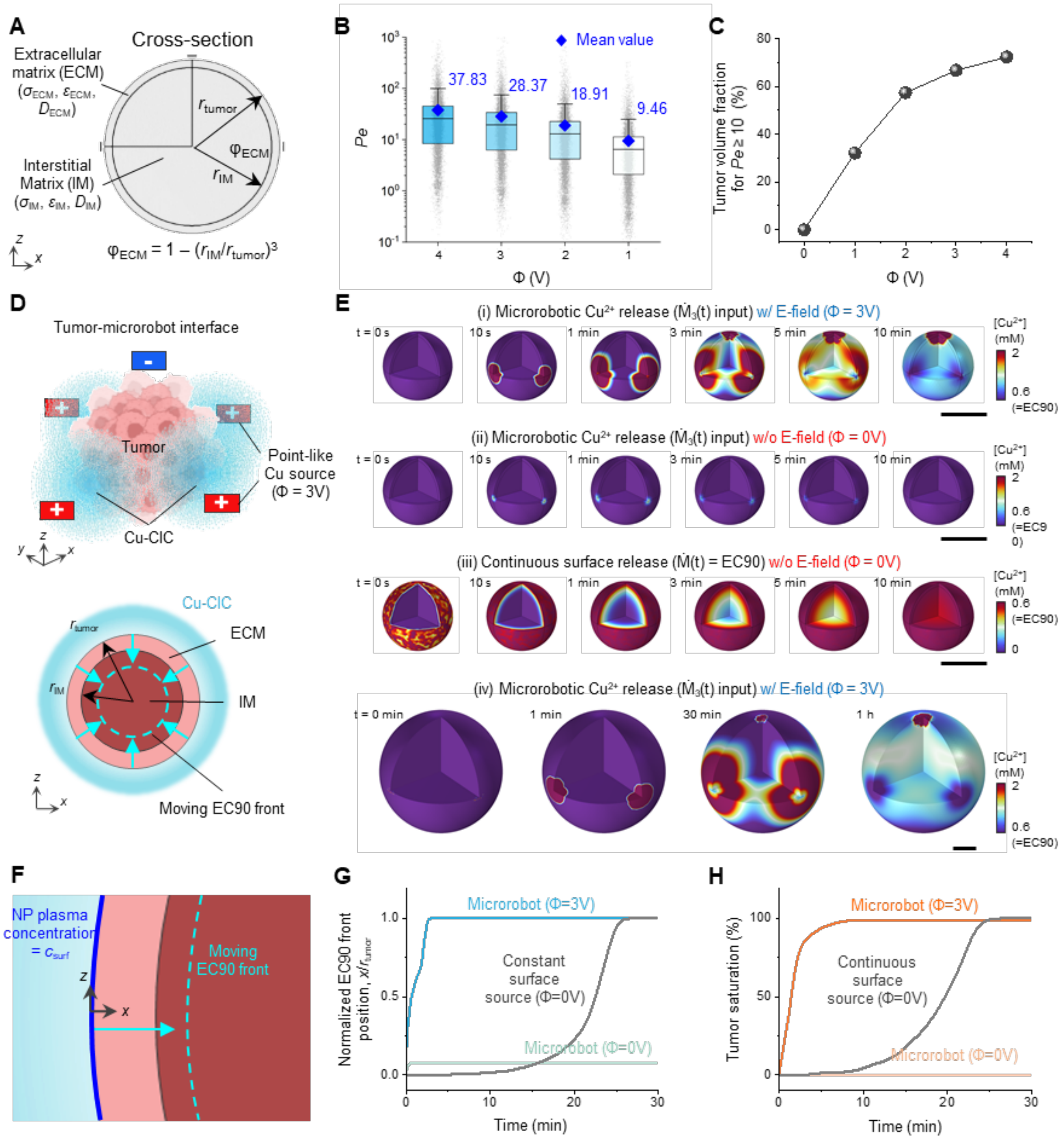

Fig. S13. Numerical analysis of Cu-CIC dynamics within the spherical core-shell tumor model. (**A**) Model definition used in the numerical study. The core and shell of the tumor model correspond respectively to the interstitial matrix (IM) and extracellular matrix (ECM), whose volume fraction is defined as $\phi_{ECM} = 1–(r_{IM}/r_{tumor})^3$ with r_{tumor} = 1.45 mm which beset suits the Cu configuration with d = 3 mm (see fig. S9). The effective medium theory based on the dual-scale cylindrical pore model (*47*) and the modified Brinkman model (*48*) are employed to characterize the diffusive trait within IM and ECM, respectively. See table S1 for the actual values used for the material properties and geometric parameters. (**B** and **C**) Integrative data analysis of *Pe* (B) and the concomitant tumor volume fraction satisfying $Pe \geq 10$ (C) for Φ = 1–4 V. (**D**) Schematic illustration of the microrobot-mediated Cu^{2+}-rich electrochemical interfacing of the tumor (top) and the shrinking core model (SCM) describing tumor penetration/saturation based on the moving EC90 front (bottom). (**E**) Time-evolving spatial distribution of Cu^{2+} ions within the spherical core-shell tumor model (r_{tumor} = 1.45 and 5 mm, respectively for (i-iii) and (iv), and ϕ_{ECM} = 0.25) during M-cuproptosis at Φ = 3 V (i), 0 V (ii). To investigate the effect of

electromigration on tumor penetration/saturation in comparison with pure diffusion, the input boundary condition of four-point $\dot{M}_3(t)$ was equally applied to the model of $\Phi = 0V$ (ii), or the constant surface concentration ($[Cu^{2+}]$ = EC90 = 0.6 mM) was assumed to meet the central condition of the SCM without an exogenous E-field (iii). Note that for the larger tumor model (r_{tumor} = 5 mm; volume ~520 mm^3 as shown in (iv)), the input function was replaced with $\beta M_3(t)$ where β is the fold increase in the cross-sectional area of Cu sources; for example, the experimental value is $\beta = (0.25 \times 0.8\ mm^2)/(\pi \times 0.075^2\ mm^2) \approx 11.32$. The continuous cell-ion reaction condition, as described in fig. S11, is applied for all cases. Scale bars, 2 mm. (**F**) Enlarged schematic view of the cross-sectional tumor surface area describing the model assumption, such as the boundary condition and the moving EC90 front. (**G** and **H**) Kinetic evolution of the EC90 front (G) and pharmacokinetic fractional tumor saturation (H) of Cu^{2+} ions injected from the source types described in (**E**).

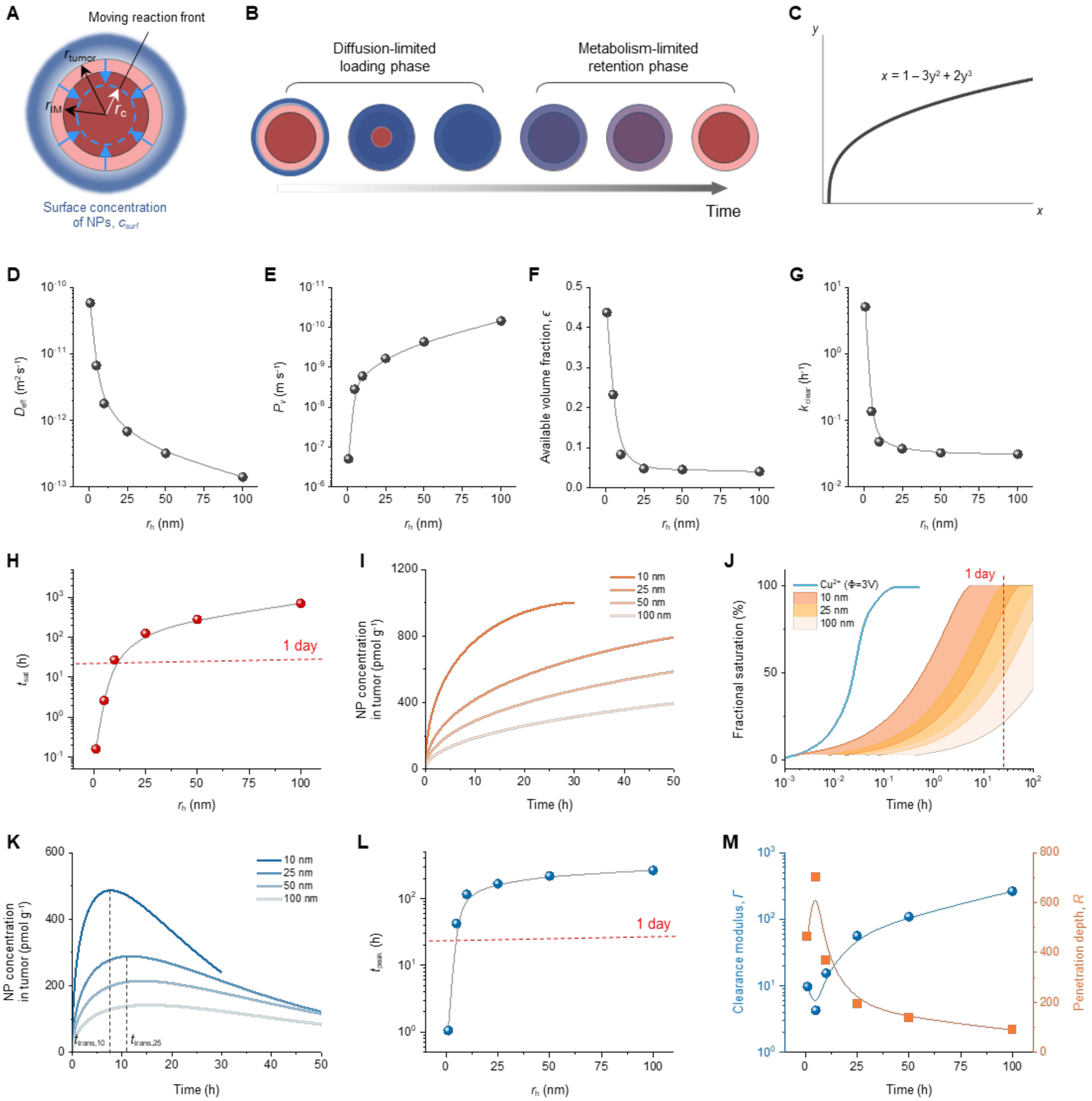

Fig. S14. Theoretical analysis of tumor penetration and saturation for systemically administered nanoparticles. (**A**) Schematic illustration of the nanoparticle (NP)-rich tumor microenvironment under the assumption of the SCM (see note S5). (**B**) Schematic drawing of the kinetics of tumor saturation by diffusion-limited NPs whose concentration, reaction, and moving front are characterized by the critical radius, r_c. The kinetic phase is bifurcated into the diffusion-limited loading phase and the metabolism-limited retention phase, depending on the initial boundary condition and the clearance term. (**C**) Characteristic curve of the SCM, expressed by $x = 1 - 3y^2 + 2y^3$ as described in eq. S37, where x and y denote t/t_{sat} and r_c/r_{tumor}, respectively. (**D** to **G**) Plot of size-dependent parameters involved in tumor uptake: diffusivity (D), tumor vascular permeability (P_v), available volume fraction (ϵ), and plasma clearance factor (k_{clear}). (**H**) Characteristic time scale to saturate the tumor with a radius of r_{tumor} = 1.45 mm for NPs of clinically relevant sizes (r_{h} = 1–100 nm). (**I**) Pharmacokinetic profiles for tumor uptake of NPs (r_{h} = 10–100 nm) estimated from the SCM. (**J**) Fractional tumor saturation behavior of Cu^{2+} ions (Φ = 3V) in comparison with NPs (r_{h} = 10–100 nm). (**K**) Modified pharmacokinetic profiles for

tumor uptake of NPs (r_h = 10–100 nm) with consideration of clearance. (**L** and **M**) Plots of estimated t_{peak} (L), clearance modulus, $\Gamma = t_{sat}/t_{clear}$, and penetration depth as a function of r_h (M) .

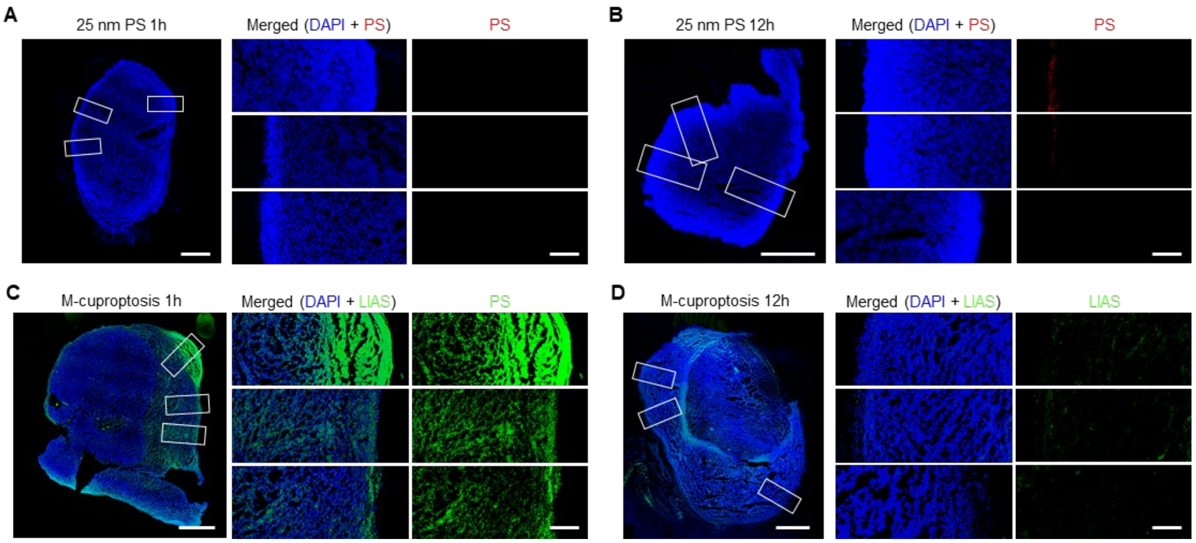

Fig. S15. Tumor penetration analysis. (**A** and **B**) Representative fluorescence images of excised tumors incubated for 1 h (A) and 12 h (B) in PS nanoparticle solution. (**C** and **D**) Representative fluorescence images of excised tumors treated by M-cuproptosis for 1 h (C) and 12 h (D). Clipped images show the sections used for intensity profiling. Scale bars are 1 mm for the tumor images in A to D, and 200 μm in the clipped images.

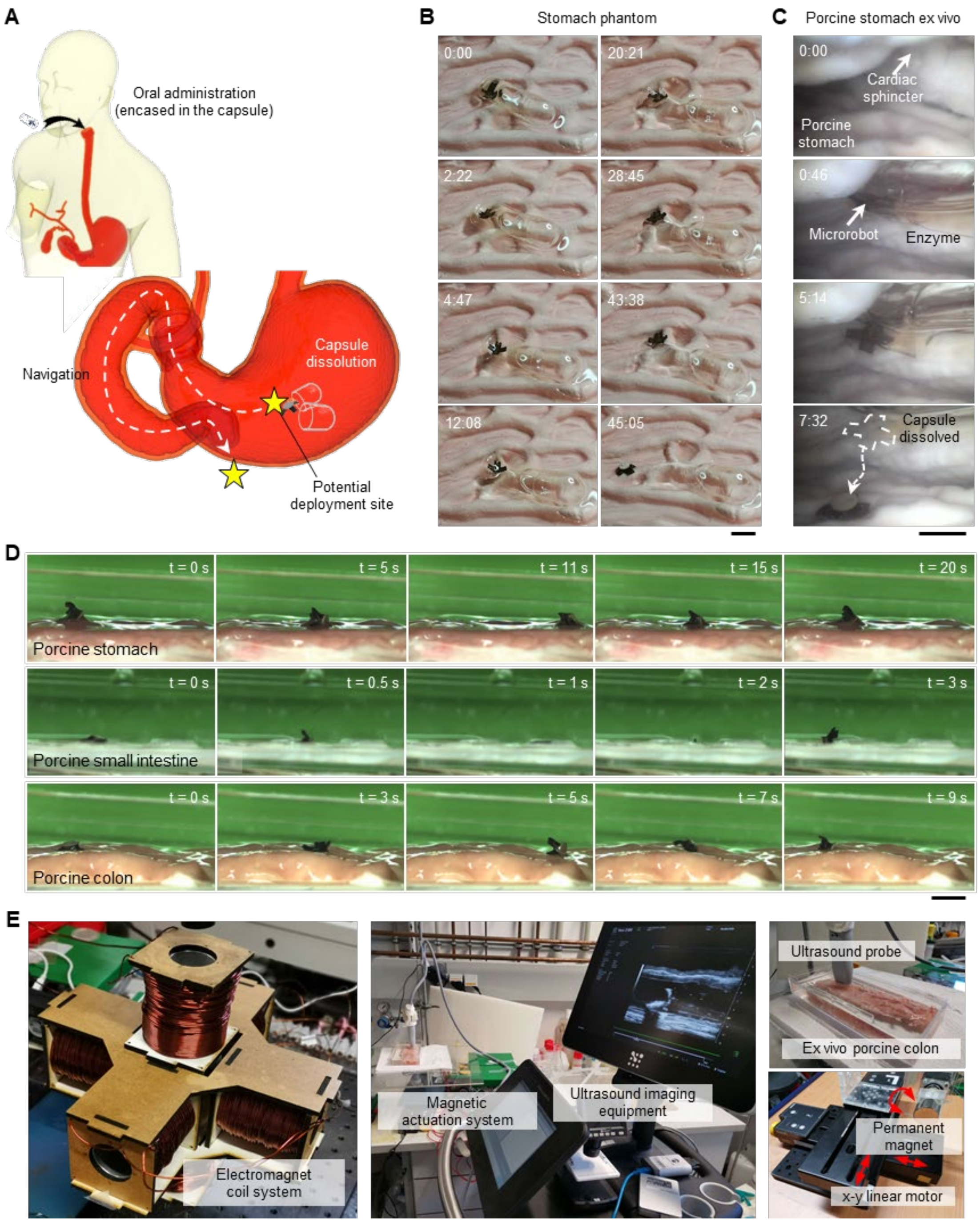

Fig. S16. Capsule-mediated deployment and magnetically driven locomotion of the microrobot. (**A**) An overview of the capsule-mediated deployment strategy. Different capsule types specified for each organ environment can be employed to carry the microrobot to the target site of action. For example, a gelatin capsule is properly dissolvable in the stomach environment, and while an enteric capsule is more effective to target the small intestine directly. (**B** and **C**) Sequential image frames of the capsule-mediated deployment process performed in the phantom (movie S3) (B) and ex vivo porcine stomach model (movie S4) (C). (**D**) Ex vivo characterization of locomotion performance on the GI lining (movie S2). (**E**) Experimental setup for locomotion characterization. All scale bars indicate 5 mm.

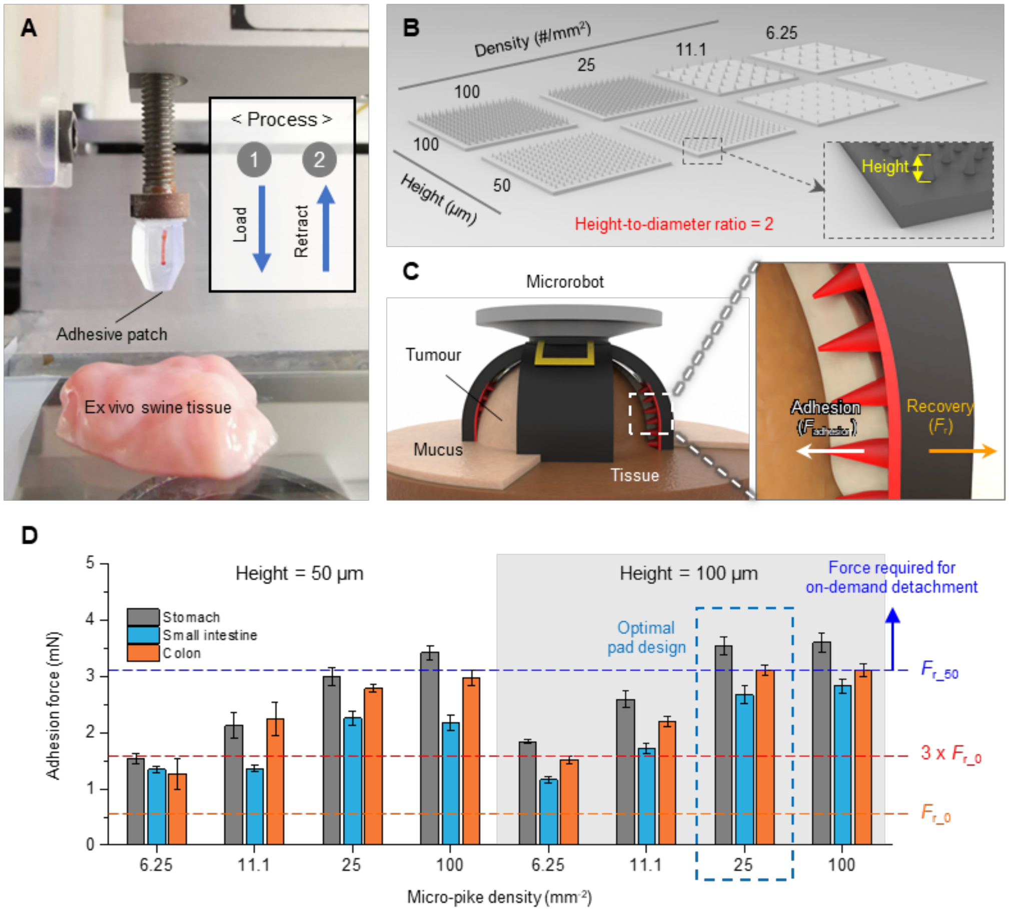

Fig. S17. Experimental setup and characterization of micro-spike bioadhesive pads ex vivo. (**A**) Experimental setup for ex vivo adhesion characterization with swine tissues. (**B**) Schematic drawing of 3D printed micro-spike bioadhesive pads with varying density and height. (**C**) Schematic drawing of bioadhesive-assisted anchoring of the microrobot. The inset image shows the factors determining adhesion stability. (**D**) Ex vivo adhesion characterization of micro-spike pads for swine stomach, small intestine, and colon tissues.

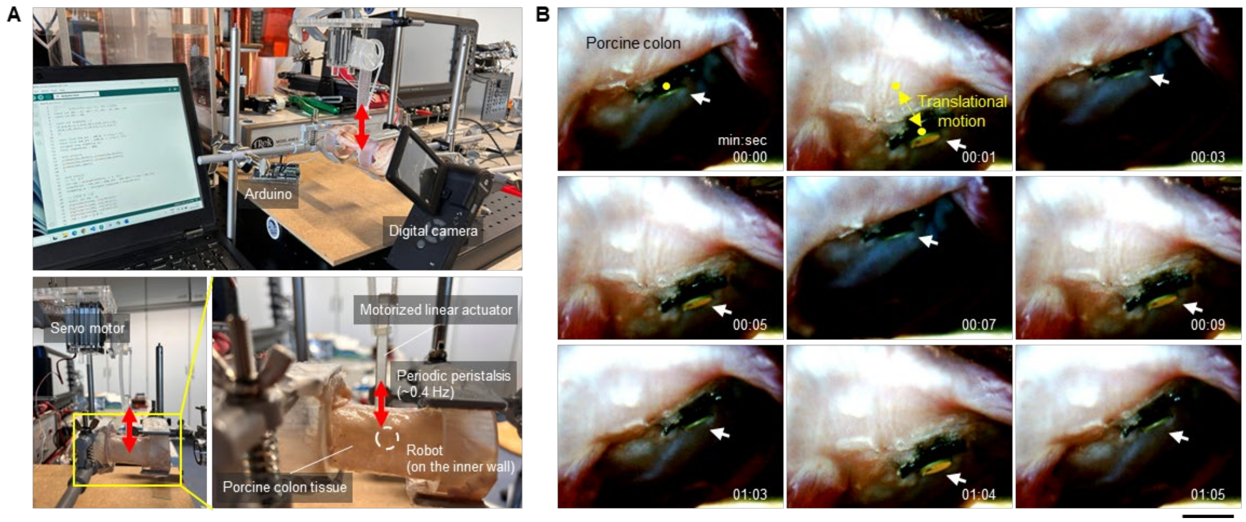

Fig. S18. Ex vivo adhesion test under accelerated peristalsis conditions. (**A**) Experimental setup of motorized accelerated peristalsis motion (~0.4 Hz) of porcine colon tissues. (**B**) Sequential image frames of peristaltic motion of the colon tissue with the microrobot adhered onto the inner wall (see movie S6). Scale bar, 5 mm.

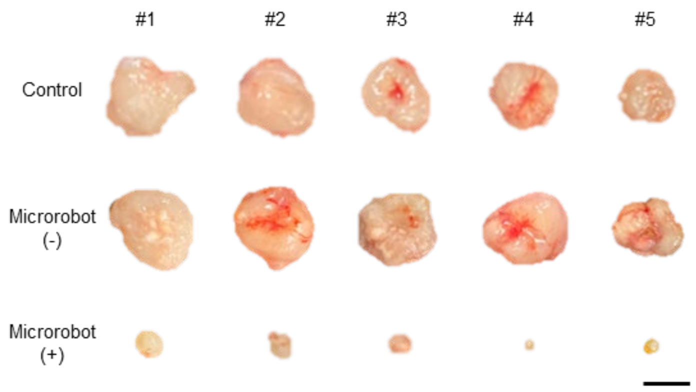

Fig. S19. In vivo therapeutic outcome. Photographs of the tumor control group, microrobot (-), and microrobot (+) groups on day 14 post-treatment with M-cuproptosis. Scale bar, 5 mm.

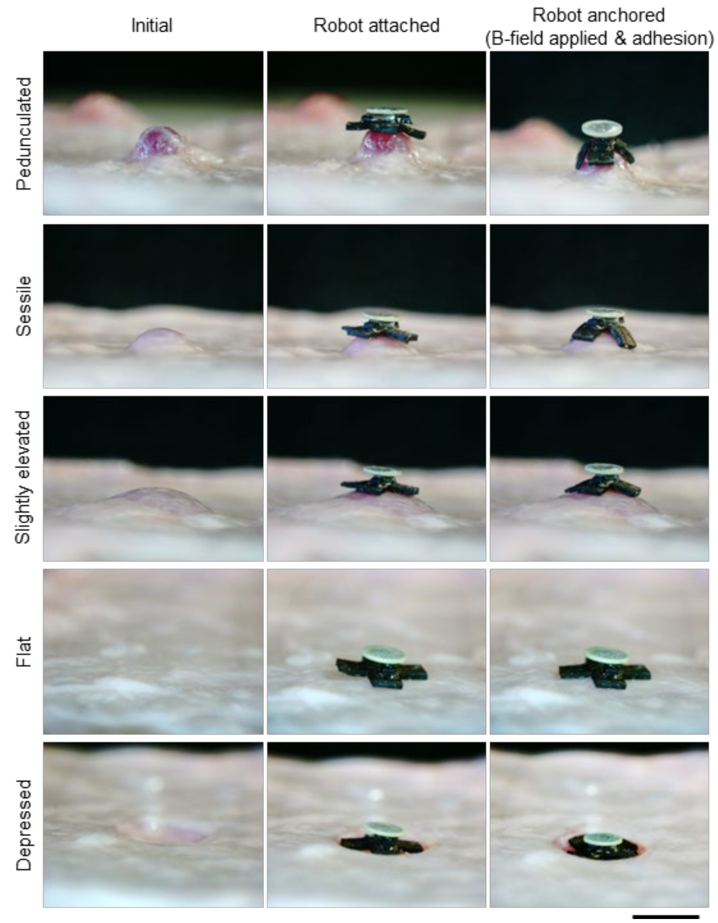

Fig. S20. The microrobot can be anchored to either polypoid or non-polypoid GI tumors. Scale bar, 5 mm.

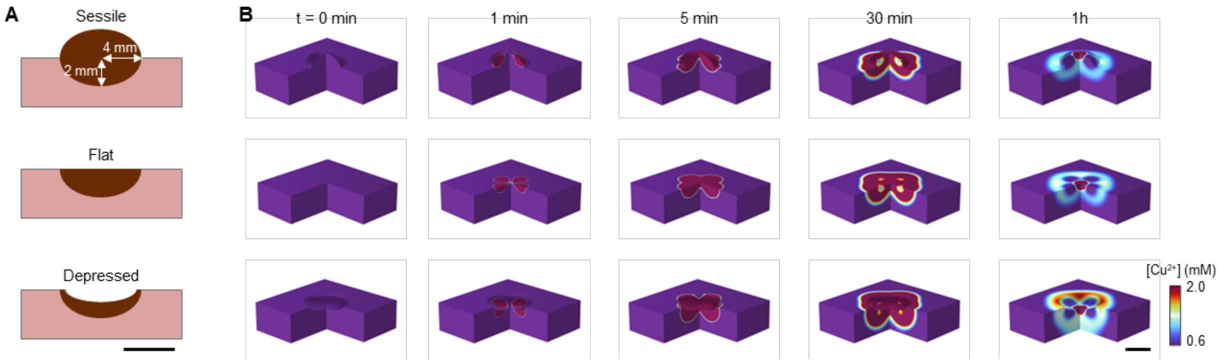

Fig. S21. Numerical analysis of Cu-CIC dynamics for various tumor types. (**A**) Schematics of representative tumor types including polypoid (sessile, top) and non-polypoid (flat, middle; depressed, bottom) cancers. (**B**) Numerical results of Cu-CIC kinetics under the continuous cell-ion interaction condition for the cases shown in A. All scale bars indicate 5 mm.

Table S1. Definition of parameter values.

Model category	Symbol	Description (unit)	Value	Note
Electromigration-dominated ion kinetics (note S1)	c	Concentration (mM)	-	-
	$[Cu^{2+}]$	Cu^{2+} concentration (mM)	-	-
	D	Diffusivity ($m^2\ s^{-1}$)	10^{-9}	For Cu^{2+}
	T	Absolute temperature (K)	310	Body temperature (≈ 37 ˚C)
	E	Electric field ($V\ m^{-1}$)	$0–10^4$	-
	Φ	Electric potential (V)	0–4	-
	z	Valence	2	-
	e	Elementary charge (C)	1.602×10^{-19}	-
	k	First-order reaction rate constant (s^{-1})	4.70×10^{-4}	~4–7 × $10^{-6}\ s^{-1}$ for monolayer cells (*44*)
	$\mathrm{M}(t)$	Cu^{2+} quantity function (µg)	-	-
	$\dot{\mathrm{M}}(t)$	Cu^{2+} release rate function ($nmol\ s^{-1}$)	-	-
	d	Diameter of target area (mm)	1–4	Characteristic length of the system
	μ	Ionic mobility ($m^2\ V^{-1}\ s^{-1}$)	3.78×10^{-8}	For Cu^{2+}, from the Einstein relation
	u	Drift velocity ($m\ s^{-1}$)	$0–10^4$	$u = \mu E$
	r_h	Hydrodynamic radius (nm)	0.065	For Cu^{2+}, from the Einstein relation
	η	Dynamic viscosity (Pa s)	6.913×10^{-4}	At T = 310 K (≈ 37 ˚C)
	Pe	Peclet number	$0–10^3$	-
	σ	Electrical conductivity of the tumor ($S\ m^{-1}$)	1	-
	ε_r	Relative permittivity of the tumor	60	-
	r_{tumor}	Radius of the tumor (mm)	1.45	-
	r_{IM}	Radius of the interstitial matrix (mm)	1.3174	Calculated from $\phi_{ECM} = 0.25$
	ϕ_{ECM}	ECM volume fraction	0.25	-
Intracellular ion uptake (note S1)	υ	Rate of intracellular uptake (ions min^{-1})	-	-
	K_m	Michaelis constant (mM)	$4.7–6.5 \times 10^{-3}$	(*44*)
	V_{max}	Limiting rate of facilitated diffusion ($mM\ min^{-1}$)	0.01–0.02	(*44*)
	Ψ	Electrochemical potential ($J\ mol^{-1}$)	-	-
	J	Ionic flux across plasma membrane ($mol\ m^{-2}\ s^{-1}$)	-	-
	V_m	Membrane potential (mV)	-50	Defined as $\Phi_{in} - \Phi_{out}$
	δ_m	Membrane thickness (nm)	5	(83)
	P_m	Membrane permeability ($m\ s^{-1}$)	$2–20 \times 10^{-8}$	Defined as D/δ_m
	ρ_{cell}	Density of cells ($cells\ mm^{-2}$)	~800	HT-29 monolayers
Reaction equation (note S2)	E^0	Standard reduction potential (V)	0.34	for Cu^{2+}
	ΔE^0	Standard potential for reaction (V)	-	Defined as $E^0_{cathode} - E^0_{anode}$
	Φ_{onset}	Onset potential for Cu^{2+} dissolution (V)	1.4	Experimental value
Tumor interstitial diffusivity (note S4)	P	Permeability of the tumor interstitial space ($m\ s^{-1}$)	-	-
	ϵ	Available volume fraction in the tumor	-	-
	λ	Ratio of molecular radius to pore radius	-	Defined as r_h/r_{pore}
	κ	Hydrodynamic drag factor of a spherical particle	-	Obtained from numerical solutions[68]
	r_{gel}	Pore radius of the ECM fiber matrix (µm)	2	(*48*)
	ϕ_{gel}	Volume fraction of the collagen gel matrix	0.378	(*48*)
	ϵ_{gel}	Gel porosity	0.622	Defined as $1 - \phi_{gel}$
	k_{gel}	Kozeny factor	-	-
Tumor uptake of nanoparticles (note S5)	P_v	Tumor vascular permeability ($m\ s^{-1}$)	-	-
	k_{clear}	Plasma clearance factor (h^{-1})	-	-
	r_c	Critical radius for tumor saturation	-	-
	$[Ag]_t$	Antigen concentration (mM)	8.303×10^{-5}	(*50*)
	c_{surf}	Plasma concentration of nanoparticles at the tumor surface (mM)	0.001	(*47,93–95*)
	t_{sat}	Characteristic time scale for tumor saturation (h)	-	-
	t_{peak}	Characteristic time scale for peak tumor uptake (h)	-	-
	t_{clear}	Characteristic time scale for clearance (h)	-	-
	K_d	Binding affinity of nanoparticles (mM)	10^{-6}	(*47*)
	r_{cap}	Radius of tumor capillary (µm)	8	(*47*)
	R_{Krogh}	Average radius of tumor tissues surrounding each blood vessel (µm)	75	(*47*)
	Γ	Clearance modulus	4–260	For r_h = 1–100 nm
Magnetic actuation (note S7)	K_{eff}	Effective bending stiffness ($mN\ mm^2$)	-	-
	E	Young's modulus (Pa)	-	-
	I	Moment of inertia ($g\ mm^2$)	-	-
	A	Cross-sectional area of the beam (mm^2)	-	-
	w	Beam deflection (mm)	-	-
	F_r	Beam recovery force (mN)	-	-
	$\mathbf{M}(s)$	Parameterized magnetic moment ($mN\ mm\ mT^{-1}$)	-	-
	$\mathbf{B}(t)$	Magnetic field (mT)	-	-
	$\mathbf{R}$	Rotation matrix	-	-
	$\boldsymbol{\tau}_{net}$	Net magnetic torque (mN mm)	-	-
	$\mathbf{M}_{net}$	Net magnetic moment ($mN\ mm\ mT^{-1}$)	-	-
	$F_{adhesion}$	Adhesion force (mN)	-	-

Table S2. Experimental data set of diffusivity for interstitial matrix.

Tracer molecules	Hydrodynamic radius, r_h (nm)	Diffusivity, D ($m^2 s^{-1}$)	Reference
Na-F	0.48	3.782E-11	(*47*)
	0.48	4.595E-11	
	0.48	1.167E-10	
FITC-BSA	3.576	1.691E-12	(*47*)
	3.576	2.850E-13	
	3.576	2.026E-13	
FITC-dextran	3.153	1.383E-11	(*47*)
	4.393	7.837E-12	
	5.437	3.457E-12	
	3.148	4.365E-12	
	3.231	3.288E-12	
	4.376	3.463E-13	
	4.651	4.156E-13	
	5.406	1.158E-13	
	5.803	9.587E-14	
Na-FITC	0.45	6.40E-10	(*47*)
	0.45	4.30E-10	
	0.45	5.35E-10	
Dextran	3.2	7.50E-11	(*47*)
	5	4.20E-11	
	6.5	1.90E-11	
	22.1	2.47E-12	
	22.1	4.11E-13	
	19.2	1.44E-12	
Lactalbumin	2.54	1.01E-10	(*47*)
Fab	3.36	2.70E-11	(*47*)
IgG	4.85	1.30E-11	(*47*)
	4.85	9.60E-13	
	4.85	1.90E-11	
	4.85	1.90E-11	
	4.85	8.70E-12	
	4.85	1.91E-11	
	4.85	9.38E-12	
	4.85	1.27E-11	
IgM	8.81	7.70E-12	(*47*)
	8.81	1.05E-11	
	8.81	4.30E-12	
	8.81	7.50E-12	
Liposome	76.2	2.97E-13	(*47*)
Lactalbumin, BSA, IgG, IgM, dextran, liposomes	0.40	6.351E-10	(*17*)
	3.88	6.776E-11	
	5.95	1.476E-11	
	8.95	8.503E-12	
	18.79	3.374E-13	
	1.925	1.653E-10	
	3.918	3.719E-11	
	5.889	3.061E-11	
	8.852	1.764E-11	
	74.631	4.979E-13	
	3.877	5.313E-11	
	8.852	6.560E-12	
	18.786	1.050E-12	
	3.877	4.821E-11	
	8.945	1.764E-11	
	18.786	4.033E-12	
	73.855	6.776E-13	
Au nanoparticle (active targeting)	15	3.94E-15	(*87*)
	30	1.09E-15	
	60	2.81E-15	
	100	1.07E-15	
Au nanoparticle (passive targeting)	15	6.19E-15	(*87*)
	30	1.39E-14	
	60	1.25E-14	
	100	6.94E-17	

Table S3. Experimental data set of diffusivity for extracellular matrix.

Tracer molecules	Hydrodynamic radius, r_h (nm)	Diffusivity, D (m^2 s^{-1})	ECM type	Reference
Polystyrene nanoparticle	267.2	9.20E-13	0.5 mg/ml collagen(I) matrix	(*13*)
	267.2	5.90E-13	1 mg/ml collagen(I) matrix	
	267.2	3.50E-13	2 mg/ml collagen(I) matrix	
RITC-silica nanoparticle	52.06	2.67E-12	0.7 mg/ml collagen(I) matrix	(*13*)
	52.06	9.20E-13	1 mg/ml collagen(I) matrix	
	52.06	5.20E-13	1.5 mg/ml collagen(I) matrix	
BSA, dextran, liposome	1.83	1.303E-10	1% collagen matrix	(*48*)
	3.91	5.768E-11	1% collagen matrix	
	5.58	3.537E-11	1% collagen matrix	
	23.77	5.426E-12	1% collagen matrix	
	25.90	5.139E-12	1% collagen matrix	
	49.66	1.881E-12	1% collagen matrix	
	1.83	1.201E-10	3% collagen matrix	
	3.91	4.052E-11	3% collagen matrix	
	5.65	2.623E-11	3% collagen matrix	
	6.88	1.793E-11	3% collagen matrix	
	23.77	2.337E-12	3% collagen matrix	
	1.80	9.407E-11	4.5% collagen matrix	
	2.19	7.169E-11	4.5% collagen matrix	
	3.50	3.350E-11	4.5% collagen matrix	
	3.86	3.537E-11	4.5% collagen matrix	
	4.58	1.894E-11	4.5% collagen matrix	
	23.77	7.673E-13	4.5% collagen matrix	
BSA	4	2.200E-11	3% collagen matrix	(88)
Dextran	6	2.000E-11	3% collagen matrix	(88)
Au nanoparticle	50	1.62E-12	collagen(I) matrix	(*44*)
	120	3.39E-13	collagen(I) matrix	
	6	7.60E-11	ECM material	(89)
	6	9.00E-11	ECM material	

Table S4. Figure of merits of E-field-assisted locoregional cancer therapy.

Treatment type	Cancer-killing mechanism	Invasiveness (method)	Localization (spot size, mm)	Local power density (W cm^{-2})	Duration (min)	Temperature increase (℃)	Reference
M-cuproptosis (This work)	Cuproptosis	Non-invasive (wireless microrobot)	●●● (1–5)	10^{-5}–10^{-6}	30–360	~0	-
RF/MW electrothermal ablation	Thermal coagulation necrosis	Invasive (tethered electrodes)	● (3–50)	1.5–8	5–12	5–65	(*51, 99–101*)
Electrochemical therapy	Electrolysis-induced local pH changes	Invasive (tethered electrodes)	●● (5–30)	10^{-3}–0.1	60–240	~0	(*52, 102, 103*)
Irreversible electroporation	Electric pulse-induced disruption of cell membrane	Invasive (tethered electrodes)	●● (5–30)	0.05–3.75	0.2–5	5–30	(*53, 104*)
Tumor-treating fields	Mitotic spindle disruption	Non-invasive (wearable electrodes)	- (~100)	0.2–1.5	1,000–1,500	<5	(*54, 105*)

Captions for Supplementary Videos

Movie S1. Effect of microrobot design on locomotion capability on the gastrointestinal lining.

Movie S2. Characterization of rolling locomotion within gastrointestinal tract tissues.

Movie S3. Demonstration of the deployment procedure in the stomach phantom.

Movie S4. Demonstration of locomotion-driven long-distance gastrointestinal navigation ex vivo.

Movie S5. Demonstration of consecutive tumor targeting and anchoring ex vivo.

Movie S6. Demonstration of adhesion under accelerated test conditions of peristalsis ex vivo.

REFERENCES

1. J. Shi, P. W. Kantoff, R. Wooster, O. C. Farokhzad, Cancer nanomedicine: Progress, challenges and opportunities. *Nat. Rev. Cancer* **17**, 20–37 (2017).

2. D. Peer, J. M. Karp, S. Hong, O. C. Farokhzad, R. Margalit, R. Langer, Nanocarriers as an emerging platform for cancer therapy. *Nat. Nanotechnol.* **2**, 751–760 (2007).

3. R. K. Jain, T. Stylianopoulos, Delivering nanomedicine to solid tumors. *Nat. Rev. Clin. Oncol.* **7**, 653–664 (2010).

4. D. Rosenblum, N. Joshi, W. Tao, J. M. Karp, D. Peer, Progress and challenges towards targeted delivery of cancer therapeutics. *Nat. Commun.* **9**, 1410 (2018).

5. S. Wilhelm, A. J. Tavares, Q. Dai, S. Ohta, J. Audet, H. F. Dvorak, W. C. W. Chan, Analysis of nanoparticle delivery to tumors. *Nat. Rev. Mater.* **1**, 16014 (2016).

6. T. Stylianopoulos, R. K. Jain, Design considerations for nanotherapeutics in oncology. *Nanomedicine* **11**, 1893–1907 (2015).

7. D. A. Hume, The mononuclear phagocyte system. *Curr. Opin. Immunol.* **18**, 49–53 (2006).

8. A. Albanese, C. D. Walkey, J. B. Olsen, H. Guo, A. Emili, W. C. W. Chan, Secreted biomolecules alter the biological identity and cellular interactions of nanoparticles. *ACS Nano* **8**, 5515–5526 (2014).

9. F. Yuan, M. Dellian, D. Fukumura, M. Leunig, D. A. Berk, V. P. Torchilin, R. K. Jain, Vascular permeability in a human tumor xenograft: Molecular size dependence and cutoff size. *Cancer Res.* **55**, 3752–3756 (1995).

10. V. P. Chauhan, T. Stylianopoulos, J. D. Martin, Z. Popović, O. Chen, W. S. Kamoun, M. G. Bawendi, D. Fukumura, R. K. Jain, Normalization of tumour blood vessels improves the delivery of nanomedicines in a size-dependent manner. *Nat. Nanotechnol.* **7**, 383–388 (2012).

11. S. Lee, H. Han, H. Koo, J. H. Na, H. Y. Yoon, K. E. Lee, H. Lee, H. Kim, I. C. Kwon, K. Kim, Extracellular matrix remodeling in vivo for enhancing tumor-targeting efficiency of

nanoparticle drug carriers using the pulsed high intensity focused ultrasound. *J. Control. Release* **263**, 68–78 (2017).

12. C. Wong, T. Stylianopoulos, J. Cui, J. Martin, V. P. Chauhan, W. Jiang, Z. Popovic, R. K. Jain, M. G. Bawendi, D. Fukumura, Multistage nanoparticle delivery system for deep penetration into tumor tissue. *Proc. Natl. Acad. Sci. U.S.A.* **108**, 2426–2431 (2011).

13. X. He, Y. Yang, Y. Han, C. Cao, Z. Zhang, L. Li, C. Xiao, H. Guo, L. Wang, L. Han, Z. Qu, N. Liu, S. Han, F. Xu, Extracellular matrix physical properties govern the diffusion of nanoparticles in tumor microenvironment. *Proc. Natl. Acad. Sci. U.S.A.* **120**, e2209260120 (2023).

14. D. M. Copolovici, K. Langel, E. Eriste, Ü. Langel, Cell-penetrating peptides: Design, synthesis, and applications. *ACS Nano* **8**, 1972–1994 (2014).

15. S. Zhang, G. Lykotrafitis, G. Bao, S. Suresh, Size-dependent endocytosis of nanoparticles. *Adv. Mater.* **21**, 419–424 (2009).

16. Y.-Y. Yuan, C.-Q. Mao, X.-J. Du, J.-Z. Du, F. Wang, J. Wang, Surface charge switchable nanoparticles based on zwitterionic polymer for enhanced drug delivery to tumor. *Adv. Mater.* **24**, 5476–5480 (2012).

17. A. Pluen, Y. Boucher, S. Ramanujan, T. D. McKee, T. Gohongi, E. di Tomaso, E. B. Brown, Y. Izumi, R. B. Campbell, D. A. Berk, R. K. Jain, Role of tumor-host interactions in interstitial diffusion of macromolecules: Cranial vs. subcutaneous tumors. *Proc. Natl. Acad. Sci. U.S.A.* **98**, 4628–4633 (2001).

18. L. Miao, J. M. Newby, C. M. Lin, L. Zhang, F. Xu, W. Y. Kim, M. G. Forest, S. K. Lai, M. I. Milowsky, S. E. Wobker, L. Huang, The binding site barrier elicited by tumor-associated fibroblasts interferes disposition of nanoparticles in stroma-vessel type tumors. *ACS Nano* **10**, 9243–9258 (2016).

19. Y. H. Bae, K. Park, Targeted drug delivery to tumors: Myths, reality and possibility. *J. Control. Release* **153**, 198–205 (2011).

20. Q. Dai, S. Wilhelm, D. Ding, A. M. Syed, S. Sindhwani, Y. Zhang, Y. Y. Chen, P. MacMillan, W. C. W. Chan, Quantifying the ligand-coated nanoparticle delivery to cancer cells in solid tumors. *ACS Nano* **12**, 8423–8435 (2018).

21. R. J. Phillips, W. M. Deen, J. F. Brady, Hindered transport in fibrous membranes and gels: Effect of solute size and fiber configuration. *J. Colloid Interface Sci.* **139**, 363–373 (1990).

22. G. M. Thurber, M. M. Schmidt, K. D. Wittrup, Factors determining antibody distribution in tumors. *Trends Pharmacol. Sci.* **29**, 57–61 (2008).

23. R. K. Jain, Transport of molecules in the tumor interstitium: A review. *Cancer Res.* **47**, 3039–3051 (1987).

24. C. Steiger, A. Abramson, P. Nadeau, A. P. Chandrakasan, R. Langer, G. Traverso, Ingestible electronics for diagnostics and therapy. *Nat. Rev. Mater.* **4**, 83–98 (2019).

25. C. K. Schmidt, M. Medina-Sánchez, R. J. Edmondson, O. G. Schmidt, Engineering microrobots for targeted cancer therapies from a medical perspective. *Nat. Commun.* **11**, 5618 (2020).

26. E. Mathiowitz, J. S. Jacob, Y. S. Jong, G. P. Carino, D. E. Chickering, P. Chaturvedi, C. A. Santos, K. Vijayaraghavan, S. Montgomery, M. Bassett, C. Morrell, Biologically erodable microspheres as potential oral drug delivery systems. *Nature* **386**, 410–414 (1997).

27. S. S. Srinivasan, A. Alshareef, A. V. Hwang, Z. Kang, J. Kuosmanen, K. Ishida, J. Jenkins, S. Liu, W. A. M. Madani, J. Lennerz, A. Hayward, J. Morimoto, N. Fitzgerald, R. Langer, G. Traverso, RoboCap: Robotic mucus-clearing capsule for enhanced drug delivery in the gastrointestinal tract. *Sci. Robot.* **7**, eabp9066 (2022).

28. A. Abramson, E. Caffarel-Salvador, M. Khang, D. Dellal, D. Dilverstein, Y. Gao, M. R. Frederiksen, A. Vegge, F. Hubálek, J. J. Water, A. V. Friderichsen, J. Fels, R. K. Kirk, C. Cleveland, J. Collins, S. Tamang, A. Hayward, T. Landh, S. T. Buckley, N. Roxhed, U. Rahbek, R. Langer, G. Traverso, An ingestible self-orienting system for oral delivery of macromolecules. *Science* **363**, 611–615 (2019).

29. D. Son, H. Gilbert, M. Sitti, Magnetically actuated soft capsule endoscope for fine-needle biopsy. *Soft Robot.* **7**, 10–21 (2020).

30. R. H. Soon, Z. Ren, W. Hu, U. Bozuyuk, E. Yildiz, M. Li, M. Sitti, On-demand anchoring of wireless soft miniature robots on soft surfaces. *Proc. Natl. Acad. Sci. U.S.A.* **119**, e2207767119 (2022).

31. G. Arrick, D. Sticker, A. Ghazal, Y. Lu, T. Duncombe, D. Gwynne, B. Mouridsen, J. Wainer, J. P. H. Jepsen, T. S. Last, D. Schultz, K. Hess, E. M. De Alba, S. Min, M. Poulsen, C. Anker, P. Karandikar, H. D. Pedersen, J. Collins, N. E. Egecioglu, S. Tamang, C. Cleveland, K. Ishida, A. H. Uhrenfeldt, J. Kuosmanen, M. Pereverzina, A. Hayward, R. K. Kirk, S. You, C. M. Dalsgaard, S. B. Gunnarsson, I. Patsi, A. Bohr, A. Azzarello, M. R. Frederiksen, P. Herskind, J. Li, N. Roxhed, U. L. Rahbek, J. J. Water, S. T. Buckley, G. Traverso, Cephalopod-inspired jetting devices for gastrointestinal drug delivery. *Nature* **636**, 481–487 (2024).

32. K. Aran, M. Chooljian, J. Paredes, M. Rafi, K. Lee, A. Y. Kim, J. An, J. F. Yau, H. Chum, I. Conboy, N. Murthy, D. Liepmann, An oral microjet vaccination system elicits antibody production in rabbits. *Sci. Transl. Med.* **9**, eaaf6413 (2017).

33. W. Hu, G. Z. Lum, M. Mastrangeli, M. Sitti, Small-scale soft-bodied robot with multimodal locomotion. *Nature* **554**, 81–85 (2018).

34. Y. Wu, X. Dong, J. K. Kim, C. Wang, M. Sitti, Wireless soft millirobots for climbing three-dimensional surfaces in confined spaces. *Sci. Adv.* **8**, eabn3431 (2022).

35. Z. Ren, W. Hu, X. Dong, M. Sitti, Multi-functional soft-bodied jellyfish-like swimming. *Nat. Commun.* **10**, 2703 (2019).

36. J. Zhang, Z. Ren, W. Hu, R. H. Soon, I. C. Yasa, Z. Liu, M. Sitti, Voxelated three-dimensional miniature magnetic soft machines via multimaterial heterogeneous assembly. *Sci. Robot.* **6**, eabf0112 (2021).

37. S. Miyashita, S. Guitron, K. Yoshida, S. Li, D. D. Damian, D. Rus, Ingestible, controllable, and degradable origami robot for patching stomach wounds, in *Proceedings of the 2016 IEEE International Conference on Robotics and Automation (ICRA)* (IEEE, 2016), pp. 909–916.

38. F. Zhang, Z. Li, Y. Duan, A. Abbas, R. Mundaca-Uribe, L. Yin, H. Luan, W. Gao, R. H. Fang, L. Zhang, J. Wang, Gastrointestinal tract drug delivery using algae motors embedded in a degradable capsule. *Sci. Robot.* **7**, eabo4160 (2022).

39. B. Wang, K. F. Chan, K. Yuan, Q. Wang, X. Xia, L. Yang, H. Ko, Y.-X. J. Wang, J. J. Y. Sung, P. W. Y. Chiu, L. Zhang, Endoscopy-assisted magnetic navigation of biohybrid soft microrobots with rapid endoluminal delivery and imaging. *Sci. Robot.* **6**, eabd2813 (2021).

40. P. Tsvetkov, S. Coy, B. Petrova, M. Dreishpoon, A. Verma, M. Abdusamad, J. Rossen, L. Joesch-Cohen, R. Humeidi, R. D. Spangler, J. K. Eaton, E. Frenkel, M. Kocak, S. M. Corsello, S. Lutsenko, N. Kanarek, S. Santagata, T. R. Golub, Copper induces cell death by targeting lipoylated TCA cycle proteins. *Science* **375**, 1254–1261 (2022).

41. C. M. Saporito-Magrina, R. N. Musacco-Sebio, G. Andrieux, L. Kook, M. T. Orrego, M. V. Tuttolomondo, M. F. Desimone, M. Boerries, C. Borner, M. G. Repetto, Copper-induced cell death and the protective role of glutathione: The implication of impaired protein folding rather than oxidative stress. *Metallomics* **10**, 1743–1754 (2018).

42. Y. Xiao, Q. Zhai, G. Wang, X. Liu, J. Zhao, F. Tian, H. Zhang, W. Chen, Metabolomics analysis reveals heavy metal copper-induced cytotoxicity in HT-29 human colon cancer cells. *RSC Adv.* **375**, 1254–1261 (2022).

43. Y. Ogra, A. Tejima, N. Hatakeyama, M. Shiraiwa, S. Wu, T. Ishikawa, A. Yawata, Y. Anan, N. Suzuki, Changes in intracellular copper concentration and copper-regulating gene expression after PC12 differentiation into neurons. *Sci. Rep.* **6**, 33007 (2016).

44. S. M. Herd, J. Camakaris, R. Christoffeerson, P. Wookey, D. M. Danks, Uptake and efflux of copper-64 in Menkes'-disease and normal continuous lymphoid cell lines. *Biochem. J.* **247**, 341–347 (1987).

45. D. E. Goldman, Potential, impedance, and rectification in membranes. *J. Gen. Physiol.* **27**, 37–60 (1943).

46. H. Kimizuka, K. Koketsu, Ion transport through cell membrane. *J. Theor. Biol.* **6**, 290–305 (1964).

47. M. M. Schmidt, K. D. Wittrup, A modeling analysis of the effects of molecular size and binding affinity on tumor targeting. *Mol. Cancer Ther.* **8**, 2861–2871 (2009).

48. S. Ramanujan, A. Pluen, T. D. McKee, E. B. Brown, Y. Boucher, R. K. Jain, Diffusion and convection in collagen gels: Implications for transport in the tumor interstitium. *Biophys. J.* **83**, 1650–1660 (2002).

49. P. Decuzzi, F. Causa, M. Ferrari, P. A. Nett, The effective dispersion of nanovectors within the tumor microvasculature. *Ann. Biomed. Eng.* **34**, 633–641 (2006).

50. C. P. Graff, K. D. Wittrup, Theoretical analysis of antibody targeting of tumor spheroids: Importance of dosage for penetration and affinity for retention. *Cancer Res.* **63**, 1288–1296 (2003).

51. L. W. Organ, Electrophysiologic principles of radiofrequency lesion making. *Appl. Neurophysiol.* **39**, 69–76 (1976).

52. Y.-L. Xin, F.-Z. Xue, B.-S. Ge, F.-R. Zhao, B. Shi, W. Zhang, Electrochemical treatment of lung cancer. *Bioelectromagnetics* **18**, 8–13 (1997).

53. N. Jourabchi, K. Beroukhim, B. A. Tafti, S. T. Kee, E. W. Lee, Irreversible electroporation (NanoKnife) in cancer treatment. *Gastrointest. Interv.* **3**, 8–18 (2014).

54. E. D. Kirson, V. Dbaly, F. Tovarys, J. Vymazal, J. F. Soustiel, A. Itzhaki, D. Mordechovich, S. Steinberg-Shapira, Z. Gurvich, R. Schneiderman, Y. Wasserman, M. Salzberg, B. Ryffel, D. Goldsher, E. Dekel, Y. Palti, Alternating electric fields arrest cell proliferation in animal tumor models and human brain tumors. *Proc. Natl. Acad. Sci. U.S.A.* **104**, 10152–10157 (2007).

55. P. C. Valdivia, A. R. Robertson, N. K. H. De Boer, W. Marlicz, A. Koulaouzidis, An overview of robotic capsules for drug delivery to the gastrointestinal tract. *J. Clin. Med.* **10**, 5791 (2021).

56. J. Byun, M. Park, S.-M. Baek, J. Yoon, W. Kim, B. Lee, Y. Hong, K.-J. Cho, Underwater maneuvering of robotic sheets through buoyancy-mediated active flutter. *Sci. Robot.* **6**, eabe0637 (2021).

57. D. Tang, G. Kroemer, R. Kang, Targeting cuproplasia and cuproptosis in cancer. *Nat. Rev. Clin. Oncol.* **21**, 370–388 (2024).

58. Y. Liu, Y. Huo, L. Yao, Y. Xu, F. Meng, H. Li, K. Sun, G. Zhou, D. S. Kohane, K. Tao, Transcytosis of nanomedicine for tumor penetration. *Nano Lett.* **19**, 8010–8020 (2019).

59. D.-T. Nguyen, M.-J. Baek, S. M. Lee, D. Kim, S.-Y. Yoo, J.-Y. Lee, D.-D. Kim, Photobleaching-mediated charge-convertible cyclodextrin nanoparticles achieve deep tumour penetration for rectal cancer theranostics. *Nat. Nanotechnol.* **19**, 1723–1734 (2024).

60. B. Wang, J. Shen, C. Huang, Z. Ye, J. He, X. Wu, Z. Guo, L. Zhang, T. Xu, Magnetically driven biohybrid blood hydrogel fibres for personalized intracranial tumour therapy under fluoroscopic tracking. *Nat. Biomed. Eng.* **9**, 1471–1485 (2025).

61. P. Wrede, O. Degtyaruk, S. K. Kalva, X. L. Deán-Ben, U. Bozuyuk, A. Aghakhani, B. Akolpoglu, M. Sitti, D. Razansky, Real-time 3D optoacoustic tracking of cell-sized magnetic microrobots circulating in the mouse brain vasculature. *Sci. Adv.* **8**, eabm9132 (2022).

62. C. Wang, T. Wang, M. Sitti, Synthetic data-assisted miniature medical robot navigation via ultrasound imaging. *IEEE/ASME Trans. Mechatron.* **30**, 7717–7727 (2025).

63. G. H. Kim, Systematic endoscopic approach to early gastric cancer in clinical practice. *Gut Liver* **15**, 811–817 (2020).

64. T. Kaltenbach, J. C. Anderson, C. A. Burke, J. A. Dominitz, S. Gupta, D. Lieberman, D. J. Robertson, A. Shaukat, S. Syngal, D. K. Rex, Endoscopic removal of colorectal lesions—Recommendations by the US multi-society task force on colorectal cancer. *Am. J. Gastroenterol.* **115**, 435–464 (2020).

65. G. G. R. J. Johnson, R. Helewa, D. C. Moffatt, J. G. Coneys, J. Park, E. Hyun, Colorectal polyp classification and management of complex polyps for surgeon endoscopists. *Can. J. Surg.* **66**, E491–E498 (2023).

66. C. H. Huh, M. S. Bhutani, E. B. Farfán, W. E. Bolch, Individual variations in mucosa and total wall thickness in the stomach and rectum assessed via endoscopic ultrasound. *Physiol. Meas.* **24**, N15–N22 (2003).

67. Institute of Medicine, *Dietary Reference Intakes for Vitamin A, Vitamin K, Arsenic, Boron, Chromium, Copper, Iodine, Iron, Manganese, Molybdenum, Nickel, Silicon, Vanadium, and Zinc* (National Academy Press, 2001).

68. E. Uslu, V. K. Rana, Y. Guo, T. Stampoultzis, F. Gorostidi, K. Sandu, D. P. Pioletti, Enhancing robustness of adhesive hydrogels through PEG-NHS incorporation. *ACS Appl. Mater. Interfaces* **15**, 50095–50105 (2023).

69. R. Xu, W. M. Zheng, E. Martin, Acoustic holography for characterisation of sub-megahertz frequency medical ultrasound sources. *J. Acoust. Soc. Am.* **158**, 974–984 (2025).

70. G. Bianchi, P. Longhi, Copper in sea-water, potential-pH diagrams. *Corros. Sci.* **13**, 853–864 (1973).

71. M. Guan, K. Cheng, X.-T. Xie, Y. Li, M.-W. Ma, B. Zhang, S. Chen, W. Chen, B. Liu, J.-X. Fan, Y.-D. Zhao, Regulating copper homeostasis of tumor cells to promote cuproptosis for enhancing breast cancer immunotherapy. *Nat. Commun.* **15**, 10060 (2024).

72. J. Li, G. Zhang, Z. Sun, M. Jiang, G. Jia, H. Liu, N. Liu, L. Shi, L. Zhang, L. Nie, Y. Zhang, Y. Fu, Immunogenic cuproptosis in cancer immunotherapy via an in situ cuproptosis-inducing system. *Biomaterials* **319**, 123201 (2025).

73. Y. Luo, X. Luo, Y. Ru, X. Zhou, D. Liu, Q. Huang, M. Linghu, Y. Wu, Z. Lv, M. Chen, Y. Ma, Y. Huang, J. Wang, Copper(II)-based nano-regulator correlates cuproptosis burst and sequential immunogenic cell death for synergistic cancer immunotherapy. *Biomater. Res.* **28**, 0039 (2024).

74. J. Byun, Y. Lee, J. Yoon, B. Lee, E. Oh, S. Chung, T. Lee, K.-J. Cho, J. Kim, Y. Hong, Electronic skins for soft, compact, reversible assembly of wirelessly activated fully soft robots. *Sci. Robot.* **3**, eaas9020 (2018).

75. J. Byun, B. Lee, E. Oh, H. Kim, S. Kim, S. Lee, Y. Hong, Fully printable, strain-engineered electronic wrap for customizable soft electronics. *Sci. Rep.* **7**, 45328 (2017).

76. J. Byun, E. Oh, B. Lee, S. Kim, S. Lee, Y. Hong, A single droplet-printed double-side universal soft electronic platform for highly integrated stretchable hybrid electronics. *Adv. Funct. Mater.* **27**, 1701912 (2017).

77. L. Michaelis, M. L. Menten, Die Kinetik Der Invertiiiwirkulig. *Biochem. Z.* **49**, 333 (1913). [The kinetics of the inverting effect].

78. L. T. Baxter, R. K. Jain, Transport of fluid and macromolecules in tumors. I. Role of interstitial pressure and convection. *Microvasc. Res.* **37**, 77–104 (1989).

79. R. D. Shannon, Revised effective ionic radii and systematic studies of interatomic distances in halides and chalcogenides. *Acta Crystallogr.* **32**, 751–767 (1976).

80. I. Tasaki, Thermodynamic treatment of radio-tracer movements across biological membranes. *Science* **132**, 1661–1663 (1960).

81. R. N. Robertson, *The lively membranes* (Cambridge Univ. Press, 1983).

82. R. Hine, "Membrane," in *The Facts on File Dictionary of Biology* (Checkmark, ed. 3, 1999), p. 198.

83. B. J. Mossop, R. C. Barr, D. A. Zaharoff, F. Yuan, Electric fields within cells as a function of membrane resistivity—A model study. *IEEE Trans. Nanobioscience* **3**, 225–231 (2004).

84. S.-J. Hao, Y. Wan, Y.-Q. Xia, X. Zou, S.-Y. Zheng, Size-based separation methods of circulating tumor cells. *Adv. Drug Deliv. Rev.* **125**, 3–20 (2018).

85. L. J. Nugent, R. K. Jain, Pore and fiber-matrix models for diffusive transport in normal and neoplastic tissues. *Microvasc. Rev.* **28**, 270–274 (1984).

86. P. L. Paine, P. Scherr, Drag coefficients for the movement of rigid spheres through liquid-filled cylindrical pores. *Biophys. J.* **15**, 1087–1091 (1975).

87. E. A. Sykes, J. Chen, G. Zheng, W. C. W. Chan, Investigating the impact of nanoparticle size on active and passive tumor targeting efficiency. *ACS Nano* **8**, 5696–5706 (2014).

88. V. Shenoy, J. Rosenblatt, Diffusion of macromolecules in collagen and hyaluronic acid, rigid rod-flexible polymer, composite matrices. *Macromolecules* **28**, 8751–8758 (1995).

89. B. Kim, G. Han, B. J. Toley, C.-K. Kim, V. M. Rotello, N. S. Forbes, Tuning payload delivery in tumour cylindroids using gold nanoparticles. *Nat. Nanotechnol.* **5**, 465–472 (2010).

90. V. Raeesi, W. C. W. Chan, Improving nanoparticle diffusion through tumor collagen matrix by photo-thermal gold nanorods. *Nanoscale* **8**, 12524–12530 (2016).

91. Z. Qi, I. Whitt, A. Mehta, J. Jin, M. Zhao, R. C. Harris, A. B. Fogo, M. D. Breyer, Serial determination of glomerular filtration rate in conscious mice using FITC-inulin clearance. *Am. J. Physiol. Renal Physiol.* **286**, F590–F596 (2004).

92. W. M. Deen, M. J. Lazzara, B. D. Myers, Structural determinants of glomerular permeability. *Am. J. Physiol. Renal Physiol.* **281**, F579–F596 (2001).

93. N. Lindman, D. Simonsson, On the application of the shrinking core model to liquid-solid reactions. *Chem. Eng. Sci.* **34**, 31–35 (1979).

94. U. Del Monte, Does the cell number 10^9 still really fit one gram of tumor tissue? *Cell Cycle* **8**, 505–506 (2009).

95. G. P. Adams, R. Schier, A. M. McCall, H. H. Simmons, E. M. Horak, R. K. Alpaugh, J. D. Marks, L. M. Weiner, High affinity restricts the localization and tumor penetration of single-chain Fv antibody molecules. *Cancer Res.* **61**, 4750–4755 (2001).

96. T. Saga, R. D. Neumann, T. Heya, J. Sato, S. Kinuya, N. Le, C. H. Paik, J. N. Weinstein, Targeting cancer micrometastases with monoclonal antibodies: A binding-site barrier. *Proc. Natl. Acad. Sci. U.S.A.* **92**, 8999–9003 (1995).

97. A. M. Wu, W. Chen, A. Raubitschek, L. E. Williams, M. Neumaier, R. Fischer, S.-Z. Hu, T. Odom-Maryon, J. Y. Wong, J. E. Shively, Tumor localization of anti-CEA single-chain Fvs: Improved targeting by non-covalent dimers. *Immunotechnology* **2**, 21–36 (1996).

98. W. J. Aston, D. E. Hope, A. K. Nowak, B. W. Robinson, R. A. Lake, W. J. Lesterhuis, A systematic investigation of the maximum tolerated dose of cytotoxic chemotherapy with and without supportive care in mice. *BMC Cancer* **17**, 684 (2017).

99. S. N. Goldberg, Radiofrequency tumor ablation: Principles and techniques. *Eur. J. Ultrasound* **13**, 129–147 (2001).

100. C. J. Simon, D. E. Dupuy, W. W. Mayo-Smith, Microwave ablation: Principles and applications. *Radiographics* **25**, S69–S83 (2005).

101. K. Hong, C. Georgiades, Radiofrequency ablation: Mechanism of action and devices. *J. Vasc. Interv. Radiol.* **21**, S179–S186 (2010).

102. A. Kim, S. K. Lee, T. Parupudi, R. Rahimi, S. H. Song, M. C. Park, S. Islam, J. Zhou, A. K. Majumdar, J. S. Park, J. M. Yoo, B. Ziaie, An ultrasonically powered implantable microprobe for electrolytic ablation. *Sci. Rep.* **10**, 1510 (2020).

103. X. Sun, B. Yuan, W. Rao, J. Liu, Amorphous liquid metal electrodes enabled conformable electrochemical therapy of tumors. *Biomaterials* **146**, 156–167 (2017).

104. C. Ball, K. R. Thomson, H. Kavnoudias, Irreversible electroporation: A new challenge in "out of operating theater" anesthesia. *Anesth. Analg.* **110**, 1305–1309 (2010).

105. C. Ramon, P. Garguilo, E. A. Fridgeirsson, J. Haueisen, Changes in scalp potentials and spatial smoothing effects of inclusion of dura layer in human head models for EEG simulations. *Front. Neuroeng.* **7**, 32 (2014).
